# Causal association between metabolites and age-related macular degeneration: a bidirectional two-sample mendelian randomization study

**DOI:** 10.1186/s41065-024-00356-6

**Published:** 2024-12-20

**Authors:** Zhen-Yu Liu, Hang Zhang, Xiu-Li Sun, Jian-Ying Liu

**Affiliations:** Beijing Institute of Ophthalmology, Beijing Tongren Eye Center, Beijing Tongren Hospital, Capital Medical University, No. 1, Dongjiaomin Lane, Dongcheng District, Beijing, 100730 China

**Keywords:** Metabolites, Age-related macular degeneration, Causal association, Mendelian randomization, Single nucleotide polymorphism

## Abstract

**Background:**

Age-related macular degeneration (AMD) is the leading cause of visual impairment in the elderly population. Accumulating evidence has revealed the possible association between metabolites and AMD. This study aimed to assess the effect of plasma metabolites on AMD and its two subtypes using a bidirectional two-sample Mendelian randomization approach.

**Methods:**

The causality between plasma metabolites and AMD was assessed by a bidirectional two-sample Mendelian randomization (MR) analysis using the genome-wide association studies (GWAS) summary statistics of 1400 genetically determined metabolites (GDMs) and AMD. For this MR analysis, inverse variance weighted (IVW) was used as the primary method, with weighted median, MR-Egger, weighted mode, and simple mode as supplementary methods to examine the causality. MR-Egger intercept, Cochran’s Q, and MR-PRESSO test were employed to evaluate possible pleiotropy and heterogeneity.

**Results:**

The results of IVW showed significant causal associations between 13 GDMs and AMD. 1-stearoyl-GPE (18:0), androstenediol (3β,17β) monosulfate, stearoyl sphingomyelin (d18:1/18:0), xylose, and X-11,850 exhibited a protective effect on AMD, while gulonate and mannonate increased the risk of AMD. 1-stearoyl-GPE (18:0) and X-11,850 exhibited protective effects on dry AMD. DHEAS, 1-stearoyl-GPE (18:0), 5α-androstan-3β,17β-diol disulfate, xylose, androstenediol (3β,17β) monosulfate, and N2-acetyl, N6, N6-dimethyllysine exhibited a protective effect on wet AMD, while succinimide, 16a-hydroxy DHEA 3-sulfate, and X-13,553 increased the risk of wet AMD. Horizontal pleiotropy and heterogeneity did not distort the causal estimates. In the reverse MR analysis, AMD reduced the androstenediol (3β,17β) monosulfate level, and increased the stearoyl sphingomyelin(d18:1/18:0) level.

**Conclusion:**

This study supported the effect of plasma metabolites on AMD, providing novel insights for clinical diagnosis and prevention strategy.

**Supplementary Information:**

The online version contains supplementary material available at 10.1186/s41065-024-00356-6.

## Background

Age-related macular degeneration (AMD) is the leading cause of visual impairment in the elderly population and an important public issue [1, 2]. The number of AMD patients is expected to reach 288 million in 2040 globally [3]. The exact pathogenesis of AMD remains indistinct, and it is considered to be related to multiple factors. Genetic and environmental factors are related to the pathogenesis of AMD, including smoking, dietary supplementation, physical activity, serum cholesterol, hemodynamics, and circadian rhythm [1, 4, 5]. Advanced AMD is generally categorized into an atrophic subtype (known informally as dry AMD) and a neovascular subtype (known informally as wet AMD) [1]. Because of the formation of neovascularization and subsequent bleeding or leakage, wet AMD is typically associated with more severe visual impairment compared with dry AMD [4–7]. The diagnosis of AMD mainly depends on ophthalmic imaging technology to a great extent. Early symptoms of AMD may be mild and easily overlooked, so many patients may not be diagnosed in time until the disease is serious. For advanced AMD, the existing treatment strategies are not effective [8]. Therefore, it is urgent to find potential biomarkers and prevention targets for AMD.

As the final products of enzymatic processes, metabolites could reflect physiological responses from cell to tissue and organ [9]. They acted as novel diagnostic biomarkers in various diseases such as osteoporosis, anxiety disorder, and cancer [10]. Previous studies reported the alteration of metabolites in ocular diseases, including Vogt-Koyanagi-Harada syndrome, retinal vascular occlusion, diabetic retinopathy, and AMD [10]. For instance, L- gulonate NAD(+)-3-oxidoreductase and L-iditol-NAD(+)-5-oxidoreductase were found in bovine and rat lens that were associated with diabetic complications, including diabetic retinopathy [11]. Increasing studies indicated that lipid-related metabolites and plasma metabolites are associated with AMD [12, 13]. A randomized controlled trial revealed the difference in plasma metabolites between AMD patients and controls. Differential metabolites included di- and tripeptides, bile acids, covalently modified amino acids, and vitamin D-related metabolites [14]. Another randomized controlled trial suggested the association between cystine and AMD [15]. A meta-analysis indicated the potential connection between cholesteryl ester transfer and AMD [16]. However, the evidence of exact causality between metabolites and AMD is limited.

Mendelian randomization (MR) is a data analysis technique used to evaluate causal inference in epidemiological studies. As an innovative method, MR is to assess the causal association between exposure and diseases using genetic variants that have a strong association with exposure factors as instrumental variables (IVs) [17]. Because of the independent random distribution of genotypes during meiosis, the association between genetic variants and outcome remains unaffected by confounding factors, thus supporting a rational causal inference [18]. MR has been extensively applied to explore the causality between metabolites and diseases [19–22]. We performed this study to explore the effect of plasma metabolites on AMD and its two subtypes by a two-sample MR analysis. We aimed to enhance deduction regarding the influence of plasma metabolites on AMD and discover promising novel biomarkers for AMD.

## Methods

### Study design

**Fig. 1:**
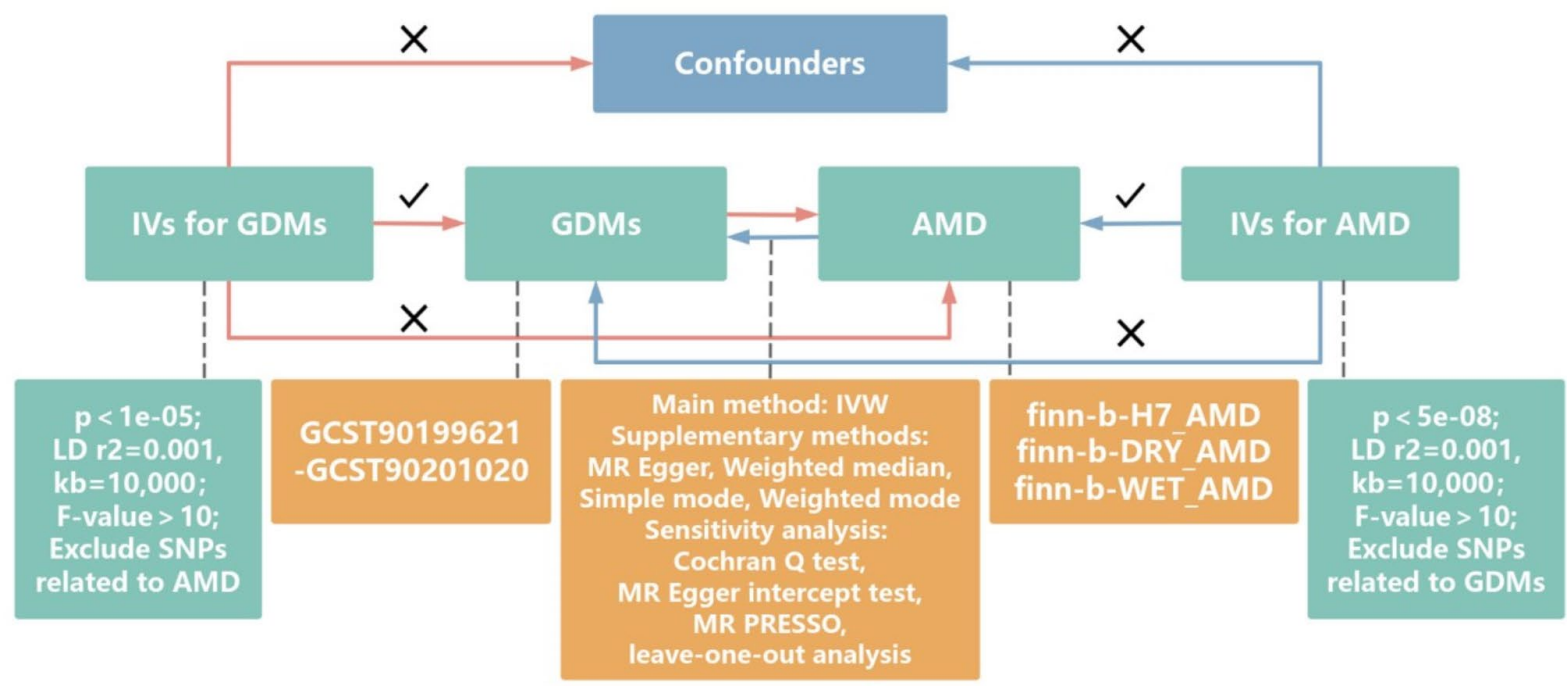
Flow chart of our study and assumptions of MR analysis. Relevance assumption: the IVs for MR analysis must be strongly associated with the exposure; Exclusion restriction assumption: the IVs can only affect the outcome through exposure; Independence assumption: the IVs are independent from any confounding factors. MR: mendelian randomization; IV: instrumental variables; IVW: inverse variance weighted; GDMs: genetically determined metabolites; SNPs: single nucleotide polymorphisms; AMD: age-related macular degeneration

We conducted a bidirectional two-sample MR analysis to estimate the causal association between 1400 genetically determined metabolites (GDMs) and AMD and its two subtypes using public summary statistics of genome-wide association study (GWAS). The GWAS of AMD we used was derived from the FinnGen. The published data of FinnGen has been through quality control, with the quality control information on the website (https://finngen.gitbook.io). Figure 1 shows a brief flow chart of this bidirectional MR design between metabolites and AMD. This MR study met the STROBE-MR guidelines [17] and three core assumptions [23, 24]: [1] The IVs must be associated strongly with exposure; [2] The IVs only affect the outcome through exposure; [3] The IVs are independent from any confounding factors (Fig. 1). The MR analysis avoided the five pitfalls proposed by Burgess and co-workers [25]. All analyses in our study were based on publicly available summary data, no additional ethical approval or informed consent was required.

### Data sources

Summary statistics for GDMs were obtained from GWAS studies conducted by Chen et al. [26] from the Canadian Longitudinal Study on Aging (CLSA) cohort, with a large available plasma metabolites sample size including 1091 metabolites and 309 metabolite ratios from 8299 European individuals. The datasets for GDMs were released to the public in 2023 and are available in the GWAS catalog (https://www.ebi.ac.uk/gwas/, the accession number: GCST90199621-90201020).

Summary statistics for AMD and subtypes were extracted from the public online database FinnGen consortium [27] (https://www.finngen.fi/) using the keywords AMD and subtype. The dataset of AMD included 3,763 cases and 205,359 controls of European ancestry, covering 16,380,424 single nucleotide polymorphisms (SNP). We also obtained two separate subtype datasets of AMD. Quality control was also mentioned in the IEUOpenGWAS project (https://mrcieu.github.io/ieugwasr/articles/guide.html). The GWAS IDs include finn-b-H7_AMD, finn-b-DRY_AMD, and finn-b-WET_AMD. Details of the GWAS are shown in Supplementary Table 1. After data processing and quality control, GWAS was performed using the fastGWA tool from GCTA version 1.93.2 beta, adjusting for age, sex, hour since the last meal or drink, genotyping batch, and the first ten genetic principal components.

### Selection of instrumental variables

Our selection of instrumental variables adheres to the STROBE-MR guidelines and the three core assumptions of Mendelian randomization. Eligible SNPs meeting the following criteria were screened out as genetic IVs. Firstly, to ensure the robustness of our analysis, independent SNPs that were strongly related to metabolites were filtered with *p* < 1e-05 to obtain an appropriate number of SNPs [26, 28, 29]. Secondly, the SNPs in strong linkage disequilibrium were excluded by clumping procedure (R^2^ < 0.001 within 10,000 kb window distance) [30]. We calculated the F-value for each SNP using the formula: F = R^2^(N-k-1) / k(1-R^2^). R^2^ represented the capacity of SNP to explain exposure factors. The SNPs with an F-value less than 10 were excluded to avoid bias from weak IVs. For inexistent SNPs in outcome data, we searched for other SNPs as proxies according to R^2^ > 0.8. LD trait online Tool (https://ldlink.nih.gov/) was used to screen SNPs associated with known risk factors of AMD (high-density lipoprotein cholesterol, body mass index, diabetes, etc.) [31, 32]. The confounding SNPs were eliminated before the final MR analysis. In reverse MR analysis, we used *p* < 5e-08 to select IVs for AMD and AMD subtypes.

### MR analysis

In this study, random-effect inverse variance weighted (IVW) was used as the primary analytical method for MR analysis to infer the causal association between GDMs and AMD [33]. MR-Egger, weighted median, weighted mode, and simple mode were used as supplementary analytical methods to verify the robustness of IVW results [33, 34]. IVW analysis provided an accurate estimate in the absence of horizontal pleiotropy. MR-Egger identified the possible pleiotropy of IVs through the intercept term and provided a consistent result with IVW when the intercept was zero or close to zero [24, 35]. Weighted median was considered a reliable method of causality estimation when less than 50% of IVs were invalid or affected by pleiotropy [36]. A reverse MR analysis was also conducted to assess the causal effect of AMD on GDMs.

### Sensitivity analysis

Cochran’s Q test was used to detect heterogeneity, with *p* < 0.05 indicating the presence of heterogeneity [37–39][32–34]. MR-Egger intercept and MR-PRESSO global test were used for assessing horizontal pleiotropy [38, 39]. Additionally, MR-PRESSO analysis could also identify outliers. If outliers existed, we removed them and re-performed MR analysis. The influence of a single SNP on the results was assessed by leave-one-out analysis.

### Validation with additional GWAS databases

To validate our main findings, we used additional separate GWAS studies for additional forward and reverse MR analysis and sensitivity analysis. The GWAS databases used are derived from GWAS Catalog (https://www.ebi.ac.uk/gwas/), obtained in December 2024 (GWAS ID: GCST90043776 and GSCT90086108). The screening criteria for the instrumental variables used in this section are the same as in the main MR analysis, and the specific information of SNPs is obtained.

### Statistical analysis

TwoSampleMR (0.5.9) package and MRPRESSO (1.0) package in R version 4.3.2 were used for statistical analysis. *p* < 0.05 was considered as a significant difference. Further, we performed false discovery rate (FDR) correction on the *P*-values to assess the statistical significance of the associations we observed [40]. The analysis results were displayed by scatter plots, forest plots, and funnel plots. The sample size was determined based on power calculation with α value of 0.05 and β level of 0.2 for an 80% power.

## Results

### Selection of instrumental variables

Following the above selection criteria for IVs, we screened out more than 30,000 SNPs for 1400 GDMs overall. Each metabolite had at least 12 SNPs. The F-value for all SNPs was greater than 10, indicating the absence of weak IVs. After preliminary analysis and a series of quality control steps including confounding analysis (Supplementary Table 2), we used 189, 57, and 235 SNPs in MR analysis for AMD, dry AMD, and wet AMD as outcomes respectively. The SNPs for final MR analysis are shown in Supplementary Table 3. In reverse MR analysis, we used 9, 6, and 9 SNPs of three AMD datasets respectively, details of these SNPs are shown in Supplementary Table 4.

### Causal effects of metabolites on AMD and subtypes

**Fig. 2:**
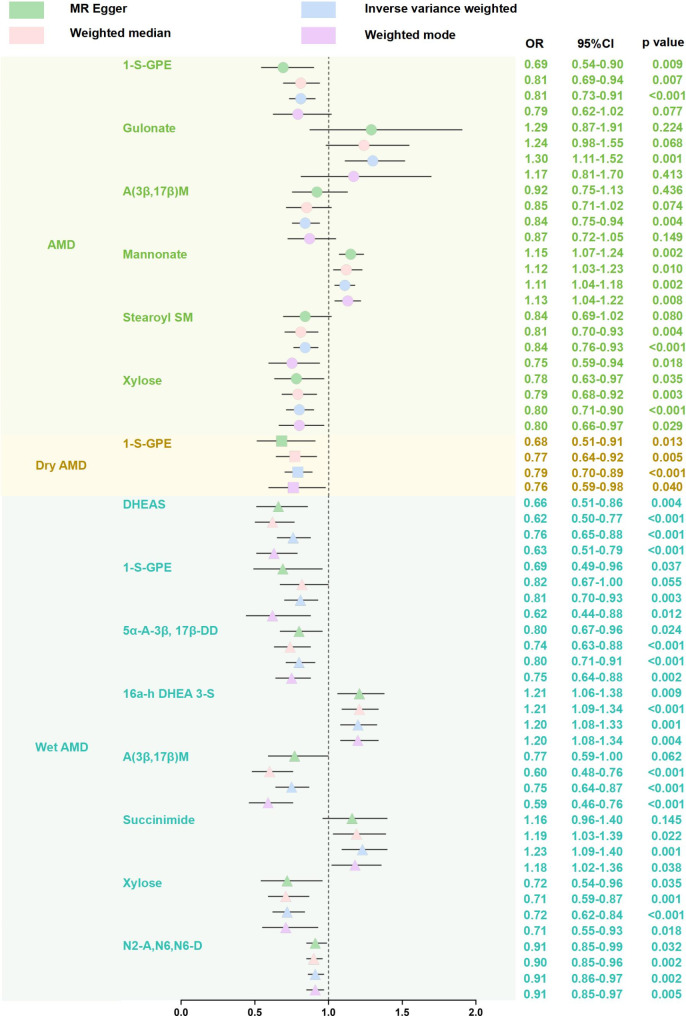
MR results for causal effects of metabolites on AMD and subtypes. We found the causal effects of several metabolites on AMD. OR: odds ratio; CI: confidence interval; AMD: age-related macular degeneration; 1-S-GPE: 1-stearoyl-GPE (18:0); A (3β,17β) M: androstenediol (3β,17β) monosulfate; Stearoyl SM: stearoyl sphingomyelin (d18:1/18:0); 5α-A-3β, 17β-DD: 5α-androstan-3β,17β-diol disulfate; 16a-h DHEA 3-S: 16a-hydroxy DHEA 3-sulfate; N2-A, N6,N6-D: N2-acetyl, N6,N6-dimethyllysine

MR results of different analytical methods are shown in Fig. 2 and Supplementary Table 5. There were significant causal associations between 13 GDMs and AMD. The results of IVW showed that gulonate (OR = 1.30, *p* = 0.001) and mannonate (OR = 1.11, *p* = 0.002) increased the risk of AMD, while 1-stearoyl-GPE (18:0) (OR = 0.81, *p* < 0.001), androstenediol (3β,17β) monosulfate (OR = 0.84, *p* = 0.004), stearoyl sphingomyelin (d18:1/18:0) (OR = 0.84, *p* < 0.001) and xylose (OR = 0.80, *p* < 0.001) decreased the risk of AMD. For two AMD subtypes, the IVW estimates indicated that 1-stearoyl-GPE (18:0) (OR = 0.79, *p* < 0.001) exhibited protective effects on dry AMD; 1-stearoyl-GPE (18:0) (OR = 0.81, *p* = 0.003), androstenediol (3β,17β) monosulfate (OR = 0.75, *p* < 0.001), xylose (OR = 0.72, *p* < 0.001), DHEAS (OR = 0.76, *p* < 0.001), 5α-androstan-3β,17β-diol disulfate (OR = 0.80, *p* < 0.001), and N2-acetyl, N6, N6-dimethyllysine (OR = 0.91, *p* = 0.002) decreased the risk of wet AMD, while 16a-hydroxy DHEA 3-sulfate (OR = 1.20, *p* = 0.001) and succinimide(OR = 1.23, *p* = 0.001) increased the risk of wet AMD. In addition, several metabolites with unknown chemical properties were found to have a causal relationship with AMD or two subtypes. Compound X-13,553 was a risk factor for wet AMD (OR = 1.36, *p* < 0.001), while compound X-11,850 was a protective factor for AMD (OR = 0.81, *p* = 0.002) and dry AMD (OR = 0.73, *p* < 0.001). Additionally, MR-Egger, Weighted median, Weighted mode, and the Simple mode methods showed consistent results with IVW (Supplementary Table 5). Scatter plots for effect sizes of SNPs for AMD and its subtypes were shown in Supplementary Fig. 1.

### Causal effects of AMD and subtypes on metabolites

According to reverse MR results, when whole AMD is taken as exposure, AMD is the protective factor of androstenediol (3β, 17β) monosulfate levels ( OR = 0.96, *p* = 0.003) and the risk factor of stearoyl sphingomyelin levels (D18: 1/18: 0) (OR = 1.03, *p* = 0.042). There were no significant causal effects of AMD and subtypes on other GDMs (Supplementary Table 6).

### Sensitivity analysis

Sensitivity analysis was used to verify the robustness of IVW results. Cochran’s Q and MR-Egger intercept test showed that there was no heterogeneity and horizontal pleiotropy in MR analysis results of the effect of metabolites on AMD and subtypes (Table 1). MR-PRESSO further verified the absence of horizontal pleiotropy and outliers (Table 1). Leave-one-out analysis indicated that the overall causal evaluations were not influenced by any single SNP. Leave-one-out analysis plots and funnel plots were presented in Supplementary Figs. 2–3. Sensitivity analysis results for reverse MR are shown in Supplementary Table 7. The causal effect of AMD on metabolites was not affected by pleiotropy except for succinimide and X-11,850.

**Table 1:** Results of sensitivity analysis

Outcome	Exposure	Method	Cochran’s Q		MR-Egger intercept		MR-PRESSO Global	
			Q statistics	*p*	intercept	*p*	RSSobs	*p*
AMD	1-S-GPE	Inverse variance weighted	40.18	0.18	0.02	0.191	44.82	0.162
		MR Egger	38.06	0.21				
	Gulonate	Inverse variance weighted	20.06	0.45	0.00	0.963	21.97	0.490
		MR Egger	20.06	0.39				
	A(3β,17β)M	Inverse variance weighted	18.55	0.95	-0.01	0.290	19.31	0.960
		MR Egger	17.38	0.96				
	Mannonate	Inverse variance weighted	19.37	0.43	-0.02	0.067	24.57	0.501
		MR Egger	15.58	0.62				
	Stearoyl SM	Inverse variance weighted	33.00	0.52	0.00	0.963	35.68	0.501
		MR Egger	33.00	0.47				
	Xylose	Inverse variance weighted	11.31	0.99	0.00	0.780	11.80	0.990
		MR Egger	11.23	0.98				
	X-11,850	Inverse variance weighted	13.00	0.93	-0.01	0.654	14.28	0.945
		MR Egger	12.80	0.92				
Dry AMD	1-S-GPE	Inverse variance weighted	26.55	0.78	0.02	0.278	28.88	0.773
		MR Egger	25.33	0.79				
	X-11,850	Inverse variance weighted	15.65	0.83	-0.02	0.452	17.50	0.818
		MR Egger	15.07	0.82				
Wet AMD	DHEAS	Inverse variance weighted	54.98	0.10	0.02	0.236	58.41	0.092
		MR Egger	53.15	0.12				
	1-S-GPE	Inverse variance weighted	40.04	0.19	0.02	0.311	44.58	0.160
		MR Egger	38.76	0.19				
	5α-A-3β, 17β-DD	Inverse variance weighted	27.61	0.43	0.00	0.921	32.04	0.435
		MR Egger	27.60	0.38				
	16a-h DHEA 3-S	Inverse variance weighted	26.70	0.18	0.00	0.799	28.20	0.332
		MR Egger	26.61	0.15				
	A(3β,17β)M	Inverse variance weighted	27.49	0.60	0.00	0.826	31.19	0.562
		MR Egger	27.44	0.55				
	Succinimide	Inverse variance weighted	31.30	0.05	0.02	0.399	35.06	0.087
		MR Egger	30.12	0.05				
	Xylose	Inverse variance weighted	12.06	0.98	0.00	0.995	12.64	0.984
		MR Egger	12.06	0.97				
	X-13,553	Inverse variance weighted	19.51	0.67	0.00	0.868	20.67	0.716
		MR Egger	19.48	0.62				
	N2-A, N6,N6-D	Inverse variance weighted	22.97	0.46	0.00	0.995	25.34	0.507
		MR Egger	22.97	0.40				

AMD: age-related macular degeneration; 1-S-GPE: 1-stearoyl-GPE (18:0); A (3β,17β) M: androstenediol (3β,17β) monosulfate; Stearoyl SM: stearoyl sphingomyelin (d18:1/18:0); 5α-A-3β, 17β-DD: 5α-androstan-3β,17β-diol disulfate; 16a-h DHEA 3-S: 16a-hydroxy DHEA 3-sulfate; N2-A, N6,N6-D: N2-acetyl, N6,N6-dimethyllysine

### Validation results from additional GWAS databases

When 13 GDMs were used as exposure, we found that succinimide increased the risk of AMD (OR = 1.33, *p* = 0.008), while DHEAS was a protective factor for AMD (OR = 0.87, *p* = 0.036). In the reverse MR analysis, we found that AMD increased the levels of stearoyl sphingomyelin (d18:1/18:0) (OR = 1.03, *p* = 0.028). The MR results are presented in the Supplementary Table 8. We also performed sensitivity analysis and did not find any presence of heterogeneity or pleiotropy. The results of sensitivity analysis are provided in Supplementary Table 9.

## Discussion

The pathogenesis of AMD is unclear and metabolites have shown a possible association with AMD, but the causal relationship is less clear. Evidence of randomized controlled trials (RCT) is lacking. Previous MR studies took metabolites as exposure and diseases as outcomes and obtained some possible causal relationships [41, 42]. Based on the lack of knowledge in the current literature and clinical needs, we chose this research question, without selecting for exposure factors impractical or unrelated to genetic variants as mentioned in the article by Burgess et al. [25], for example, the use of chopsticks. Besides, how our study avoided the five “pitfalls” they have highlighted were displayed in supplementary materials about five pitfalls.

In our study, we systematically evaluated the causal effect of plasma metabolites on AMD and its two subtypes based on the GWAS of an external dataset [26]. This assumption requires that the instrumental variables (IVs) must be strongly associated with the exposure. In our study, we selected SNPs associated with 1400 genetically determined metabolites (GDMs), using a criterion of p-value < 1e-05 to ensure strong relevance between the IVs and the exposure. A total of 5 GDMs including gulonate, mannonate, 16a-hydroxy DHEA 3-sulfate, succinimide and X-13,553 would increase the risk of AMD, while 8 GDMs including 1-stearoyl-GPE (18:0), androstenediol (3β,17β) monosulfate, stearoyl sphingomyelin (d18:1/18:0), xylose, DHEAS, 5α-androstan-3β, 17β-diol disulfate, N2-acetyl, N6, N6-dimethyllysine, and X-11,850 exhibited protective effects on AMD. Among these metabolites, 1-stearoyl-GPE (18:0) exhibited protective effects both on AMD and its two subtypes. Sensitivity analysis demonstrated the robustness of our analysis (Table 1). In the validation of the above results using additional GWAS studies, we found that succinimide increased the risk of AMD, while DHEAS was a protective factor for AMD. Consistently, previous studies pointed the metabolites are closely connected to AMD [43, 44]. For instance, a recent mendelian randomization study based on one dataset al.so indicated that 1-stearoyl-GPE (18:0), androstenediol (3 beta, 17 beta) disulfate [2], and 1-palmitoyl-2-docosahexaenoyl-GPE (16:0/22:6) had causal effects on AMD [45]. These data identified specific metabolites that are differentially expressed in AMD patients, which might serve as potential biomarkers for the disease.

Previous studies have indicated the relationship between these metabolites and various diseases. For example, gulonate was related to kidney function by untargeted metabolomics profiling of blood samples [46]. Mannonate is significantly correlated with diabetes-related glycemic indices and has been regarded as a possible biomarker for type 2 diabetes [47]. Androstenediol (3β,17β) monosulfate had significant changes in patients with rheumatoid arthritis [48]. 16a-hydroxy-DHEA-3-sulfate, biochemically related to DHEA, estradiol, and estrone, is a kind of steroid metabolite involved in the risk of breast cancer [49]. We revealed the role of these metabolites on AMD and further confirmed the involvement of related metabolic pathways in human diseases.

Our results showed the protective effect of 1-stearoyl-GPE(18:0) on AMD and two subtypes. A previous study suggested that 1-stearoyl-GPE(18:0) has a role in increasing insulin sensitivity and decreasing body mass index (BMI) and alanine aminotransferase (ALT) [50]. This phenomenon indicated a consistent idea with our results, as diabetes and BMI have a bearing on the risk of AMD [51]. Feofanova et al. [52] reported similar results that besides 1-stearoyl-GPE(18:0), another saturated lysophosphatidylethanolamine 1-palmitoyl-GPE also exhibited a protective effect on AMD. These results prompt the potential of saturated lysophosphatidylethanolamine species on clinical application for AMD prevention [50, 52, 53].

Glycerophospholipids and sphingolipids are major constituents of membrane lipid bilayers [54]. Bioactive lipid mediators generated from membrane lipids play a key role in various biological processes, including angiogenesis [55–57]. Neovascularization is a characteristic sign of wet AMD, and the latest research has revealed the effect of sphingolipids on ocular neovascularization [58, 59]. Interestingly, we found a bidirectional causal correlation between stearoyl sphingomyelin (d18:1/18:0) and AMD. Stearoyl sphingomyelin (d18:1/18:0) showed a protective effect on AMD while the presence of AMD would increase the level of stearoyl sphingomyelin (d18:1/18:0). This association deserves to be further studied to reveal the mechanism of sphingomyelin in ocular neovascularization.

DHEA is the precursor of sex hormones, and it’s the most abundant steroid hormone in human body [60]. With the aging of organisms, the decrease of DHEA synthesis would lead to a large consumption of estrogen and androgen [61–63]. DHEAS is the sulfate form of DHEA in plasma. DHEA and DHEAS have been evaluated as potential anti-aging therapies, although in the absence of convincing clinical trials [64, 65]. A cross-sectional study indicated a significant reduction in serum DHEAS in both exudative and nonexudative AMD patients [66]. However, some studies revealed the opposite results [67, 68]. In our MR study, DHEAS showed a protective effect on wet AMD. It has been proved in vitro that DHEAS could protect RPE from oxidative stress induced by hydrogen peroxide, and this effect is mediated by sigma1 receptors [69]. DHEA has the effect of improving arterial circulation through anti-oxidant and anti-inflammatory functions [70, 71]. Ocular microcirculation impairment and decreased choroidal perfusion are thought to accelerate RPE dysfunction, geographic atrophy, and neovascularization in AMD patients [72]. This may explain the protective mechanisms of DHEAS against AMD. Our results indicated that DHEAS may be used for the prevention or treatment of AMD in the future.

Our results showed xylose may play a protective role on whole AMD and wet AMD. Xylose participates in multiple metabolic processes [73–75], including the regulation of blood glucose and accelerating diabetic retinopathy and cataracts [11, 76]. Experiments in vitro developed an efficient biotechnological method to convert xylose into vitamin A, an essential nutrient for maintaining the normal physiology of retina [77]. However, the specific mechanism of xylose in AMD needs to be explored. Additionally, alterations of amino acid levels have been found in retinopathy, particularly diabetic retinopathy and AMD [78, 79]. Our results also suggested a protective role of N2-acetyl, N6, N6-dimethyllysine on AMD. It suggests the application prospect of amino acids in clinical prevention and treatment of AMD [80].

This study presents several strengths. We used the latest large-scale metabonomic genetic datasets comprising numerous categories of metabolites for a comprehensive and systematic MR analysis. The inherent advantages of MR avoid most of the confounding factors and biases effectively. We also considered two subtypes of AMD to provide more comprehensive insights into the role of plasma metabolites on AMD. Reverse MR analysis was further conducted to assess the reverse causal effect. Our research has promoted the application of metabonomics in AMD. Further investigation into the mechanisms of action of these substances in AMD could be a feasible direction for future research.

This study also presents some limitations. First, given that our dataset is predominately focused on European samples, the applicability of our results to other ethnicities is still in question, which emphasizes the requirement for subsequent research to address a wider demographic variety [26]. Before these metabolites can be used in clinical practice, they need to be validated in larger, independent cohorts to ensure their sensitivity and specificity for AMD patients. Second, due to the limitations of the GWAS database, we couldn’t stratify AMD by other factors, such as biological rhythm, gender, and disease stage of patients. Third, most of the plasma metabolites could not penetrate the blood-ocular barrier, so the ability of plasma metabolites to reflect AMD conditions needs to be further explored. Last but not least, we cannot reveal the relationship between metabolites and AMD at the individual level. Some of the associations in our main findings were not validated in external data and possible reasons include sample size, genetic background, environmental factors or other variables that may influence the outcome. Further clinical trials and basic experiments are required to verify our results.

## Conclusion

In conclusion, our study investigated the bidirectional causal association between metabolites and AMD. The results supported the effects of 13 plasma metabolites on AMD and provided new insights for the diagnosis and prevention of AMD. It is necessary to further explore the application of these metabolites as potential biomarkers or therapeutic targets for AMD.

## Electronic Supplementary Material

Below is the link to the electronic supplementary material.

Supplementary Material 1

Supplementary Material 2

Supplementary Material 3

Supplementary Material 4

Supplementary Material 5

Supplementary Material 6

Supplementary Material 7

Supplementary Material 8

Supplementary Material 9

Supplementary Material 10

Supplementary Material 11

Supplementary Material 12

Supplementary Material 13

Supplementary Material 14

## Data Availability

The datasets generated during and analyzed during the current study are available from the corresponding author on reasonable request. The analysis codes were shown in supplementary materials.
