# Supplementary figures and images for: Causal association between metabolites and age-related macular degeneration: a bidirectional two-sample mendelian randomization study
Source: Hereditas. 2024 Dec 20;161:51. doi: 10.1186/s41065-024-00356-6 (PMC11662531; doi:10.1186/s41065-024-00356-6)

Supplementary Figure 1. Scatter plots of MR analysis.

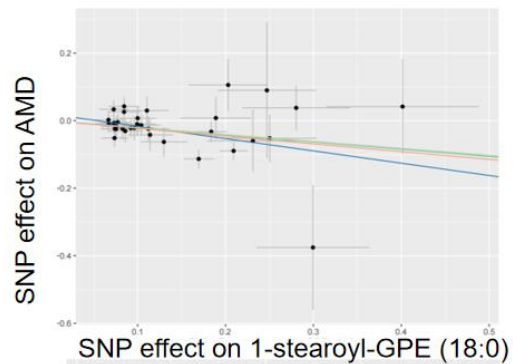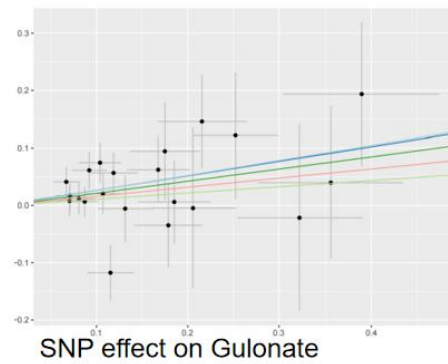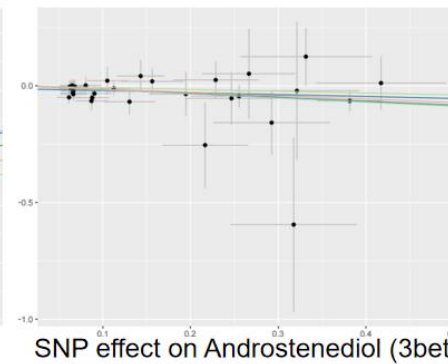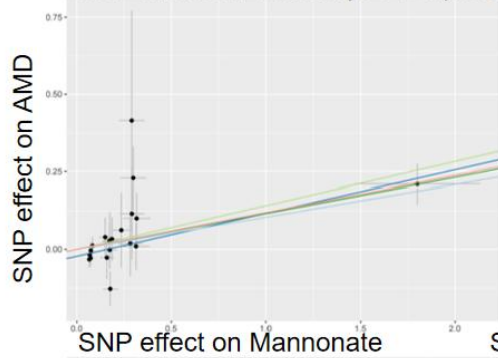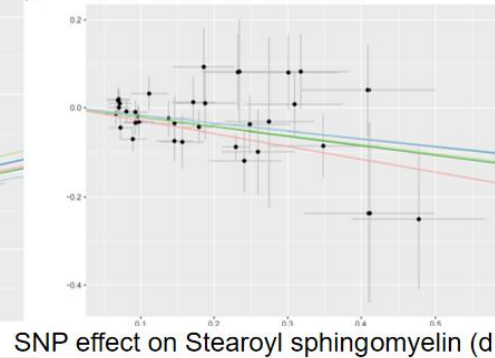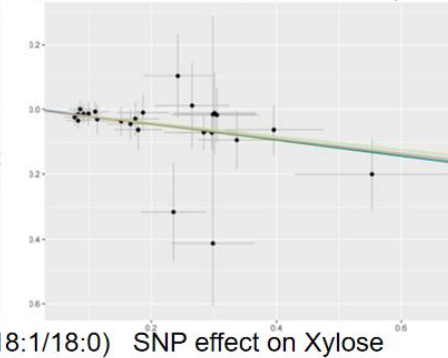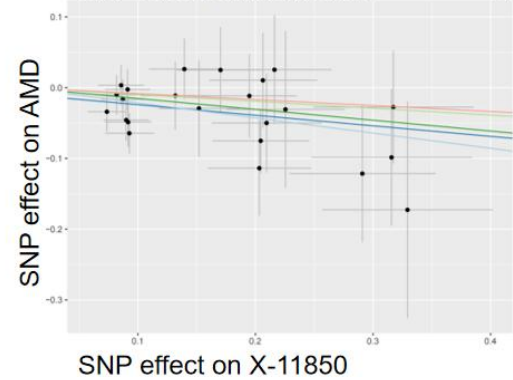

### MR Test

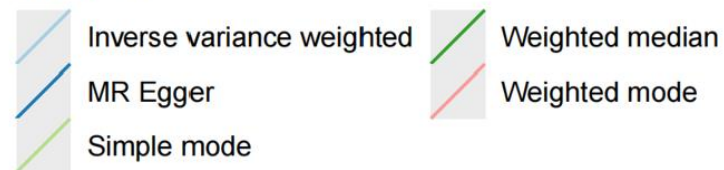

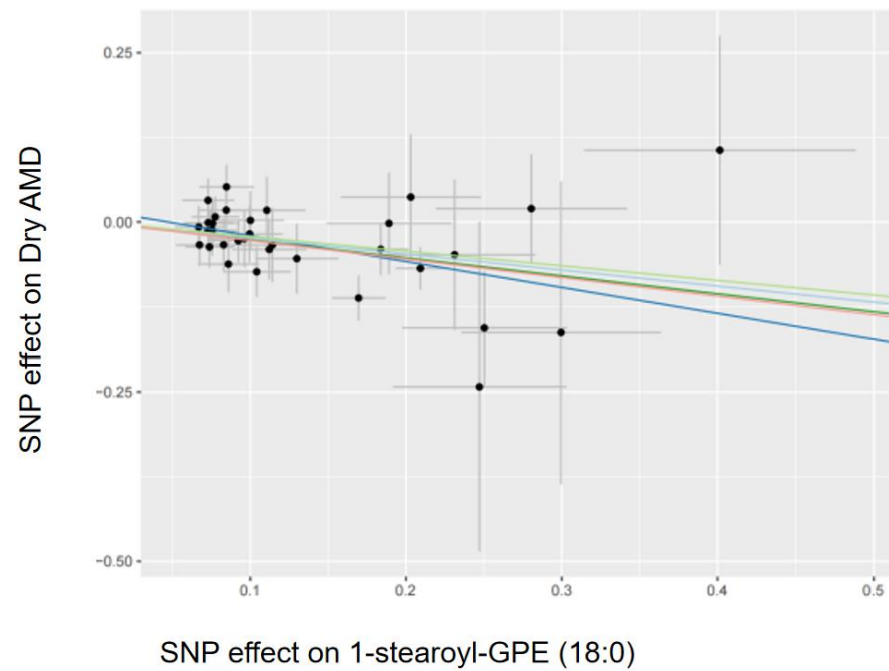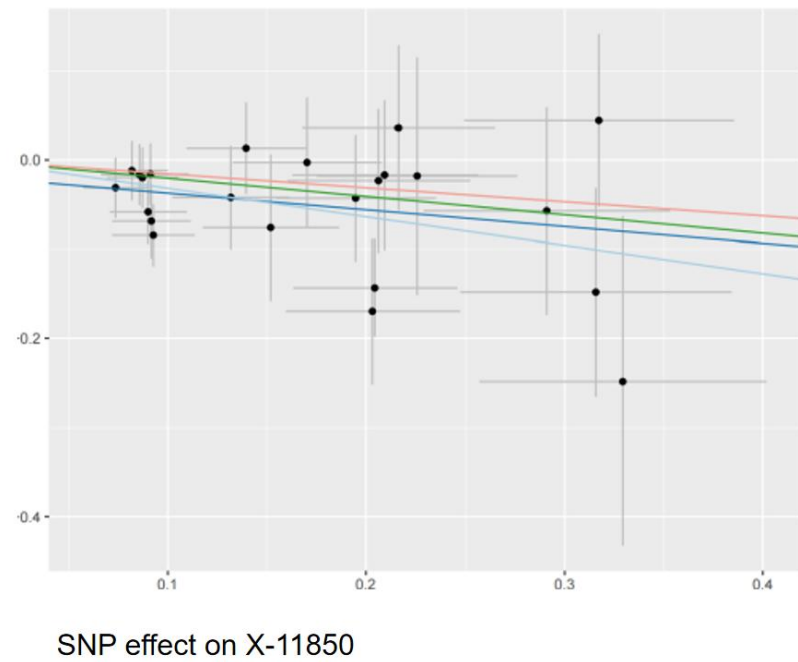

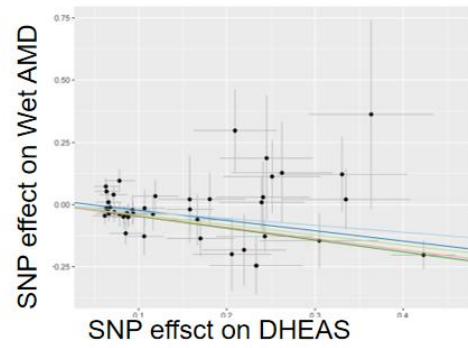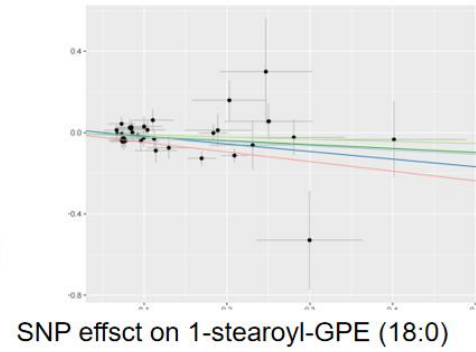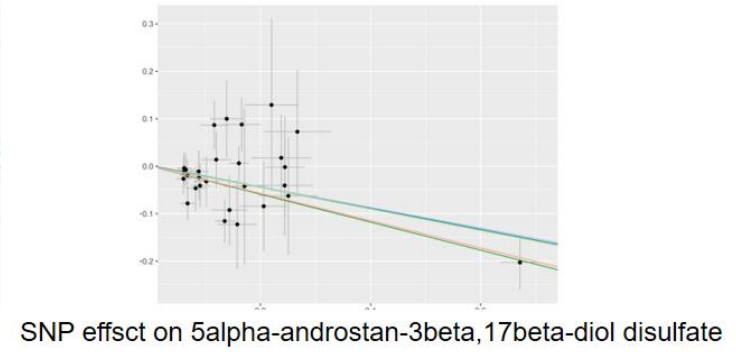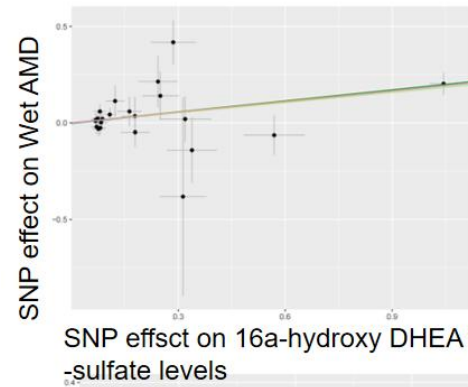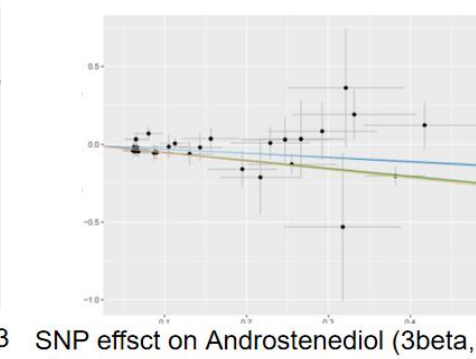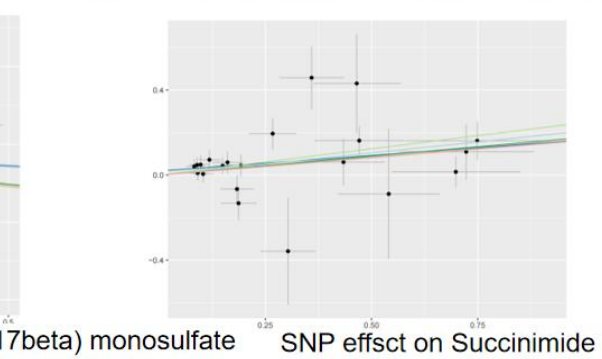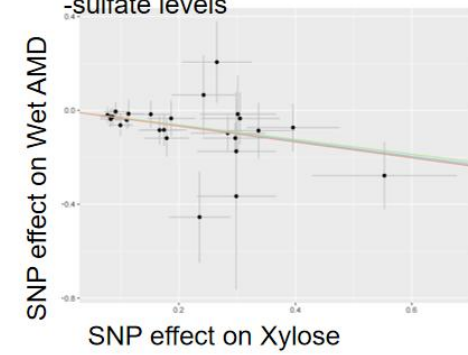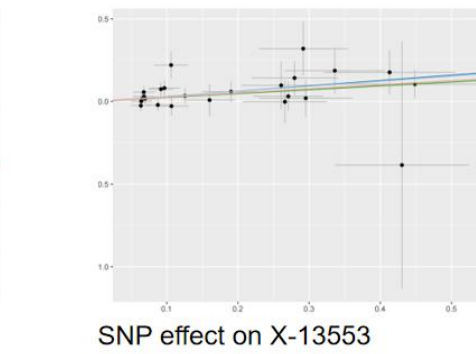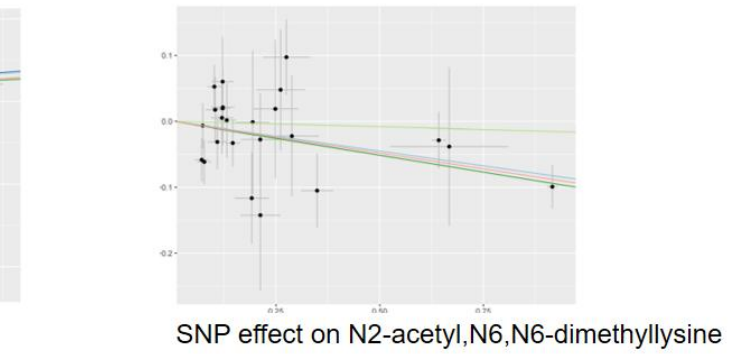

Supplement: Supplementary file 1 — Supplementary Material 1 [file 41065_2024_356_MOESM1_ESM.pdf]
