# Supplementary material for: Causal association between metabolites and age-related macular degeneration: a bidirectional two-sample mendelian randomization study
Source: Hereditas. 2024 Dec 20;161:51. doi: 10.1186/s41065-024-00356-6 (PMC11662531; doi:10.1186/s41065-024-00356-6)
Supplement: Supplementary file 2 — Supplementary Material 2 [file 41065_2024_356_MOESM2_ESM.pdf]

Supplementary Figure 2. Leave-one-out analysis plots of MR analysis.

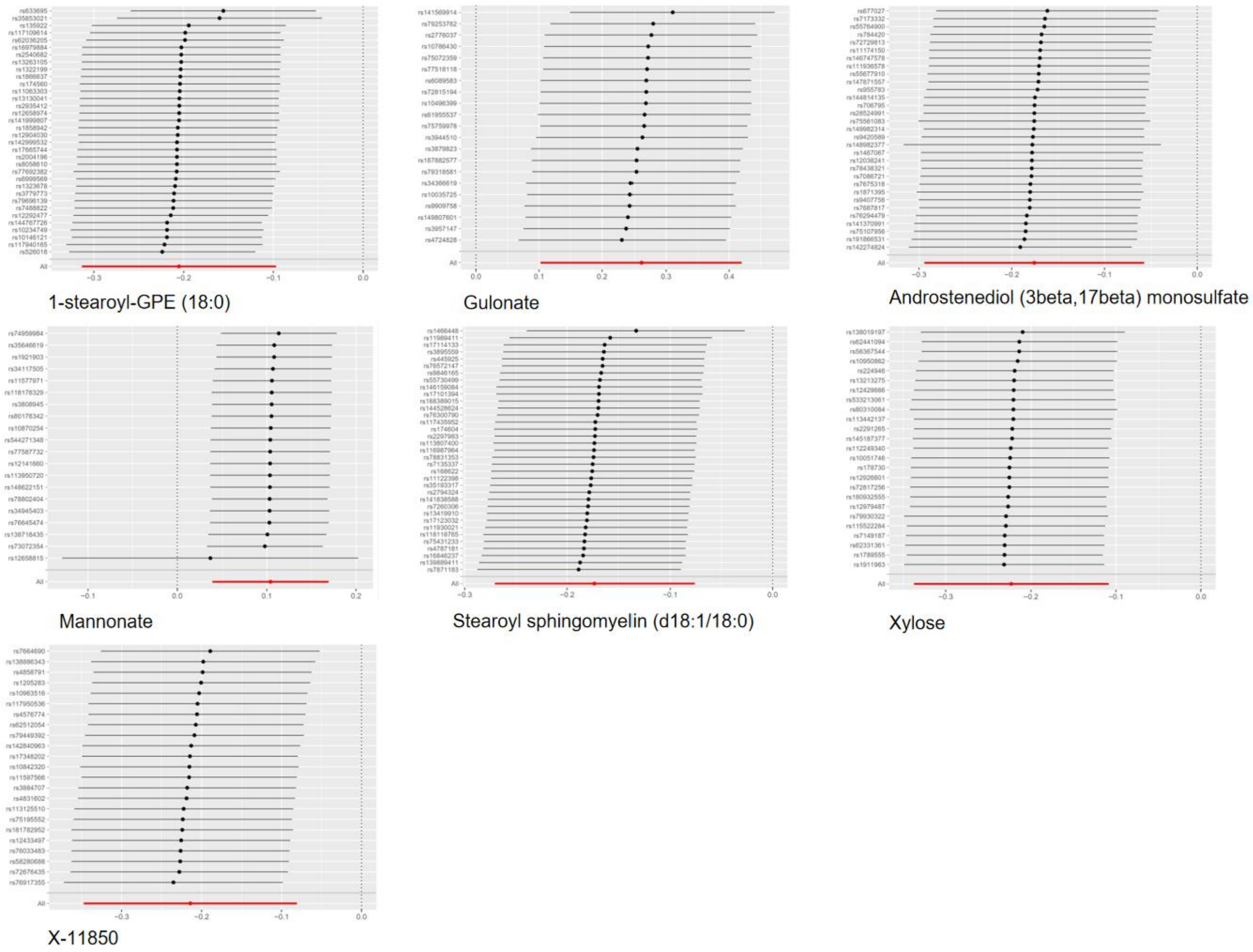

Leave-one-out analysis plots of MR analysis between metabolites and AMD

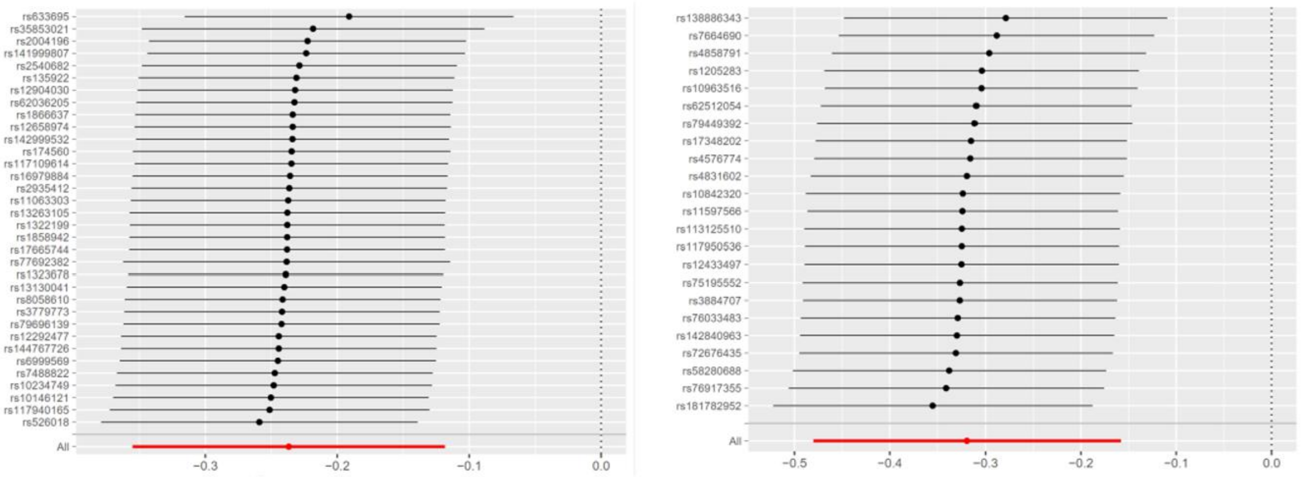

1-stearoyl-GPE (18:0) X-11850

Leave-one-out analysis plots of MR analysis between metabolites and dry AMD

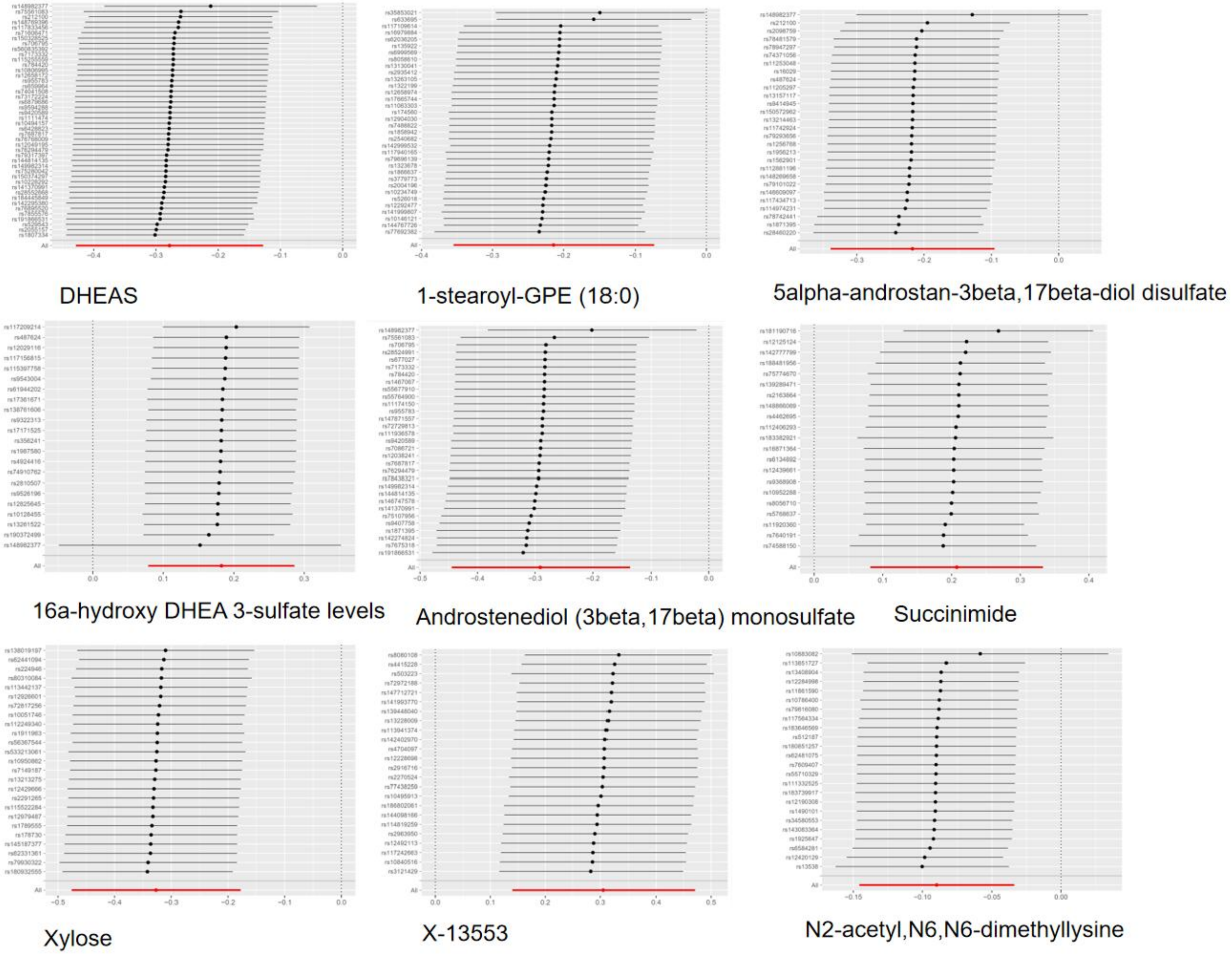

Leave-one-out analysis plots of MR analysis between metabolites and wet AMD
