# Supplementary material for: Causal association between metabolites and age-related macular degeneration: a bidirectional two-sample mendelian randomization study
Source: Hereditas. 2024 Dec 20;161:51. doi: 10.1186/s41065-024-00356-6 (PMC11662531; doi:10.1186/s41065-024-00356-6)
Supplement: Supplementary file 3 — Supplementary Material 3 [file 41065_2024_356_MOESM3_ESM.pdf]

Supplementary Figure 3. Funnel plots of MR analysis.

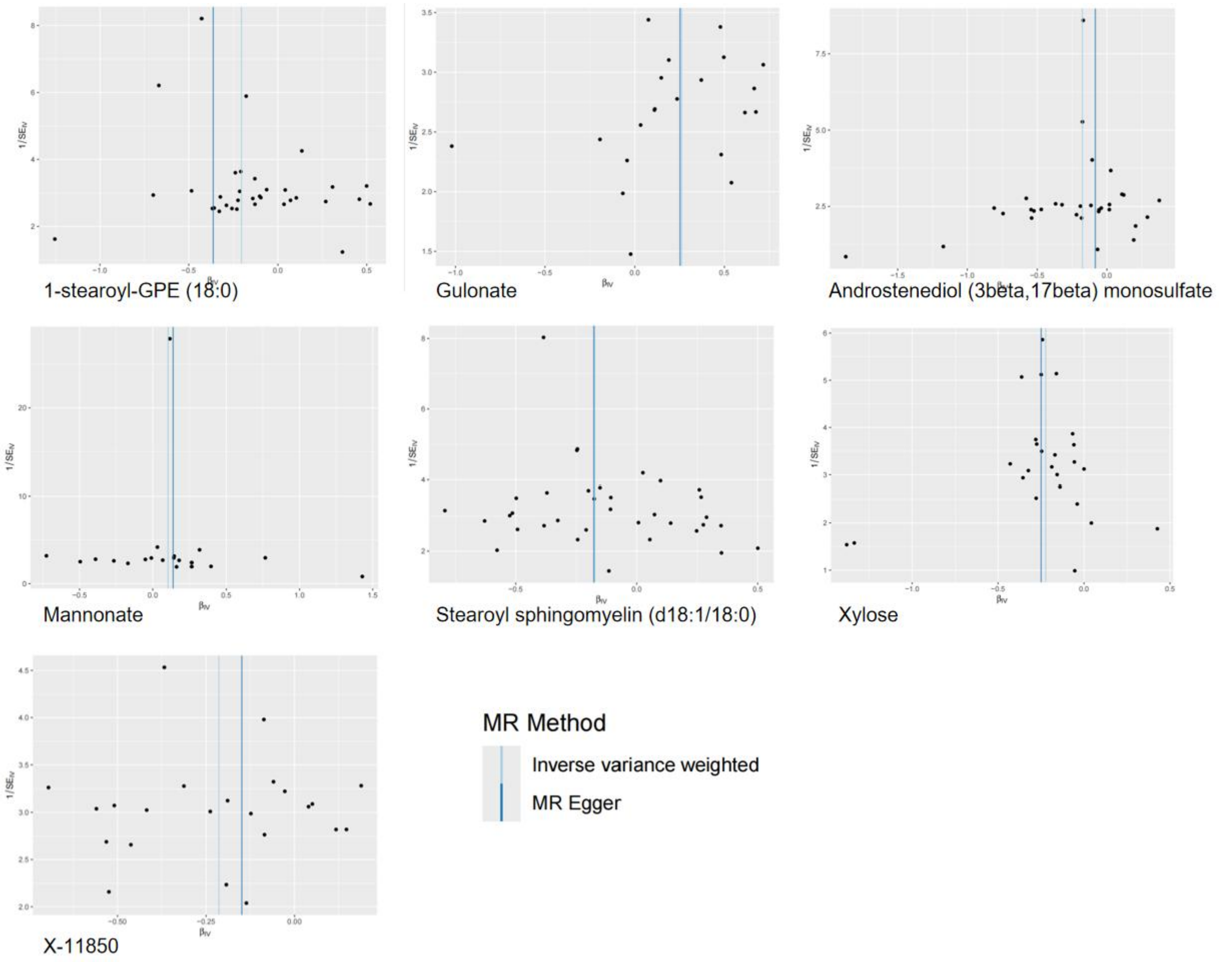

Funnel plots of MR analysis between metabolites and AMD

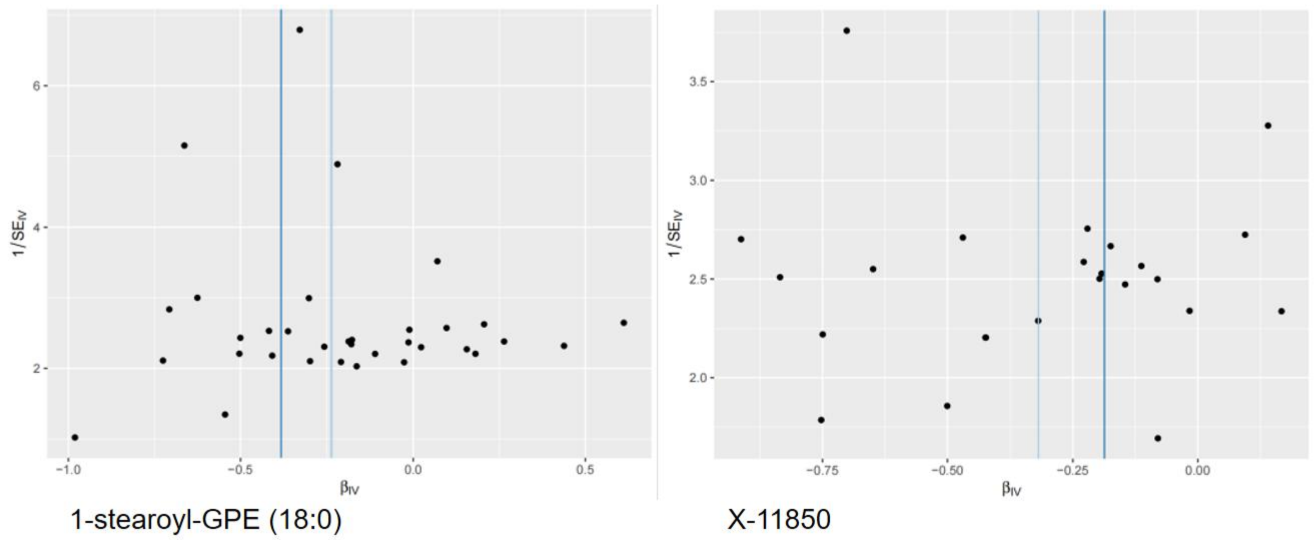

Funnel plots of MR analysis between metabolites and dry AMD

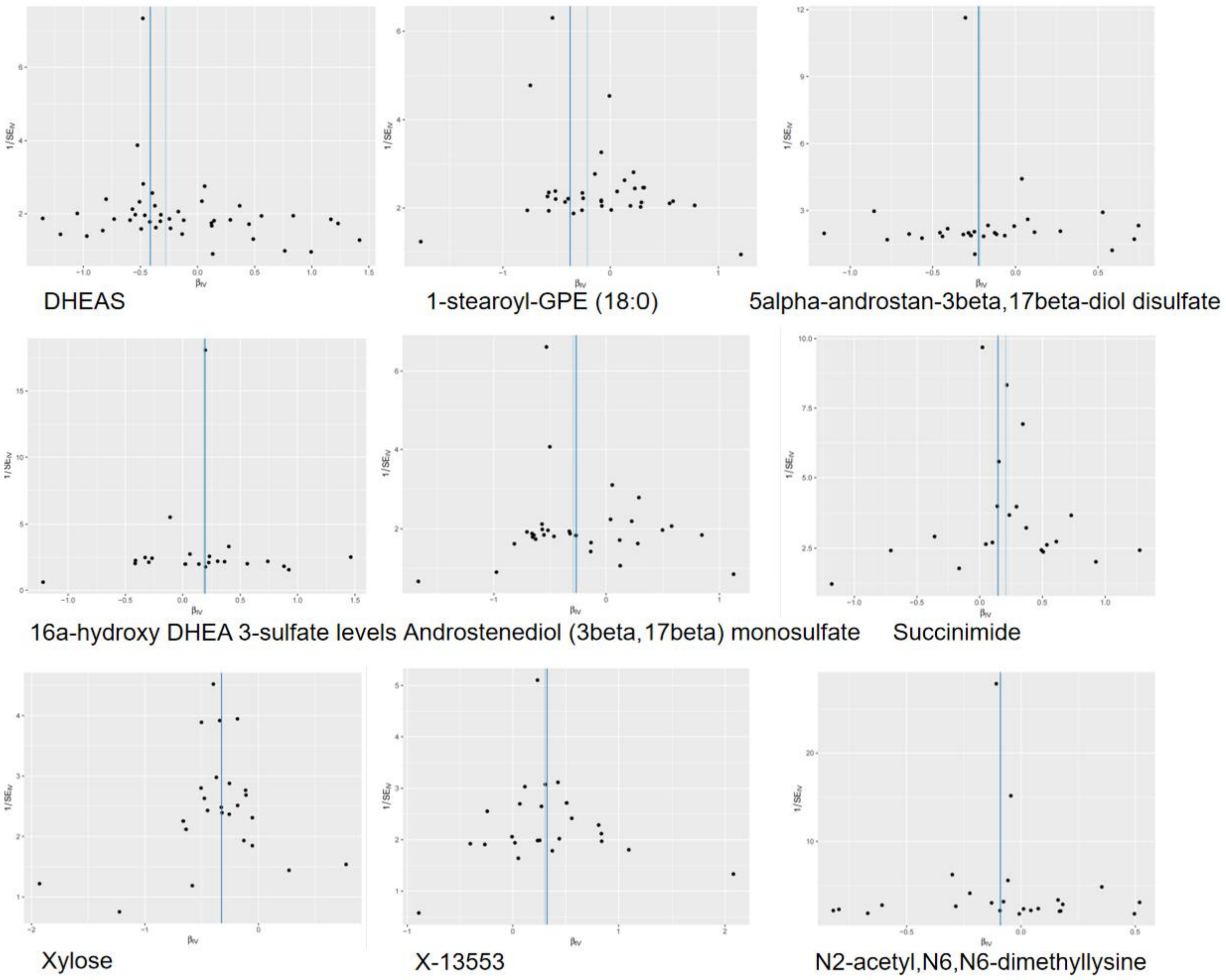

Funnel plots of MR analysis between metabolites and wet AMD
