# Supplementary material for: Causal association between metabolites and age-related macular degeneration: a bidirectional two-sample mendelian randomization study
Source: Hereditas. 2024 Dec 20;161:51. doi: 10.1186/s41065-024-00356-6 (PMC11662531; doi:10.1186/s41065-024-00356-6)
Supplement: Supplementary file 4 — Supplementary Material 4 [file 41065_2024_356_MOESM4_ESM.pdf]

Supplementary Table 1. GWAS datasets used for AMD in our study.

Phenotype	N (case / control)	Number of SNPs	Population	GWAS ID
Age-related macular degeneration (whether dry or wet)	209,122 (3,763 / 205,359)	16,380,424	European	finn-b-H7_AMD
Dry age-related macular degeneration (includes geographic atrophy)	208,690 (2,469 / 206,221)	16,380,423	European	finn-b-DRY_AMD
Wet age-related macular degeneration	208,715 (2,114 / 206,601)	16,380,422	European	finn-b-WET_AMD

GWAS: genome-wide association studies ; AMD: age-related macular degeneration; SNP: single nucleotide polymorphism.
