# Supplementary material for: Causal association between metabolites and age-related macular degeneration: a bidirectional two-sample mendelian randomization study
Source: Hereditas. 2024 Dec 20;161:51. doi: 10.1186/s41065-024-00356-6 (PMC11662531; doi:10.1186/s41065-024-00356-6)
Supplement: Supplementary file 5 — Supplementary Material 5 [file 41065_2024_356_MOESM5_ESM.pdf]

Supplementary Table 2. Confounding SNPs removed before final MR analysis.

| SNP | Confounding factors |
|---|---|
| rs139097404 | HDL cholesterol |
| rs10162642 | HDL cholesterol levels |
| rs1077834 | High density lipoprotein cholesterol levels |
| rs1260326 | High density lipoprotein cholesterol levels |
| rs12775431 | Age of smoking initiation |
| rs139097404 | HDL cholesterol |
| rs1532085 | High density lipoprotein cholesterol levels |
| rs174541 | Type 2 diabetes |
| rs174566 | High-density lipoprotein levels |
| rs2070895 | High density lipoprotein cholesterol levels |
| rs2342307 | Body mass index |
| rs2414577 | Age-related macular degeneration or COVID-19 critical illness |
| rs261291 | High density lipoprotein cholesterol levels |
| rs438811 | High density lipoprotein cholesterol levels |
| rs439401 | HDL cholesterol levels |
| rs4686471 | Type 2 diabetes |
| rs483082 | High density lipoprotein cholesterol levels |
| rs6078 | Cholesterol levels in large HDL |
| rs673548 | High density lipoprotein cholesterol levels |
| rs780093 | Type 2 diabetes |

MR: Mendelian randomization; SNP: single nucleotide polymorphisms; HDL: high density lipoprotein. COVID: corona virus disease.
