# Supplementary material for: Causal association between metabolites and age-related macular degeneration: a bidirectional two-sample mendelian randomization study
Source: Hereditas. 2024 Dec 20;161:51. doi: 10.1186/s41065-024-00356-6 (PMC11662531; doi:10.1186/s41065-024-00356-6)
Supplement: Supplementary file 6 — Supplementary Material 6 [file 41065_2024_356_MOESM6_ESM.pdf]

Supplementary Table 3. SNPs for final MR analysis.

SNP	effect_allele	other_allele	beta.exposure	beta.outcome	eaf.exposure	eaf.outcome	se.outcome	pval.outcome	outcome	proxy_snp	exposure	se.exposure	pval.exposure
rs10035725	G	T	0.07	0.04	0.5	0.46	0.03	1.01E-01	Age-related macular degeneration	NA	Gulonate levels	0.01	5.18E-06
rs10496399	C	T	-0.08	-0.01	0.22	0.3	0.03	6.63E-01	Age-related macular degeneration	NA	Gulonate levels	0.02	4.46E-06
rs10786430	C	A	0.13	-0.01	0.93	0.95	0.06	9.22E-01	Age-related macular degeneration	NA	Gulonate levels	0.03	9.74E-06
rs141569914	C	A	-0.12	0.12	0.09	0.07	0.05	1.51E-02	Age-related macular degeneration	NA	Gulonate levels	0.03	4.85E-06
rs149807601	T	C	-0.22	-0.15	0.02	0.03	0.08	7.12E-02	Age-related macular degeneration	NA	Gulonate levels	0.05	9.37E-06
rs187882577	A	G	-0.25	-0.12	0.02	0.01	0.11	2.65E-01	Age-related macular degeneration	NA	Gulonate levels	0.05	5.84E-08
rs2776037	C	T	0.09	0.01	0.58	0.56	0.03	7.96E-01	Age-related macular degeneration	NA	Gulonate levels	0.01	4.16E-09
rs34366619	T	C	0.39	0.19	0.01	0.01	0.12	1.20E-01	Age-related macular degeneration	NA	Gulonate levels	0.08	4.18E-06
rs3879823	C	T	0.17	0.06	0.05	0.05	0.06	2.76E-01	Age-related macular degeneration	NA	Gulonate levels	0.03	6.90E-07
rs3944510	A	C	-0.07	-0.02	0.61	0.59	0.03	5.13E-01	Age-related macular degeneration	NA	Gulonate levels	0.02	2.72E-06
rs3957147	T	C	0.09	0.06	0.17	0.23	0.03	5.64E-02	Age-related macular degeneration	NA	Gulonate levels	0.02	1.51E-06
rs4724828	T	C	0.1	0.07	0.89	0.83	0.03	2.77E-02	Age-related macular degeneration	NA	Gulonate levels	0.02	6.19E-06
rs6089583	A	G	-0.07	-0.01	0.67	0.65	0.03	7.70E-01	Age-related macular degeneration	NA	Gulonate levels	0.02	5.63E-06
rs61955537	A	T	-0.11	-0.02	0.2	0.15	0.03	5.57E-01	Age-related macular degeneration	rs9315666	Gulonate levels	0.02	3.22E-09

rs72815194	C	T	0.36	0.04	0.01	0.01	0.13	7.64E-01	Age-related macular degeneration	NA	Gulonate levels	0.08	5.99E-06
rs75072359	T	A	-0.19	-0.01	0.04	0.03	0.07	9.34E-01	Age-related macular degeneration	NA	Gulonate levels	0.04	2.39E-06
rs75759978	G	A	-0.21	0	0.03	0.01	0.14	9.73E-01	Age-related macular degeneration	NA	Gulonate levels	0.05	8.99E-06
rs77518118	T	A	0.32	-0.02	0.01	0.01	0.16	8.94E-01	Age-related macular degeneration	NA	Gulonate levels	0.07	1.77E-06
rs79253762	T	C	-0.18	0.03	0.04	0.03	0.07	6.35E-01	Age-related macular degeneration	NA	Gulonate levels	0.04	1.42E-06
rs79318581	A	G	0.17	0.09	0.04	0.02	0.08	2.63E-01	Age-related macular degeneration	NA	Gulonate levels	0.04	4.67E-06
rs9909758	C	G	0.12	0.06	0.1	0.15	0.04	1.06E-01	Age-related macular degeneration	NA	Gulonate levels	0.03	5.52E-06
rs10051746	C	T	-0.08	0.02	0.36	0.35	0.03	5.52E-01	Age-related macular degeneration	NA	Xylose levels	0.02	3.18E-06
rs10950862	G	T	-0.08	0.04	0.35	0.4	0.03	1.65E-01	Age-related macular degeneration	NA	Xylose levels	0.02	4.33E-06
rs112249340	A	G	0.3	-0.02	0.02	0	0.3	9.58E-01	Age-related macular degeneration	NA	Xylose levels	0.07	9.24E-06
rs113442137	A	G	0.17	-0.05	0.06	0.08	0.05	3.15E-01	Age-related macular degeneration	NA	Xylose levels	0.04	6.10E-06
rs115522284	A	G	-0.19	0.01	0.04	0.05	0.06	8.54E-01	Age-related macular degeneration	NA	Xylose levels	0.04	6.30E-06
rs12429666	A	G	0.34	-0.09	0.02	0.02	0.09	2.94E-01	Age-related macular degeneration	NA	Xylose levels	0.06	5.33E-08
rs12926601	T	C	0.1	-0.01	0.21	0.14	0.04	6.99E-01	Age-related macular degeneration	NA	Xylose levels	0.02	2.04E-06
rs12979487	G	A	0.3	-0.01	0.02	0.01	0.13	9.24E-01	Age-related macular degeneration	NA	Xylose levels	0.07	3.59E-06
rs13213275	T	C	0.08	-0.03	0.47	0.52	0.03	3.18E-01	Age-related macular degeneration	NA	Xylose levels	0.02	5.68E-06
rs138019197	A	G	0.55	-0.2	0	0.01	0.11	6.56E-02	Age-related macular	NA	Xylose levels	0.12	8.29E-06

									degeneration				
rs145187377	T	C	0.15	-0.04	0.07	0.09	0.04	3.89E-01	Age-related macular degeneration	NA	Xylose levels	0.03	5.23E-06
rs178730	T	A	-0.09	0.01	0.24	0.22	0.03	6.37E-01	Age-related macular degeneration	NA	Xylose levels	0.02	6.22E-06
rs1789555	G	A	0.24	0.1	0.02	0.01	0.13	4.24E-01	Age-related macular degeneration	NA	Xylose levels	0.05	9.93E-06
rs180932555	T	A	0.27	0.01	0.02	0.01	0.13	9.32E-01	Age-related macular degeneration	rs79137056	Xylose levels	0.06	7.75E-06
rs1911963	G	A	0.11	-0.01	0.8	0.74	0.03	7.96E-01	Age-related macular degeneration	NA	Xylose levels	0.02	2.68E-07
rs224946	A	G	-0.18	0.06	0.94	0.95	0.06	2.97E-01	Age-related macular degeneration	NA	Xylose levels	0.04	3.15E-06
rs2291265	T	C	0.11	-0.03	0.88	0.92	0.05	4.82E-01	Age-related macular degeneration	NA	Xylose levels	0.03	7.60E-06
rs533213061	G	A	-0.28	0.07	0.02	0.05	0.06	2.02E-01	Age-related macular degeneration	NA	Xylose levels	0.06	8.03E-06
rs56367544	C	T	0.3	-0.41	0.02	0	0.19	3.37E-02	Age-related macular degeneration	NA	Xylose levels	0.07	8.03E-06
rs62331361	T	C	0.31	-0.02	0.02	0.02	0.08	8.31E-01	Age-related macular degeneration	NA	Xylose levels	0.07	5.95E-06
rs62441094	A	G	-0.24	0.31	0.03	0.01	0.15	3.54E-02	Age-related macular degeneration	NA	Xylose levels	0.05	6.71E-06
rs7149187	A	G	0.09	0	0.29	0.29	0.03	9.99E-01	Age-related macular degeneration	NA	Xylose levels	0.02	5.65E-06
rs72817256	C	G	0.17	-0.03	0.05	0.06	0.05	5.64E-01	Age-related macular degeneration	NA	Xylose levels	0.04	5.78E-06
rs79930322	A	G	0.4	-0.06	0.01	0.03	0.08	4.08E-01	Age-related macular degeneration	NA	Xylose levels	0.08	5.46E-07
rs80310084	C	A	0.3	-0.07	0.02	0.07	0.05	1.56E-01	Age-related macular degeneration	NA	Xylose levels	0.06	1.23E-07
rs10146121	C	A	-0.07	-0.03	0.68	0.62	0.03	1.98E-01	Age-related macular degeneration	rs8018830	1-stearoyl-GPE (18:0) levels	0.02	9.21E-06

rs10234749	G	T	0.08	0.03	0.77	0.67	0.03	3.29E-01	Age-related macular degeneration	NA	1-stearoyl-GPE (18:0) levels	0.02	3.70E-06
rs11063303	T	G	-0.23	0.06	0.02	0.02	0.09	5.12E-01	Age-related macular degeneration	NA	1-stearoyl-GPE (18:0) levels	0.05	7.64E-06
rs117109614	C	T	-0.3	0.38	0.01	0	0.18	4.18E-02	Age-related macular degeneration	NA	1-stearoyl-GPE (18:0) levels	0.06	2.62E-06
rs117940165	G	T	0.28	0.04	0.02	0.04	0.07	5.66E-01	Age-related macular degeneration	NA	1-stearoyl-GPE (18:0) levels	0.06	4.47E-06
rs12292477	T	C	0.11	0.03	0.11	0.11	0.04	4.60E-01	Age-related macular degeneration	NA	1-stearoyl-GPE (18:0) levels	0.02	5.94E-06
rs12658974	A	C	0.11	-0.02	0.12	0.14	0.04	5.13E-01	Age-related macular degeneration	NA	1-stearoyl-GPE (18:0) levels	0.02	2.16E-06
rs12904030	T	C	-0.07	0.01	0.51	0.42	0.03	7.32E-01	Age-related macular degeneration	NA	1-stearoyl-GPE (18:0) levels	0.02	8.83E-06
rs13130041	T	C	0.08	-0.02	0.27	0.22	0.03	5.60E-01	Age-related macular degeneration	NA	1-stearoyl-GPE (18:0) levels	0.02	9.55E-06
rs1322199	A	G	0.08	-0.02	0.7	0.79	0.03	4.20E-01	Age-related macular degeneration	NA	1-stearoyl-GPE (18:0) levels	0.02	6.41E-06
rs1323678	A	C	-0.07	0	0.49	0.45	0.03	9.26E-01	Age-related macular degeneration	NA	1-stearoyl-GPE (18:0) levels	0.02	9.59E-06
rs13263105	G	C	-0.07	0.02	0.66	0.62	0.03	3.52E-01	Age-related macular degeneration	NA	1-stearoyl-GPE (18:0) levels	0.02	3.82E-06
rs135922	A	G	0.07	-0.05	0.49	0.48	0.03	3.94E-02	Age-related macular degeneration	NA	1-stearoyl-GPE (18:0) levels	0.02	1.56E-06
rs141999807	G	A	0.25	-0.05	0.02	0.03	0.07	4.47E-01	Age-related macular degeneration	NA	1-stearoyl-GPE (18:0) levels	0.05	2.14E-06
rs142999532	C	G	0.25	0.09	0.02	0	0.2	6.53E-01	Age-related macular degeneration	NA	1-stearoyl-GPE (18:0) levels	0.06	8.27E-06
rs144767726	A	C	-0.2	-0.11	0.03	0.03	0.08	1.65E-01	Age-related macular degeneration	NA	1-stearoyl-GPE (18:0) levels	0.04	5.71E-06
rs16979884	A	G	0.11	-0.04	0.1	0.09	0.05	3.50E-01	Age-related macular degeneration	NA	1-stearoyl-GPE (18:0) levels	0.03	7.81E-06
rs174560	C	T	-0.09	0.02	0.31	0.39	0.03	3.89E-01	Age-related macular	NA	1-stearoyl-GPE	0.02	1.52E-08

									degeneration		(18:0) levels		
rs17665744	G	T	0.1	-0.01	0.16	0.16	0.03	7.67E-01	Age-related macular degeneration	NA	1-stearoyl-GPE (18:0) levels	0.02	1.55E-06
rs1858942	A	G	0.07	-0.01	0.42	0.39	0.03	6.88E-01	Age-related macular degeneration	NA	1-stearoyl-GPE (18:0) levels	0.02	2.23E-06
rs1866637	T	C	0.08	-0.02	0.23	0.2	0.03	4.46E-01	Age-related macular degeneration	NA	1-stearoyl-GPE (18:0) levels	0.02	4.14E-06
rs2004196	T	C	0.1	-0.01	0.14	0.22	0.03	6.57E-01	Age-related macular degeneration	NA	1-stearoyl-GPE (18:0) levels	0.02	1.33E-06
rs2540682	A	T	0.09	-0.03	0.18	0.16	0.03	3.62E-01	Age-related macular degeneration	NA	1-stearoyl-GPE (18:0) levels	0.02	9.59E-06
rs2935412	T	C	-0.1	0.02	0.85	0.85	0.03	5.33E-01	Age-related macular degeneration	NA	1-stearoyl-GPE (18:0) levels	0.02	9.68E-06
rs35853021	T	G	0.21	-0.09	0.35	0.4	0.03	4.44E-04	Age-related macular degeneration	NA	1-stearoyl-GPE (18:0) levels	0.02	1.38E-40
rs3779773	C	G	0.1	0.01	0.16	0.14	0.04	8.43E-01	Age-related macular degeneration	NA	1-stearoyl-GPE (18:0) levels	0.02	1.79E-06
rs526018	G	A	-0.08	-0.04	0.28	0.35	0.03	1.09E-01	Age-related macular degeneration	NA	1-stearoyl-GPE (18:0) levels	0.02	8.99E-07
rs62036205	C	A	-0.13	0.06	0.09	0.1	0.04	1.38E-01	Age-related macular degeneration	NA	1-stearoyl-GPE (18:0) levels	0.03	9.36E-07
rs633695	G	A	0.17	-0.11	0.28	0.31	0.03	3.24E-05	Age-related macular degeneration	NA	1-stearoyl-GPE (18:0) levels	0.02	1.41E-23
rs6999569	G	A	-0.08	0	0.48	0.46	0.03	8.43E-01	Age-related macular degeneration	NA	1-stearoyl-GPE (18:0) levels	0.02	2.71E-07
rs7488822	G	A	0.4	0.04	0.01	0.01	0.14	7.68E-01	Age-related macular degeneration	NA	1-stearoyl-GPE (18:0) levels	0.09	3.81E-06
rs77692382	T	C	0.18	-0.03	0.25	0.2	0.03	2.92E-01	Age-related macular degeneration	NA	1-stearoyl-GPE (18:0) levels	0.02	5.77E-26
rs79696139	A	G	0.19	0.01	0.04	0.04	0.06	8.99E-01	Age-related macular degeneration	NA	1-stearoyl-GPE (18:0) levels	0.04	2.27E-06
rs8058610	T	C	0.07	-0.01	0.6	0.55	0.03	7.82E-01	Age-related macular degeneration	NA	1-stearoyl-GPE (18:0) levels	0.02	3.39E-06

rs10842320	T	G	-0.09	0.02	0.26	0.29	0.03	5.53E-01	Age-related macular degeneration	NA	X-11850 levels	0.02	1.25E-06
rs10963516	A	G	0.09	-0.05	0.2	0.16	0.03	1.53E-01	Age-related macular degeneration	NA	X-11850 levels	0.02	3.02E-06
rs113125510	A	G	-0.19	0.01	0.04	0.05	0.06	8.43E-01	Age-related macular degeneration	NA	X-11850 levels	0.04	1.05E-06
rs11597566	G	A	0.23	-0.03	0.03	0.01	0.11	7.82E-01	Age-related macular degeneration	NA	X-11850 levels	0.05	7.28E-06
rs117950536	C	A	-0.29	0.12	0.02	0.02	0.1	2.07E-01	Age-related macular degeneration	NA	X-11850 levels	0.06	2.78E-06
rs1205283	G	T	-0.09	0.05	0.78	0.76	0.03	1.18E-01	Age-related macular degeneration	NA	X-11850 levels	0.02	2.99E-06
rs12433497	T	C	0.09	0	0.21	0.28	0.03	9.04E-01	Age-related macular degeneration	NA	X-11850 levels	0.02	9.91E-06
rs138886343	A	G	-0.2	0.08	0.04	0.09	0.05	9.55E-02	Age-related macular degeneration	NA	X-11850 levels	0.04	6.21E-07
rs142840963	C	T	0.21	-0.05	0.03	0.03	0.07	4.74E-01	Age-related macular degeneration	NA	X-11850 levels	0.05	6.88E-06
rs17348202	C	T	0.15	-0.03	0.05	0.03	0.07	6.67E-01	Age-related macular degeneration	NA	X-11850 levels	0.03	9.20E-06
rs181782952	C	A	-0.32	0.03	0.01	0.03	0.08	7.30E-01	Age-related macular degeneration	NA	X-11850 levels	0.07	3.05E-06
rs3884707	T	G	0.08	-0.01	0.26	0.3	0.03	7.12E-01	Age-related macular degeneration	NA	X-11850 levels	0.02	4.54E-06
rs4576774	C	A	0.07	-0.03	0.35	0.28	0.03	2.20E-01	Age-related macular degeneration	NA	X-11850 levels	0.02	6.32E-06
rs4831602	T	C	-0.13	0.01	0.92	0.93	0.05	8.15E-01	Age-related macular degeneration	NA	X-11850 levels	0.03	7.61E-06
rs4858791	T	C	0.2	-0.11	0.97	0.96	0.07	8.94E-02	Age-related macular degeneration	NA	X-11850 levels	0.04	3.20E-06
rs58280688	C	G	-0.22	-0.03	0.03	0.03	0.08	7.40E-01	Age-related macular degeneration	NA	X-11850 levels	0.05	7.49E-06
rs62512054	C	T	-0.33	0.17	0.01	0.01	0.15	2.58E-01	Age-related macular	NA	X-11850 levels	0.07	5.28E-06

									degeneration					
rs72676435	T	C	-0.17	-0.03	0.05	0.05	0.06	6.79E-01	Age-related degeneration	macular	NA	X-11850 levels	0.04	5.19E-06
rs75195552	A	G	-0.09	0	0.24	0.26	0.03	9.31E-01	Age-related degeneration	macular	NA	X-11850 levels	0.02	1.40E-06
rs76033483	T	C	0.21	0.01	0.03	0.04	0.07	8.76E-01	Age-related degeneration	macular	NA	X-11850 levels	0.05	6.65E-06
rs7664690	C	T	-0.09	0.06	0.17	0.26	0.03	2.31E-02	Age-related degeneration	macular	NA	X-11850 levels	0.02	7.04E-06
rs76917355	A	G	0.14	0.03	0.07	0.1	0.04	5.35E-01	Age-related degeneration	macular	NA	X-11850 levels	0.03	3.44E-06
rs79449392	A	G	0.32	-0.1	0.01	0.02	0.1	3.07E-01	Age-related degeneration	macular	NA	X-11850 levels	0.07	3.75E-06
rs10870254	G	T	-0.28	-0.02	0.01	0.01	0.11	8.55E-01	Age-related degeneration	macular	NA	Mannonate levels	0.06	8.67E-06
rs113950720	G	A	0.19	0.03	0.03	0.04	0.07	6.31E-01	Age-related degeneration	macular	NA	Mannonate levels	0.04	6.56E-06
rs11577971	T	C	-0.16	0.03	0.05	0.04	0.07	6.93E-01	Age-related degeneration	macular	NA	Mannonate levels	0.03	5.18E-06
rs118178329	A	G	0.31	0.01	0.01	0.03	0.07	8.95E-01	Age-related degeneration	macular	NA	Mannonate levels	0.07	7.79E-06
rs12141660	T	C	-0.08	-0.01	0.31	0.35	0.03	6.44E-01	Age-related degeneration	macular	NA	Mannonate levels	0.02	1.66E-07
rs12658815	T	G	1.8	0.21	0	0.04	0.06	1.12E-03	Age-related degeneration	macular	NA	Mannonate levels	0.33	5.07E-08
rs138718435	A	C	0.32	0.1	0.01	0.02	0.08	2.20E-01	Age-related degeneration	macular	NA	Mannonate levels	0.07	7.42E-06
rs148622151	C	G	-0.23	-0.06	0.02	0.01	0.12	6.07E-01	Age-related degeneration	macular	NA	Mannonate levels	0.05	5.23E-06
rs1921903	T	C	-0.07	0.03	0.52	0.64	0.03	2.13E-01	Age-related degeneration	macular	NA	Mannonate levels	0.01	8.94E-06
rs34117505	T	C	0.07	-0.02	0.47	0.36	0.03	4.90E-01	Age-related degeneration	macular	NA	Mannonate levels	0.01	4.77E-06

rs34945403	G	A	-0.15	-0.04	0.06	0.04	0.06	5.24E-01	Age-related macular degeneration	NA	Mannonate levels	0.03	1.88E-06
rs35646619	G	A	-0.07	0.03	0.35	0.37	0.03	2.75E-01	Age-related macular degeneration	NA	Mannonate levels	0.02	2.68E-06
rs3808945	T	C	-0.07	0	0.34	0.34	0.03	8.90E-01	Age-related macular degeneration	NA	Mannonate levels	0.02	2.40E-06
rs544271348	G	T	0.17	0.03	0.04	0.02	0.09	7.55E-01	Age-related macular degeneration	NA	Mannonate levels	0.04	5.09E-06
rs73072354	G	A	0.3	0.23	0.01	0.02	0.1	2.36E-02	Age-related macular degeneration	NA	Mannonate levels	0.07	8.49E-06
rs74959984	C	T	-0.18	0.13	0.04	0.06	0.06	2.07E-02	Age-related macular degeneration	NA	Mannonate levels	0.04	7.37E-06
rs76645474	C	G	-0.29	-0.12	0.01	0.01	0.15	4.35E-01	Age-related macular degeneration	NA	Mannonate levels	0.06	3.38E-06
rs77587732	T	C	0.17	0.02	0.04	0.05	0.06	6.72E-01	Age-related macular degeneration	NA	Mannonate levels	0.04	1.45E-06
rs78802404	A	T	0.29	0.41	0.01	0	0.36	2.44E-01	Age-related macular degeneration	NA	Mannonate levels	0.07	8.73E-06
rs80178342	C	T	-0.17	0	0.04	0.05	0.06	9.77E-01	Age-related macular degeneration	NA	Mannonate levels	0.04	2.63E-06
rs11122398	C	T	0.19	0.01	0.03	0.02	0.08	8.99E-01	Age-related macular degeneration	NA	Stearoyl sphingomyelin (d18:1/18:0) levels	0.04	6.68E-06
rs113807400	C	T	0.14	-0.02	0.07	0.11	0.04	5.40E-01	Age-related macular degeneration	NA	Stearoyl sphingomyelin (d18:1/18:0) levels	0.03	4.10E-06
rs116987964	C	T	-0.27	0.03	0.02	0	0.19	8.69E-01	Age-related macular degeneration	NA	Stearoyl sphingomyelin (d18:1/18:0) levels	0.06	4.92E-06
rs117435952	A	G	-0.15	0.04	0.06	0.04	0.06	5.71E-01	Age-related macular	NA	Stearoyl	0.03	7.88E-06

									degeneration			sphingomyelin (d18:1/18:0) levels		
rs118118765	G	A	-0.31	-0.01	0.01	0.03	0.07	9.15E-01	Age-related degeneration	macular	NA	Stearoyl sphingomyelin (d18:1/18:0) levels	0.06	1.77E-06
rs11930021	A	C	-0.07	-0.02	0.5	0.59	0.03	4.49E-01	Age-related degeneration	macular	rs1002 9190	Stearoyl sphingomyelin (d18:1/18:0) levels	0.02	8.88E-06
rs11989411	A	G	0.09	-0.07	0.74	0.73	0.03	1.26E-02	Age-related degeneration	macular	NA	Stearoyl sphingomyelin (d18:1/18:0) levels	0.02	4.40E-07
rs13419910	G	A	-0.07	-0.02	0.38	0.33	0.03	5.25E-01	Age-related degeneration	macular	NA	Stearoyl sphingomyelin (d18:1/18:0) levels	0.02	9.44E-06
rs139889411	A	G	-0.3	-0.08	0.01	0.02	0.09	3.47E-01	Age-related degeneration	macular	NA	Stearoyl sphingomyelin (d18:1/18:0) levels	0.07	8.12E-06
rs141838588	C	T	-0.17	-0.01	0.07	0.05	0.06	8.24E-01	Age-related degeneration	macular	NA	Stearoyl sphingomyelin (d18:1/18:0) levels	0.03	4.48E-09
rs144528624	T	C	-0.26	0.1	0.02	0.02	0.1	2.98E-01	Age-related degeneration	macular	NA	Stearoyl sphingomyelin (d18:1/18:0) levels	0.05	1.66E-07
rs146159084	A	G	-0.35	0.09	0.01	0.03	0.07	2.33E-01	Age-related degeneration	macular	NA	Stearoyl sphingomyelin	0.07	2.97E-06

												(d18:1/18:0) levels		
rs1466448	A	C	0.23	-0.09	0.81	0.74	0.03	2.00E-03	Age-related degeneration	macular	NA	Stearoyl sphingomyelin (d18:1/18:0) levels	0.02	1.95E-34
rs16846237	A	G	0.41	0.04	0.01	0.02	0.1	6.96E-01	Age-related degeneration	macular	NA	Stearoyl sphingomyelin (d18:1/18:0) levels	0.09	6.55E-06
rs168622	G	T	-0.09	0.01	0.61	0.66	0.03	7.06E-01	Age-related degeneration	macular	NA	Stearoyl sphingomyelin (d18:1/18:0) levels	0.02	2.09E-09
rs17101394	A	G	0.18	-0.04	0.16	0.13	0.04	2.32E-01	Age-related degeneration	macular	NA	Stearoyl sphingomyelin (d18:1/18:0) levels	0.02	4.47E-19
rs17114133	T	C	-0.24	0.12	0.02	0.03	0.07	8.15E-02	Age-related degeneration	macular	NA	Stearoyl sphingomyelin (d18:1/18:0) levels	0.05	1.17E-06
rs17123032	A	G	-0.19	-0.09	0.03	0.02	0.09	2.98E-01	Age-related degeneration	macular	NA	Stearoyl sphingomyelin (d18:1/18:0) levels	0.04	8.54E-06
rs174604	G	C	-0.1	0.02	0.37	0.38	0.03	4.60E-01	Age-related degeneration	macular	NA	Stearoyl sphingomyelin (d18:1/18:0) levels	0.02	9.68E-10
rs188389015	C	G	-0.41	0.24	0.01	0	0.2	2.43E-01	Age-related degeneration	macular	NA	Stearoyl sphingomyelin (d18:1/18:0)	0.09	2.91E-06

												levels		
rs2297983	T	G	0.07	-0.01	0.53	0.58	0.03	5.88E-01	Age-related degeneration	macular	NA	Stearoyl sphingomyelin (d18:1/18:0) levels	0.01	9.25E-06
rs2794324	C	G	-0.23	-0.08	0.02	0.01	0.12	4.95E-01	Age-related degeneration	macular	NA	Stearoyl sphingomyelin (d18:1/18:0) levels	0.05	4.02E-06
rs35193317	T	C	0.07	0	0.49	0.47	0.03	9.85E-01	Age-related degeneration	macular	NA	Stearoyl sphingomyelin (d18:1/18:0) levels	0.02	4.04E-06
rs3895559	A	G	0.07	-0.05	0.46	0.44	0.03	7.27E-02	Age-related degeneration	macular	NA	Stearoyl sphingomyelin (d18:1/18:0) levels	0.02	2.23E-06
rs445925	A	G	-0.15	0.07	0.11	0.07	0.05	1.14E-01	Age-related degeneration	macular	NA	Stearoyl sphingomyelin (d18:1/18:0) levels	0.02	1.68E-09
rs4787181	G	C	-0.11	-0.03	0.1	0.13	0.04	3.93E-01	Age-related degeneration	macular	NA	Stearoyl sphingomyelin (d18:1/18:0) levels	0.03	9.35E-06
rs55730499	T	C	0.16	-0.08	0.07	0.05	0.06	1.98E-01	Age-related degeneration	macular	NA	Stearoyl sphingomyelin (d18:1/18:0) levels	0.03	6.42E-08
rs7135337	C	A	0.08	-0.01	0.59	0.52	0.03	7.30E-01	Age-related degeneration	macular	NA	Stearoyl sphingomyelin (d18:1/18:0) levels	0.02	1.42E-07

rs7260306	T	G	-0.07	-0.01	0.4	0.41	0.03	6.95E-01	Age-related macular degeneration	NA	Stearoyl sphingomyelin (d18:1/18:0) levels	0.02	5.15E-06
rs75431233	A	G	-0.23	-0.08	0.03	0.02	0.09	3.43E-01	Age-related macular degeneration	NA	Stearoyl sphingomyelin (d18:1/18:0) levels	0.05	1.08E-06
rs76300790	T	A	0.1	-0.03	0.17	0.16	0.03	3.49E-01	Age-related macular degeneration	NA	Stearoyl sphingomyelin (d18:1/18:0) levels	0.02	9.47E-07
rs76572147	T	C	-0.48	0.25	0.01	0.01	0.16	1.15E-01	Age-related macular degeneration	NA	Stearoyl sphingomyelin (d18:1/18:0) levels	0.09	8.73E-08
rs7871183	T	C	-0.32	-0.08	0.01	0.02	0.09	3.34E-01	Age-related macular degeneration	rs73661789	Stearoyl sphingomyelin (d18:1/18:0) levels	0.07	1.27E-06
rs78831353	C	T	-0.25	0.04	0.02	0.04	0.07	5.65E-01	Age-related macular degeneration	NA	Stearoyl sphingomyelin (d18:1/18:0) levels	0.05	2.94E-06
rs9846165	C	T	-0.09	0.03	0.74	0.58	0.03	1.77E-01	Age-related macular degeneration	NA	Stearoyl sphingomyelin (d18:1/18:0) levels	0.02	6.82E-08
rs11174150	T	C	0.07	-0.03	0.34	0.29	0.03	2.58E-01	Age-related macular degeneration	NA	Androstenediol (3beta,17beta) monosulfate (1) levels	0.01	5.64E-06
rs111936578	C	T	-0.22	0.25	0.02	0	0.18	1.65E-01	Age-related macular	NA	Androstenediol	0.05	7.46E-06

									degeneration			(3beta,17beta) monosulfate (1) levels		
rs12038241	G	A	-0.06	0	0.56	0.64	0.03	8.94E-01	Age-related degeneration	macular	NA	Androstenediol (3beta,17beta) monosulfate (1) levels	0.01	5.88E-06
rs141370991	T	C	-0.23	-0.02	0.02	0.03	0.08	7.54E-01	Age-related degeneration	macular	NA	Androstenediol (3beta,17beta) monosulfate (1) levels	0.05	2.02E-06
rs142274824	T	C	-0.33	-0.12	0.01	0.01	0.12	3.10E-01	Age-related degeneration	macular	NA	Androstenediol (3beta,17beta) monosulfate (1) levels	0.07	9.75E-06
rs144814135	A	G	-0.25	0.05	0.02	0.01	0.11	6.27E-01	Age-related degeneration	macular	NA	Androstenediol (3beta,17beta) monosulfate (1) levels	0.05	3.13E-06
rs1467067	C	A	0.07	0	0.31	0.25	0.03	8.91E-01	Age-related degeneration	macular	NA	Androstenediol (3beta,17beta) monosulfate (1) levels	0.01	3.17E-06
rs146747578	C	G	-0.29	0.16	0.01	0.01	0.14	2.52E-01	Age-related degeneration	macular	NA	Androstenediol (3beta,17beta) monosulfate (1) levels	0.07	9.63E-06
rs147871557	G	A	0.32	-0.59	0.01	0	0.37	1.11E-01	Age-related degeneration	macular	NA	Androstenediol (3beta,17beta) monosulfate (1) levels	0.07	8.20E-06
rs148982377	C	T	-0.38	0.06	0.03	0.09	0.04	1.44E-01	Age-related degeneration	macular	NA	Androstenediol (3beta,17beta)	0.04	6.86E-25

											monosulfate (1) levels		
rs149982314	T	C	-0.32	0.02	0.01	0	0.29	9.42E-01	Age-related degeneration	macular	NA	Androstenediol (3beta,17beta) monosulfate (1) levels	0.07	5.64E-06
rs1871395	G	A	0.11	-0.01	0.16	0.28	0.03	6.70E-01	Age-related degeneration	macular	NA	Androstenediol (3beta,17beta) monosulfate (1) levels	0.02	1.18E-09
rs191866531	A	G	0.42	0.01	0.01	0.01	0.11	9.18E-01	Age-related degeneration	macular	NA	Androstenediol (3beta,17beta) monosulfate (1) levels	0.07	2.00E-08
rs28524991	A	T	-0.19	0.04	0.03	0.02	0.09	7.00E-01	Age-related degeneration	macular	NA	Androstenediol (3beta,17beta) monosulfate (1) levels	0.04	3.31E-06
rs55677910	G	A	-0.09	0.03	0.14	0.15	0.04	3.41E-01	Age-related degeneration	macular	NA	Androstenediol (3beta,17beta) monosulfate (1) levels	0.02	3.07E-06
rs55764900	G	A	-0.09	0.06	0.16	0.12	0.04	9.12E-02	Age-related degeneration	macular	NA	Androstenediol (3beta,17beta) monosulfate (1) levels	0.02	2.35E-06
rs677027	A	G	-0.06	0.05	0.49	0.49	0.03	4.72E-02	Age-related degeneration	macular	NA	Androstenediol (3beta,17beta) monosulfate (1) levels	0.01	5.61E-06
rs706795	C	T	0.06	-0.01	0.59	0.59	0.03	6.34E-01	Age-related degeneration	macular	NA	Androstenediol (3beta,17beta) monosulfate (1)	0.01	4.21E-06

												levels		
rs7086721	C	T	0.07	0	0.7	0.7	0.03	9.21E-01	Age-related degeneration	macular	NA	Androstenediol (3beta,17beta) monosulfate (1) levels	0.02	9.63E-06
rs7173332	C	G	-0.09	0.05	0.16	0.19	0.03	1.09E-01	Age-related degeneration	macular	NA	Androstenediol (3beta,17beta) monosulfate (1) levels	0.02	3.07E-06
rs72729813	C	G	-0.13	0.07	0.06	0.05	0.06	2.18E-01	Age-related degeneration	macular	NA	Androstenediol (3beta,17beta) monosulfate (1) levels	0.03	7.14E-06
rs75107956	C	A	0.16	0.02	0.04	0.06	0.05	7.28E-01	Age-related degeneration	macular	rs143367698	Androstenediol (3beta,17beta) monosulfate (1) levels	0.04	8.92E-06
rs75561083	T	C	0.26	-0.04	0.02	0.07	0.05	3.54E-01	Age-related degeneration	macular	NA	Androstenediol (3beta,17beta) monosulfate (1) levels	0.05	3.38E-06
rs76294479	T	C	0.14	0.04	0.07	0.04	0.07	5.32E-01	Age-related degeneration	macular	NA	Androstenediol (3beta,17beta) monosulfate (1) levels	0.03	7.79E-08
rs7675318	T	C	0.08	0	0.18	0.17	0.03	9.66E-01	Age-related degeneration	macular	NA	Androstenediol (3beta,17beta) monosulfate (1) levels	0.02	4.19E-06
rs7687817	T	C	-0.11	-0.02	0.09	0.05	0.06	7.01E-01	Age-related degeneration	macular	NA	Androstenediol (3beta,17beta) monosulfate (1) levels	0.02	5.33E-06

rs78438321	T	C	-0.27	-0.05	0.02	0	0.19	7.87E-01	Age-related macular degeneration	NA	Androstenediol (3beta,17beta) monosulfate (1) levels	0.05	5.05E-07
rs784420	G	A	0.07	-0.04	0.3	0.29	0.03	1.91E-01	Age-related macular degeneration	NA	Androstenediol (3beta,17beta) monosulfate (1) levels	0.01	8.71E-06
rs9407758	A	C	-0.07	0	0.43	0.42	0.03	9.61E-01	Age-related macular degeneration	NA	Androstenediol (3beta,17beta) monosulfate (1) levels	0.01	2.65E-06
rs9420589	T	G	-0.06	0.01	0.43	0.45	0.03	7.72E-01	Age-related macular degeneration	NA	Androstenediol (3beta,17beta) monosulfate (1) levels	0.01	3.31E-06
rs955783	T	A	0.07	-0.02	0.4	0.37	0.03	4.09E-01	Age-related macular degeneration	NA	Androstenediol (3beta,17beta) monosulfate (1) levels	0.01	2.32E-06
rs10146121	C	A	-0.07	-0.03	0.68	0.62	0.03	3.09E-01	Dry age-related macular degeneration (includes geographic atrophy)	rs8018830	1-stearoyl-GPE (18:0) levels	0.02	9.21E-06
rs10234749	G	T	0.08	0.02	0.77	0.67	0.03	5.88E-01	Dry age-related macular degeneration (includes geographic atrophy)	NA	1-stearoyl-GPE (18:0) levels	0.02	3.70E-06
rs11063303	T	G	-0.23	0.05	0.02	0.02	0.11	6.61E-01	Dry age-related macular degeneration (includes geographic atrophy)	NA	1-stearoyl-GPE (18:0) levels	0.05	7.64E-06
rs117109614	C	T	-0.3	0.16	0.01	0	0.22	4.63E-01	Dry age-related macular degeneration (includes geographic atrophy)	NA	1-stearoyl-GPE (18:0) levels	0.06	2.62E-06
rs117940165	G	T	0.28	0.02	0.02	0.04	0.08	8.05E-01	Dry age-related macular	NA	1-stearoyl-GPE	0.06	4.47E-06

									degeneration (includes geographic atrophy)		(18:0) levels		
rs12292477	T	C	0.11	0.02	0.11	0.11	0.05	7.25E-01	Dry age-related macular degeneration (includes geographic atrophy)	NA	1-stearoyl-GPE (18:0) levels	0.02	5.94E-06
rs12658974	A	C	0.11	-0.04	0.12	0.14	0.04	3.60E-01	Dry age-related macular degeneration (includes geographic atrophy)	NA	1-stearoyl-GPE (18:0) levels	0.02	2.16E-06
rs12904030	T	C	-0.07	0.03	0.51	0.42	0.03	2.67E-01	Dry age-related macular degeneration (includes geographic atrophy)	NA	1-stearoyl-GPE (18:0) levels	0.02	8.83E-06
rs13130041	T	C	0.08	0	0.27	0.22	0.04	9.55E-01	Dry age-related macular degeneration (includes geographic atrophy)	NA	1-stearoyl-GPE (18:0) levels	0.02	9.55E-06
rs1322199	A	G	0.08	-0.01	0.7	0.79	0.04	7.39E-01	Dry age-related macular degeneration (includes geographic atrophy)	NA	1-stearoyl-GPE (18:0) levels	0.02	6.41E-06
rs1323678	A	C	-0.07	0.01	0.49	0.45	0.03	8.06E-01	Dry age-related macular degeneration (includes geographic atrophy)	NA	1-stearoyl-GPE (18:0) levels	0.02	9.59E-06
rs13263105	G	C	-0.07	0.01	0.66	0.62	0.03	6.55E-01	Dry age-related macular degeneration (includes geographic atrophy)	NA	1-stearoyl-GPE (18:0) levels	0.02	3.82E-06
rs135922	A	G	0.07	-0.04	0.49	0.48	0.03	2.23E-01	Dry age-related macular degeneration (includes geographic atrophy)	NA	1-stearoyl-GPE (18:0) levels	0.02	1.56E-06
rs141999807	G	A	0.25	-0.16	0.02	0.03	0.08	6.04E-02	Dry age-related macular degeneration (includes geographic atrophy)	NA	1-stearoyl-GPE (18:0) levels	0.05	2.14E-06
rs142999532	C	G	0.25	-0.24	0.02	0	0.24	3.17E-01	Dry age-related macular degeneration (includes geographic atrophy)	NA	1-stearoyl-GPE (18:0) levels	0.06	8.27E-06
rs144767726	A	C	-0.2	-0.04	0.03	0.03	0.09	6.90E-01	Dry age-related macular	NA	1-stearoyl-GPE	0.04	5.71E-06

									degeneration (includes geographic atrophy)		(18:0) levels		
rs16979884	A	G	0.11	-0.03	0.1	0.09	0.05	5.31E-01	Dry age-related macular degeneration (includes geographic atrophy)	NA	1-stearoyl-GPE (18:0) levels	0.03	7.81E-06
rs174560	C	T	-0.09	0.03	0.31	0.39	0.03	3.67E-01	Dry age-related macular degeneration (includes geographic atrophy)	NA	1-stearoyl-GPE (18:0) levels	0.02	1.52E-08
rs17665744	G	T	0.1	-0.02	0.16	0.16	0.04	6.68E-01	Dry age-related macular degeneration (includes geographic atrophy)	NA	1-stearoyl-GPE (18:0) levels	0.02	1.55E-06
rs1858942	A	G	0.07	-0.01	0.42	0.39	0.03	6.73E-01	Dry age-related macular degeneration (includes geographic atrophy)	NA	1-stearoyl-GPE (18:0) levels	0.02	2.23E-06
rs1866637	T	C	0.08	-0.03	0.23	0.2	0.04	3.73E-01	Dry age-related macular degeneration (includes geographic atrophy)	NA	1-stearoyl-GPE (18:0) levels	0.02	4.14E-06
rs2004196	T	C	0.1	-0.07	0.14	0.22	0.04	4.51E-02	Dry age-related macular degeneration (includes geographic atrophy)	NA	1-stearoyl-GPE (18:0) levels	0.02	1.33E-06
rs2540682	A	T	0.09	-0.06	0.18	0.16	0.04	1.26E-01	Dry age-related macular degeneration (includes geographic atrophy)	NA	1-stearoyl-GPE (18:0) levels	0.02	9.59E-06
rs2935412	T	C	-0.1	0.02	0.85	0.85	0.04	5.54E-01	Dry age-related macular degeneration (includes geographic atrophy)	NA	1-stearoyl-GPE (18:0) levels	0.02	9.68E-06
rs35853021	T	G	0.21	-0.07	0.35	0.4	0.03	2.59E-02	Dry age-related macular degeneration (includes geographic atrophy)	NA	1-stearoyl-GPE (18:0) levels	0.02	1.38E-40
rs3779773	C	G	0.1	0	0.16	0.14	0.04	9.58E-01	Dry age-related macular degeneration (includes geographic atrophy)	NA	1-stearoyl-GPE (18:0) levels	0.02	1.79E-06
rs526018	G	A	-0.08	-0.05	0.28	0.35	0.03	1.05E-01	Dry age-related macular	NA	1-stearoyl-GPE	0.02	8.99E-07

									degeneration (includes geographic atrophy)		(18:0) levels		
rs62036205	C	A	-0.13	0.05	0.09	0.1	0.05	2.91E-01	Dry age-related macular degeneration (includes geographic atrophy)	NA	1-stearoyl-GPE (18:0) levels	0.03	9.36E-07
rs633695	G	A	0.17	-0.11	0.28	0.31	0.03	6.44E-04	Dry age-related macular degeneration (includes geographic atrophy)	NA	1-stearoyl-GPE (18:0) levels	0.02	1.41E-23
rs6999569	G	A	-0.08	-0.01	0.48	0.46	0.03	8.03E-01	Dry age-related macular degeneration (includes geographic atrophy)	NA	1-stearoyl-GPE (18:0) levels	0.02	2.71E-07
rs7488822	G	A	0.4	0.11	0.01	0.01	0.17	5.30E-01	Dry age-related macular degeneration (includes geographic atrophy)	NA	1-stearoyl-GPE (18:0) levels	0.09	3.81E-06
rs77692382	T	C	0.18	-0.04	0.25	0.2	0.04	2.83E-01	Dry age-related macular degeneration (includes geographic atrophy)	NA	1-stearoyl-GPE (18:0) levels	0.02	5.77E-26
rs79696139	A	G	0.19	0	0.04	0.04	0.07	9.77E-01	Dry age-related macular degeneration (includes geographic atrophy)	NA	1-stearoyl-GPE (18:0) levels	0.04	2.27E-06
rs8058610	T	C	0.07	0	0.6	0.55	0.03	9.75E-01	Dry age-related macular degeneration (includes geographic atrophy)	NA	1-stearoyl-GPE (18:0) levels	0.02	3.39E-06
rs10842320	T	G	-0.09	0.02	0.26	0.29	0.03	5.54E-01	Dry age-related macular degeneration (includes geographic atrophy)	NA	X-11850 levels	0.02	1.25E-06
rs10963516	A	G	0.09	-0.07	0.2	0.16	0.04	9.60E-02	Dry age-related macular degeneration (includes geographic atrophy)	NA	X-11850 levels	0.02	3.02E-06
rs113125510	A	G	-0.19	0.04	0.04	0.05	0.07	5.43E-01	Dry age-related macular degeneration (includes geographic atrophy)	NA	X-11850 levels	0.04	1.05E-06
rs11597566	G	A	0.23	-0.02	0.03	0.01	0.13	8.93E-01	Dry age-related macular	NA	X-11850 levels	0.05	7.28E-06

								degeneration (includes geographic atrophy)				
rs117950536	C	A	-0.29	0.06	0.02	0.02	0.12	6.23E-01	Dry age-related macular degeneration (includes geographic atrophy)	NA	X-11850 levels	0.06	2.78E-06
rs1205283	G	T	-0.09	0.06	0.78	0.76	0.04	9.86E-02	Dry age-related macular degeneration (includes geographic atrophy)	NA	X-11850 levels	0.02	2.99E-06
rs12433497	T	C	0.09	-0.02	0.21	0.28	0.03	6.26E-01	Dry age-related macular degeneration (includes geographic atrophy)	NA	X-11850 levels	0.02	9.91E-06
rs138886343	A	G	-0.2	0.14	0.04	0.09	0.05	8.39E-03	Dry age-related macular degeneration (includes geographic atrophy)	NA	X-11850 levels	0.04	6.21E-07
rs142840963	C	T	0.21	-0.02	0.03	0.03	0.08	8.40E-01	Dry age-related macular degeneration (includes geographic atrophy)	NA	X-11850 levels	0.05	6.88E-06
rs17348202	C	T	0.15	-0.08	0.05	0.03	0.08	3.53E-01	Dry age-related macular degeneration (includes geographic atrophy)	NA	X-11850 levels	0.03	9.20E-06
rs181782952	C	A	-0.32	-0.04	0.01	0.03	0.1	6.45E-01	Dry age-related macular degeneration (includes geographic atrophy)	NA	X-11850 levels	0.07	3.05E-06
rs3884707	T	G	0.08	-0.01	0.26	0.3	0.03	7.19E-01	Dry age-related macular degeneration (includes geographic atrophy)	NA	X-11850 levels	0.02	4.54E-06
rs4576774	C	A	0.07	-0.03	0.35	0.28	0.03	3.51E-01	Dry age-related macular degeneration (includes geographic atrophy)	NA	X-11850 levels	0.02	6.32E-06
rs4831602	T	C	-0.13	0.04	0.92	0.93	0.06	4.65E-01	Dry age-related macular degeneration (includes geographic atrophy)	NA	X-11850 levels	0.03	7.61E-06
rs4858791	T	C	0.2	-0.17	0.97	0.96	0.08	3.61E-02	Dry age-related macular	NA	X-11850 levels	0.04	3.20E-06

								degeneration (includes geographic atrophy)				
rs58280688	C	G	-0.22	-0.04	0.03	0.03	0.09	6.96E-01	Dry age-related macular degeneration (includes geographic atrophy)	NA	X-11850 levels	0.05	7.49E-06
rs62512054	C	T	-0.33	0.25	0.01	0.01	0.18	1.79E-01	Dry age-related macular degeneration (includes geographic atrophy)	NA	X-11850 levels	0.07	5.28E-06
rs72676435	T	C	-0.17	0	0.05	0.05	0.07	9.69E-01	Dry age-related macular degeneration (includes geographic atrophy)	NA	X-11850 levels	0.04	5.19E-06
rs75195552	A	G	-0.09	0.02	0.24	0.26	0.03	6.42E-01	Dry age-related macular degeneration (includes geographic atrophy)	NA	X-11850 levels	0.02	1.40E-06
rs76033483	T	C	0.21	-0.02	0.03	0.04	0.08	7.72E-01	Dry age-related macular degeneration (includes geographic atrophy)	NA	X-11850 levels	0.05	6.65E-06
rs7664690	C	T	-0.09	0.08	0.17	0.26	0.03	1.36E-02	Dry age-related macular degeneration (includes geographic atrophy)	NA	X-11850 levels	0.02	7.04E-06
rs76917355	A	G	0.14	0.01	0.07	0.1	0.05	7.97E-01	Dry age-related macular degeneration (includes geographic atrophy)	NA	X-11850 levels	0.03	3.44E-06
rs79449392	A	G	0.32	-0.15	0.01	0.02	0.12	2.04E-01	Dry age-related macular degeneration (includes geographic atrophy)	NA	X-11850 levels	0.07	3.75E-06
rs10051746	C	T	-0.08	0.04	0.36	0.35	0.03	2.75E-01	Wet age-related macular degeneration	NA	Xylose levels	0.02	3.18E-06
rs10950862	G	T	-0.08	0.03	0.35	0.4	0.03	4.15E-01	Wet age-related macular degeneration	NA	Xylose levels	0.02	4.33E-06
rs112249340	A	G	0.3	-0.37	0.02	0	0.4	3.57E-01	Wet age-related macular degeneration	NA	Xylose levels	0.07	9.24E-06
rs113442137	A	G	0.17	-0.08	0.06	0.08	0.06	1.56E-01	Wet age-related macular	NA	Xylose levels	0.04	6.10E-06

								degeneration				
rs115522284	A	G	-0.19	0.03	0.04	0.05	0.07	6.44E-01	Wet age-related macular degeneration	NA	Xylose levels	0.04	6.30E-06
rs12429666	A	G	0.34	-0.09	0.02	0.02	0.12	4.61E-01	Wet age-related macular degeneration	NA	Xylose levels	0.06	5.33E-08
rs12926601	T	C	0.1	-0.06	0.21	0.14	0.05	1.77E-01	Wet age-related macular degeneration	NA	Xylose levels	0.02	2.04E-06
rs12979487	G	A	0.3	-0.02	0.02	0.01	0.16	9.21E-01	Wet age-related macular degeneration	NA	Xylose levels	0.07	3.59E-06
rs13213275	T	C	0.08	-0.02	0.47	0.52	0.03	5.42E-01	Wet age-related macular degeneration	NA	Xylose levels	0.02	5.68E-06
rs138019197	A	G	0.55	-0.28	0	0.01	0.14	5.07E-02	Wet age-related macular degeneration	NA	Xylose levels	0.12	8.29E-06
rs145187377	T	C	0.15	-0.02	0.07	0.09	0.06	7.68E-01	Wet age-related macular degeneration	NA	Xylose levels	0.03	5.23E-06
rs178730	T	A	-0.09	0	0.24	0.22	0.04	9.02E-01	Wet age-related macular degeneration	NA	Xylose levels	0.02	6.22E-06
rs1789555	G	A	0.24	0.07	0.02	0.01	0.17	6.97E-01	Wet age-related macular degeneration	NA	Xylose levels	0.05	9.93E-06
rs180932555	T	A	0.27	0.21	0.02	0.01	0.17	2.34E-01	Wet age-related macular degeneration	rs79137056	Xylose levels	0.06	7.75E-06
rs1911963	G	A	0.11	-0.04	0.8	0.74	0.04	2.70E-01	Wet age-related macular degeneration	NA	Xylose levels	0.02	2.68E-07
rs224946	A	G	-0.18	0.12	0.94	0.95	0.08	1.35E-01	Wet age-related macular degeneration	NA	Xylose levels	0.04	3.15E-06
rs2291265	T	C	0.11	-0.01	0.88	0.92	0.06	8.04E-01	Wet age-related macular degeneration	NA	Xylose levels	0.03	7.60E-06
rs533213061	G	A	-0.28	0.1	0.02	0.05	0.07	1.80E-01	Wet age-related macular degeneration	NA	Xylose levels	0.06	8.03E-06
rs56367544	C	T	0.3	-0.17	0.02	0	0.25	4.89E-01	Wet age-related macular degeneration	NA	Xylose levels	0.07	8.03E-06
rs62331361	T	C	0.31	-0.03	0.02	0.02	0.11	7.54E-01	Wet age-related macular degeneration	NA	Xylose levels	0.07	5.95E-06

rs62441094	A	G	-0.24	0.45	0.03	0.01	0.19	1.87E-02	Wet age-related macular degeneration	NA	Xylose levels	0.05	6.71E-06
rs7149187	A	G	0.09	-0.03	0.29	0.29	0.04	4.42E-01	Wet age-related macular degeneration	NA	Xylose levels	0.02	5.65E-06
rs72817256	C	G	0.17	-0.08	0.05	0.06	0.07	2.10E-01	Wet age-related macular degeneration	NA	Xylose levels	0.04	5.78E-06
rs79930322	A	G	0.4	-0.07	0.01	0.03	0.1	4.65E-01	Wet age-related macular degeneration	NA	Xylose levels	0.08	5.46E-07
rs80310084	C	A	0.3	-0.12	0.02	0.07	0.07	7.20E-02	Wet age-related macular degeneration	NA	Xylose levels	0.06	1.23E-07
rs10128455	C	T	-0.11	-0.04	0.48	0.47	0.03	1.86E-01	Wet age-related macular degeneration	NA	16a-hydroxy DHEA 3-sulfate levels	0.01	7.23E-13
rs115397758	A	G	0.34	-0.14	0.01	0.01	0.17	4.01E-01	Wet age-related macular degeneration	NA	16a-hydroxy DHEA 3-sulfate levels	0.07	1.13E-06
rs117156815	A	G	0.18	-0.05	0.04	0.05	0.07	5.18E-01	Wet age-related macular degeneration	NA	16a-hydroxy DHEA 3-sulfate levels	0.04	4.44E-06
rs117209214	A	G	0.57	-0.06	0.01	0.03	0.1	5.45E-01	Wet age-related macular degeneration	NA	16a-hydroxy DHEA 3-sulfate levels	0.08	1.55E-11
rs12029116	A	G	0.08	-0.03	0.3	0.36	0.03	3.54E-01	Wet age-related macular degeneration	NA	16a-hydroxy DHEA 3-sulfate levels	0.02	2.43E-06
rs12825645	G	A	0.24	0.21	0.02	0.02	0.13	1.11E-01	Wet age-related macular degeneration	NA	16a-hydroxy DHEA 3-sulfate levels	0.05	7.13E-06
rs13261522	T	A	0.08	0.06	0.22	0.27	0.04	1.07E-01	Wet age-related macular degeneration	NA	16a-hydroxy DHEA 3-sulfate levels	0.02	5.55E-06
rs138761606	C	T	-0.31	0.38	0.01	0	0.51	4.58E-01	Wet age-related macular degeneration	NA	16a-hydroxy DHEA 3-sulfate	0.06	1.04E-06

										levels		
rs148982377	C	T	1.04	0.2	0.03	0.09	0.06	4.40E-04	Wet age-related macular degeneration	NA	16a-hydroxy DHEA 3-sulfate levels	0.04	1.95E-159
rs17171525	A	C	0.18	0.03	0.04	0.03	0.1	7.31E-01	Wet age-related macular degeneration	NA	16a-hydroxy DHEA 3-sulfate levels	0.04	1.74E-06
rs17361671	A	G	-0.08	0	0.23	0.19	0.04	9.66E-01	Wet age-related macular degeneration	NA	16a-hydroxy DHEA 3-sulfate levels	0.02	4.33E-06
rs190372499	G	A	0.29	0.42	0.01	0.02	0.11	2.42E-04	Wet age-related macular degeneration	NA	16a-hydroxy DHEA 3-sulfate levels	0.06	4.46E-06
rs1987580	C	A	-0.09	-0.02	0.29	0.35	0.03	5.55E-01	Wet age-related macular degeneration	NA	16a-hydroxy DHEA 3-sulfate levels	0.02	7.08E-08
rs2810507	A	G	0.25	0.14	0.02	0.02	0.12	2.61E-01	Wet age-related macular degeneration	NA	16a-hydroxy DHEA 3-sulfate levels	0.05	9.42E-07
rs356241	G	A	0.07	0.02	0.54	0.56	0.03	6.37E-01	Wet age-related macular degeneration	NA	16a-hydroxy DHEA 3-sulfate levels	0.01	4.61E-06
rs487624	A	C	-0.08	0.03	0.43	0.49	0.03	4.18E-01	Wet age-related macular degeneration	NA	16a-hydroxy DHEA 3-sulfate levels	0.02	7.53E-08
rs4924416	T	G	0.07	0.02	0.34	0.37	0.03	5.07E-01	Wet age-related macular degeneration	NA	16a-hydroxy DHEA 3-sulfate levels	0.02	2.16E-06
rs61944202	T	C	0.32	0.02	0.01	0.02	0.12	8.68E-01	Wet age-related macular degeneration	NA	16a-hydroxy DHEA 3-sulfate levels	0.07	8.25E-06
rs74910762	A	C	-0.16	-0.06	0.05	0.05	0.08	4.33E-01	Wet age-related macular degeneration	NA	16a-hydroxy DHEA 3-sulfate	0.03	8.68E-07

										levels		
rs9322313	T	C	0.07	0.01	0.4	0.34	0.03	7.84E-01	Wet age-related macular degeneration	NA	16a-hydroxy DHEA 3-sulfate levels	0.02	7.30E-06
rs9526196	A	G	0.12	0.11	0.09	0.04	0.08	1.53E-01	Wet age-related macular degeneration	NA	16a-hydroxy DHEA 3-sulfate levels	0.03	2.99E-06
rs9543004	C	T	0.07	-0.02	0.36	0.39	0.03	5.33E-01	Wet age-related macular degeneration	NA	16a-hydroxy DHEA 3-sulfate levels	0.02	6.85E-06
rs10146121	C	A	-0.07	-0.04	0.68	0.62	0.03	2.13E-01	Wet age-related macular degeneration	rs8018830	1-stearoyl-GPE (18:0) levels	0.02	9.21E-06
rs10234749	G	T	0.08	0.02	0.77	0.67	0.03	5.75E-01	Wet age-related macular degeneration	NA	1-stearoyl-GPE (18:0) levels	0.02	3.70E-06
rs11063303	T	G	-0.23	0.06	0.02	0.02	0.12	6.06E-01	Wet age-related macular degeneration	NA	1-stearoyl-GPE (18:0) levels	0.05	7.64E-06
rs117109614	C	T	-0.3	0.53	0.01	0	0.24	2.85E-02	Wet age-related macular degeneration	NA	1-stearoyl-GPE (18:0) levels	0.06	2.62E-06
rs117940165	G	T	0.28	-0.02	0.02	0.04	0.09	7.86E-01	Wet age-related macular degeneration	NA	1-stearoyl-GPE (18:0) levels	0.06	4.47E-06
rs12292477	T	C	0.11	0.06	0.11	0.11	0.05	2.52E-01	Wet age-related macular degeneration	NA	1-stearoyl-GPE (18:0) levels	0.02	5.94E-06
rs12658974	A	C	0.11	-0.03	0.12	0.14	0.05	5.42E-01	Wet age-related macular degeneration	NA	1-stearoyl-GPE (18:0) levels	0.02	2.16E-06
rs12904030	T	C	-0.07	0.01	0.51	0.42	0.03	8.71E-01	Wet age-related macular degeneration	NA	1-stearoyl-GPE (18:0) levels	0.02	8.83E-06
rs13130041	T	C	0.08	-0.04	0.27	0.22	0.04	2.69E-01	Wet age-related macular degeneration	NA	1-stearoyl-GPE (18:0) levels	0.02	9.55E-06
rs1322199	A	G	0.08	-0.03	0.7	0.79	0.04	5.20E-01	Wet age-related macular degeneration	NA	1-stearoyl-GPE (18:0) levels	0.02	6.41E-06
rs1323678	A	C	-0.07	-0.01	0.49	0.45	0.03	6.99E-01	Wet age-related macular degeneration	NA	1-stearoyl-GPE (18:0) levels	0.02	9.59E-06
rs13263105	G	C	-0.07	0.03	0.66	0.62	0.03	3.88E-01	Wet age-related macular	NA	1-stearoyl-GPE	0.02	3.82E-06

								degeneration		(18:0) levels		
rs135922	A	G	0.07	-0.04	0.49	0.48	0.03	1.88E-01	Wet age-related macular degeneration	NA	1-stearoyl-GPE (18:0) levels	0.02	1.56E-06
rs141999807	G	A	0.25	0.05	0.02	0.03	0.09	5.42E-01	Wet age-related macular degeneration	NA	1-stearoyl-GPE (18:0) levels	0.05	2.14E-06
rs142999532	C	G	0.25	0.3	0.02	0	0.26	2.50E-01	Wet age-related macular degeneration	NA	1-stearoyl-GPE (18:0) levels	0.06	8.27E-06
rs144767726	A	C	-0.2	-0.16	0.03	0.03	0.1	1.09E-01	Wet age-related macular degeneration	NA	1-stearoyl-GPE (18:0) levels	0.04	5.71E-06
rs16979884	A	G	0.11	-0.09	0.1	0.09	0.06	1.33E-01	Wet age-related macular degeneration	NA	1-stearoyl-GPE (18:0) levels	0.03	7.81E-06
rs174560	C	T	-0.09	0.01	0.31	0.39	0.03	6.92E-01	Wet age-related macular degeneration	NA	1-stearoyl-GPE (18:0) levels	0.02	1.52E-08
rs17665744	G	T	0.1	-0.03	0.16	0.16	0.04	5.74E-01	Wet age-related macular degeneration	NA	1-stearoyl-GPE (18:0) levels	0.02	1.55E-06
rs1858942	A	G	0.07	-0.01	0.42	0.39	0.03	8.56E-01	Wet age-related macular degeneration	NA	1-stearoyl-GPE (18:0) levels	0.02	2.23E-06
rs1866637	T	C	0.08	0.02	0.23	0.2	0.04	5.65E-01	Wet age-related macular degeneration	NA	1-stearoyl-GPE (18:0) levels	0.02	4.14E-06
rs2004196	T	C	0.1	0.01	0.14	0.22	0.04	7.24E-01	Wet age-related macular degeneration	NA	1-stearoyl-GPE (18:0) levels	0.02	1.33E-06
rs2540682	A	T	0.09	0	0.18	0.16	0.04	9.86E-01	Wet age-related macular degeneration	NA	1-stearoyl-GPE (18:0) levels	0.02	9.59E-06
rs2935412	T	C	-0.1	0.04	0.85	0.85	0.05	3.71E-01	Wet age-related macular degeneration	NA	1-stearoyl-GPE (18:0) levels	0.02	9.68E-06
rs35853021	T	G	0.21	-0.11	0.35	0.4	0.03	7.16E-04	Wet age-related macular degeneration	NA	1-stearoyl-GPE (18:0) levels	0.02	1.38E-40
rs3779773	C	G	0.1	0.03	0.16	0.14	0.05	5.36E-01	Wet age-related macular degeneration	NA	1-stearoyl-GPE (18:0) levels	0.02	1.79E-06
rs526018	G	A	-0.08	-0.03	0.28	0.35	0.03	4.49E-01	Wet age-related macular degeneration	NA	1-stearoyl-GPE (18:0) levels	0.02	8.99E-07
rs62036205	C	A	-0.13	0.07	0.09	0.1	0.06	1.79E-01	Wet age-related macular degeneration	NA	1-stearoyl-GPE (18:0) levels	0.03	9.36E-07

rs633695	G	A	0.17	-0.13	0.28	0.31	0.04	3.82E-04	Wet age-related macular degeneration	NA	1-stearoyl-GPE (18:0) levels	0.02	1.41E-23
rs6999569	G	A	-0.08	0.04	0.48	0.46	0.03	2.24E-01	Wet age-related macular degeneration	NA	1-stearoyl-GPE (18:0) levels	0.02	2.71E-07
rs7488822	G	A	0.4	-0.03	0.01	0.01	0.19	8.58E-01	Wet age-related macular degeneration	NA	1-stearoyl-GPE (18:0) levels	0.09	3.81E-06
rs77692382	T	C	0.18	0	0.25	0.2	0.04	9.73E-01	Wet age-related macular degeneration	NA	1-stearoyl-GPE (18:0) levels	0.02	5.77E-26
rs79696139	A	G	0.19	0.01	0.04	0.04	0.08	8.79E-01	Wet age-related macular degeneration	NA	1-stearoyl-GPE (18:0) levels	0.04	2.27E-06
rs8058610	T	C	0.07	-0.04	0.6	0.55	0.03	2.64E-01	Wet age-related macular degeneration	NA	1-stearoyl-GPE (18:0) levels	0.02	3.39E-06
rs10228292	T	C	-0.07	-0.01	0.69	0.72	0.04	7.94E-01	Wet age-related macular degeneration	NA	DHEAS levels	0.01	7.29E-06
rs10494157	A	G	-0.09	0.02	0.13	0.09	0.06	7.04E-01	Wet age-related macular degeneration	NA	DHEAS levels	0.02	3.68E-06
rs10806995	G	A	-0.07	0.04	0.3	0.41	0.03	2.81E-01	Wet age-related macular degeneration	NA	DHEAS levels	0.01	7.79E-06
rs1111474	T	C	0.12	-0.04	0.07	0.07	0.06	5.58E-01	Wet age-related macular degeneration	NA	DHEAS levels	0.03	9.09E-06
rs115255559	G	A	-0.22	0.18	0.02	0.01	0.14	2.01E-01	Wet age-related macular degeneration	NA	DHEAS levels	0.05	3.52E-06
rs117833456	C	T	-0.17	0.14	0.03	0.06	0.07	5.45E-02	Wet age-related macular degeneration	NA	DHEAS levels	0.04	8.47E-06
rs12049195	A	G	-0.07	0.01	0.53	0.54	0.03	7.27E-01	Wet age-related macular degeneration	NA	DHEAS levels	0.01	4.80E-07
rs12658172	C	G	-0.08	0.05	0.16	0.15	0.05	2.80E-01	Wet age-related macular degeneration	NA	DHEAS levels	0.02	8.11E-06
rs141370991	T	C	-0.24	-0.01	0.02	0.03	0.1	9.29E-01	Wet age-related macular degeneration	NA	DHEAS levels	0.05	5.68E-07
rs142295380	G	C	-0.33	-0.02	0.01	0.02	0.12	8.64E-01	Wet age-related macular degeneration	NA	DHEAS levels	0.07	4.92E-06
rs144814135	A	G	-0.24	-0.03	0.02	0.01	0.14	8.36E-01	Wet age-related macular	NA	DHEAS levels	0.05	4.85E-06

								degeneration				
rs148769396	A	G	0.23	-0.25	0.02	0.02	0.12	3.43E-02	Wet age-related macular degeneration	NA	DHEAS levels	0.05	9.77E-06
rs148982377	C	T	-0.42	0.2	0.03	0.09	0.06	4.40E-04	Wet age-related macular degeneration	NA	DHEAS levels	0.04	7.69E-31
rs149982314	T	C	-0.36	-0.36	0.01	0	0.38	3.40E-01	Wet age-related macular degeneration	NA	DHEAS levels	0.07	2.21E-07
rs150328525	G	T	-0.3	0.15	0.01	0.02	0.11	1.80E-01	Wet age-related macular degeneration	NA	DHEAS levels	0.07	5.51E-06
rs150374297	C	T	-0.26	-0.13	0.01	0.01	0.2	5.24E-01	Wet age-related macular degeneration	NA	DHEAS levels	0.06	4.27E-06
rs1807334	A	G	0.06	0.07	0.4	0.36	0.03	3.10E-02	Wet age-related macular degeneration	NA	DHEAS levels	0.01	5.00E-06
rs184445849	T	C	0.25	0.11	0.02	0.01	0.15	4.39E-01	Wet age-related macular degeneration	NA	DHEAS levels	0.05	4.11E-06
rs191866531	A	G	0.33	0.12	0.01	0.01	0.15	4.13E-01	Wet age-related macular degeneration	NA	DHEAS levels	0.07	7.58E-06
rs2055157	A	C	0.08	0.1	0.19	0.15	0.05	3.27E-02	Wet age-related macular degeneration	NA	DHEAS levels	0.02	4.40E-06
rs212100	C	T	0.09	-0.12	0.84	0.85	0.05	1.10E-02	Wet age-related macular degeneration	NA	DHEAS levels	0.02	3.34E-06
rs28552668	T	C	-0.12	-0.03	0.07	0.07	0.06	5.99E-01	Wet age-related macular degeneration	NA	DHEAS levels	0.03	4.92E-06
rs529543	G	T	-0.06	-0.05	0.47	0.54	0.03	1.02E-01	Wet age-related macular degeneration	NA	DHEAS levels	0.01	2.85E-06
rs560835392	A	C	0.21	-0.2	0.02	0.01	0.15	1.78E-01	Wet age-related macular degeneration	NA	DHEAS levels	0.04	3.25E-06
rs6428823	T	C	-0.06	0.02	0.56	0.64	0.03	6.46E-01	Wet age-related macular degeneration	NA	DHEAS levels	0.01	3.55E-06
rs659964	C	G	0.09	-0.04	0.15	0.15	0.05	3.65E-01	Wet age-related macular degeneration	NA	DHEAS levels	0.02	2.89E-06
rs6879686	T	G	-0.07	0.03	0.23	0.2	0.04	4.57E-01	Wet age-related macular degeneration	NA	DHEAS levels	0.02	6.21E-06

rs706795	C	T	0.06	-0.04	0.59	0.59	0.03	1.75E-01	Wet age-related macular degeneration	NA	DHEAS levels	0.01	7.83E-06
rs71606471	A	C	-0.11	0.13	0.1	0.05	0.07	8.41E-02	Wet age-related macular degeneration	rs13184105	DHEAS levels	0.02	1.62E-06
rs7173332	C	G	-0.09	0.05	0.16	0.19	0.04	2.25E-01	Wet age-related macular degeneration	NA	DHEAS levels	0.02	2.48E-06
rs73172224	A	G	-0.09	0.04	0.13	0.18	0.04	4.06E-01	Wet age-related macular degeneration	NA	DHEAS levels	0.02	3.07E-06
rs74041508	C	G	-0.08	0.04	0.18	0.12	0.05	4.34E-01	Wet age-related macular degeneration	NA	DHEAS levels	0.02	7.74E-06
rs75280042	T	A	-0.18	-0.02	0.03	0.02	0.1	8.32E-01	Wet age-related macular degeneration	NA	DHEAS levels	0.04	7.78E-06
rs75561083	T	C	0.24	-0.13	0.02	0.07	0.06	4.13E-02	Wet age-related macular degeneration	NA	DHEAS levels	0.05	8.37E-06
rs76294479	T	C	0.16	-0.02	0.07	0.04	0.09	8.25E-01	Wet age-related macular degeneration	NA	DHEAS levels	0.03	2.21E-09
rs76768009	A	G	-0.16	-0.02	0.04	0.01	0.17	9.05E-01	Wet age-related macular degeneration	NA	DHEAS levels	0.04	9.55E-06
rs7687817	T	C	-0.11	0.01	0.09	0.05	0.07	8.46E-01	Wet age-related macular degeneration	NA	DHEAS levels	0.02	3.26E-06
rs76895520	A	G	-0.21	-0.3	0.02	0.01	0.16	6.95E-02	Wet age-related macular degeneration	NA	DHEAS levels	0.05	7.38E-06
rs784420	G	A	0.08	-0.04	0.3	0.29	0.04	2.37E-01	Wet age-related macular degeneration	NA	DHEAS levels	0.01	1.43E-08
rs7855576	A	C	-0.07	-0.04	0.28	0.27	0.04	2.77E-01	Wet age-related macular degeneration	NA	DHEAS levels	0.02	3.21E-06
rs79317397	A	C	0.24	0.19	0.02	0	0.25	4.51E-01	Wet age-related macular degeneration	NA	DHEAS levels	0.05	2.83E-06
rs9420589	T	G	-0.06	0.02	0.43	0.45	0.03	5.26E-01	Wet age-related macular degeneration	NA	DHEAS levels	0.01	1.84E-06
rs955783	T	A	0.09	-0.03	0.4	0.37	0.03	3.09E-01	Wet age-related macular degeneration	NA	DHEAS levels	0.01	4.92E-10
rs9594288	T	C	-0.17	0.06	0.03	0.02	0.1	5.53E-01	Wet age-related macular	NA	DHEAS levels	0.04	6.95E-06

									degeneration				
rs10495913	A	G	0.07	0.03	0.62	0.63	0.03	3.73E-01	Wet age-related macular degeneration	NA	X-13553 levels	0.01	4.85E-06
rs10840516	A	G	0.09	0.07	0.23	0.2	0.04	6.40E-02	Wet age-related macular degeneration	NA	X-13553 levels	0.02	5.61E-08
rs113941374	G	A	0.16	0.01	0.04	0.03	0.1	9.32E-01	Wet age-related macular degeneration	NA	X-13553 levels	0.04	5.06E-06
rs114819259	G	C	-0.28	-0.14	0.01	0.03	0.1	1.67E-01	Wet age-related macular degeneration	NA	X-13553 levels	0.06	3.14E-06
rs117242663	A	G	-0.29	-0.32	0.01	0.01	0.16	4.80E-02	Wet age-related macular degeneration	NA	X-13553 levels	0.06	2.77E-06
rs12228698	G	A	-0.13	-0.03	0.1	0.14	0.05	4.72E-01	Wet age-related macular degeneration	NA	X-13553 levels	0.02	1.62E-07
rs12492113	A	G	-0.1	-0.08	0.13	0.15	0.05	7.63E-02	Wet age-related macular degeneration	NA	X-13553 levels	0.02	6.60E-06
rs13228009	G	A	0.06	0	0.6	0.6	0.03	9.68E-01	Wet age-related macular degeneration	NA	X-13553 levels	0.01	9.56E-06
rs139448040	A	G	0.27	0	0.01	0.02	0.13	9.88E-01	Wet age-related macular degeneration	NA	X-13553 levels	0.06	5.94E-06
rs141993770	C	T	-0.3	-0.02	0.01	0.02	0.11	8.57E-01	Wet age-related macular degeneration	NA	X-13553 levels	0.07	6.13E-06
rs142402970	T	C	0.43	-0.38	0.01	0	0.75	6.08E-01	Wet age-related macular degeneration	NA	X-13553 levels	0.09	4.38E-06
rs144098166	G	A	0.34	0.19	0.01	0.01	0.14	1.79E-01	Wet age-related macular degeneration	NA	X-13553 levels	0.07	1.62E-06
rs147712721	C	G	-0.27	-0.03	0.02	0.04	0.09	7.29E-01	Wet age-related macular degeneration	NA	X-13553 levels	0.05	2.55E-08
rs186802061	T	G	-0.41	-0.18	0.01	0.02	0.13	1.82E-01	Wet age-related macular degeneration	NA	X-13553 levels	0.09	6.84E-06
rs2270524	T	C	0.19	0.06	0.03	0.07	0.06	3.43E-01	Wet age-related macular degeneration	NA	X-13553 levels	0.04	3.30E-06
rs2916716	T	A	-0.07	-0.02	0.35	0.31	0.04	6.12E-01	Wet age-related macular degeneration	NA	X-13553 levels	0.02	3.61E-06

rs2963950	T	C	0.07	0.06	0.33	0.34	0.03	9.76E-02	Wet age-related macular degeneration	NA	X-13553 levels	0.02	8.04E-06
rs3121429	C	T	-0.11	-0.22	0.89	0.96	0.08	5.61E-03	Wet age-related macular degeneration	NA	X-13553 levels	0.02	5.59E-06
rs4415228	A	G	0.06	-0.03	0.55	0.6	0.03	4.39E-01	Wet age-related macular degeneration	NA	X-13553 levels	0.01	5.95E-06
rs4704097	G	A	-0.07	-0.02	0.55	0.55	0.03	6.41E-01	Wet age-related macular degeneration	NA	X-13553 levels	0.01	4.14E-06
rs503223	A	G	0.45	0.11	0	0.04	0.09	2.32E-01	Wet age-related macular degeneration	NA	X-13553 levels	0.1	8.96E-06
rs72972188	C	T	0.11	-0.03	0.1	0.1	0.06	6.14E-01	Wet age-related macular degeneration	NA	X-13553 levels	0.02	8.25E-06
rs77438259	A	C	-0.26	-0.1	0.02	0.01	0.15	5.04E-01	Wet age-related macular degeneration	NA	X-13553 levels	0.06	2.65E-06
rs8080108	C	T	0.09	-0.02	0.31	0.35	0.03	5.37E-01	Wet age-related macular degeneration	NA	X-13553 levels	0.02	1.60E-08
rs10786400	G	A	-0.15	0.03	0.32	0.3	0.04	3.56E-01	Wet age-related macular degeneration	NA	N2-acetyl,N6,N6 -dimethyllysine levels	0.02	4.79E-18
rs10883082	T	C	0.92	-0.1	0.6	0.56	0.03	2.58E-03	Wet age-related macular degeneration	NA	N2-acetyl,N6,N6 -dimethyllysine levels	0.01	1.00E-200
rs111332525	T	C	-0.25	-0.02	0.02	0.03	0.1	8.57E-01	Wet age-related macular degeneration	NA	N2-acetyl,N6,N6 -dimethyllysine levels	0.06	6.29E-06
rs113851727	A	T	-0.35	0.11	0.05	0.1	0.06	6.06E-02	Wet age-related macular degeneration	NA	N2-acetyl,N6,N6 -dimethyllysine levels	0.04	4.01E-20
rs117564334	G	C	0.11	-0.03	0.14	0.2	0.04	4.50E-01	Wet age-related macular degeneration	NA	N2-acetyl,N6,N6 -dimethyllysine levels	0.02	2.93E-06
rs11861590	C	T	-0.07	0.06	0.52	0.52	0.03	7.61E-02	Wet age-related macular degeneration	NA	N2-acetyl,N6,N6 -dimethyllysine	0.02	9.79E-06

										levels		
rs12190308	G	A	-0.1	-0.02	0.14	0.12	0.05	7.24E-01	Wet age-related macular degeneration	NA	N2-acetyl,N6,N6-dimethyllysine levels	0.02	6.02E-06
rs12284998	C	T	0.08	-0.06	0.34	0.39	0.03	6.80E-02	Wet age-related macular degeneration	NA	N2-acetyl,N6,N6-dimethyllysine levels	0.02	4.76E-06
rs12420129	T	C	-0.28	-0.1	0.02	0.09	0.06	8.64E-02	Wet age-related macular degeneration	NA	N2-acetyl,N6,N6-dimethyllysine levels	0.06	1.39E-06
rs13408904	T	C	0.19	-0.12	0.04	0.06	0.07	9.17E-02	Wet age-related macular degeneration	rs17860416	N2-acetyl,N6,N6-dimethyllysine levels	0.04	3.51E-06
rs13538	G	A	0.64	-0.03	0.23	0.18	0.04	5.01E-01	Wet age-related macular degeneration	NA	N2-acetyl,N6,N6-dimethyllysine levels	0.02	1.00E-200
rs143083364	G	C	0.26	0.05	0.02	0.03	0.09	6.00E-01	Wet age-related macular degeneration	NA	N2-acetyl,N6,N6-dimethyllysine levels	0.06	5.78E-06
rs1490101	T	C	0.12	0.02	0.09	0.09	0.06	7.12E-01	Wet age-related macular degeneration	NA	N2-acetyl,N6,N6-dimethyllysine levels	0.03	9.63E-06
rs180851257	C	T	-0.29	0.02	0.02	0.03	0.09	8.09E-01	Wet age-related macular degeneration	NA	N2-acetyl,N6,N6-dimethyllysine levels	0.06	8.75E-06
rs183646569	A	G	-0.21	0.03	0.03	0.06	0.07	6.97E-01	Wet age-related macular degeneration	NA	N2-acetyl,N6,N6-dimethyllysine levels	0.05	2.88E-06
rs183739917	C	G	-0.67	0.04	0	0.02	0.12	7.51E-01	Wet age-related macular degeneration	NA	N2-acetyl,N6,N6-dimethyllysine levels	0.14	2.76E-06
rs1925647	C	A	0.12	0.02	0.27	0.29	0.04	5.86E-01	Wet age-related macular degeneration	NA	N2-acetyl,N6,N6-dimethyllysine	0.02	1.04E-11

										levels		
rs34580553	A	G	0.12	0.06	0.1	0.06	0.07	3.73E-01	Wet age-related macular degeneration	NA	N2-acetyl,N6,N6 -dimethyllysine levels	0.03	3.80E-06
rs512187	A	G	-0.07	0.01	0.56	0.64	0.03	8.42E-01	Wet age-related macular degeneration	NA	N2-acetyl,N6,N6 -dimethyllysine levels	0.02	5.47E-06
rs55710329	T	C	0.12	0.01	0.1	0.1	0.05	9.25E-01	Wet age-related macular degeneration	NA	N2-acetyl,N6,N6 -dimethyllysine levels	0.03	6.38E-06
rs62481075	T	C	0.19	0	0.04	0.02	0.11	9.90E-01	Wet age-related macular degeneration	NA	N2-acetyl,N6,N6 -dimethyllysine levels	0.04	1.79E-06
rs6584281	G	A	-0.1	-0.05	0.51	0.53	0.03	1.08E-01	Wet age-related macular degeneration	NA	N2-acetyl,N6,N6 -dimethyllysine levels	0.02	1.10E-10
rs7609407	G	A	-0.13	0	0.15	0.1	0.06	9.79E-01	Wet age-related macular degeneration	NA	N2-acetyl,N6,N6 -dimethyllysine levels	0.02	3.23E-09
rs79816080	C	T	0.21	-0.14	0.03	0.02	0.11	2.14E-01	Wet age-related macular degeneration	NA	N2-acetyl,N6,N6 -dimethyllysine levels	0.05	7.38E-06
rs10952288	G	A	0.09	0.05	0.47	0.36	0.03	1.61E-01	Wet age-related macular degeneration	NA	Succinimide levels	0.02	1.32E-06
rs112406293	A	G	0.19	0.05	0.05	0.11	0.05	3.82E-01	Wet age-related macular degeneration	NA	Succinimide levels	0.04	2.35E-06
rs11920360	C	T	0.36	0.46	0.01	0.01	0.15	1.96E-03	Wet age-related macular degeneration	NA	Succinimide levels	0.08	1.96E-06
rs12125124	T	C	0.19	-0.13	0.05	0.05	0.08	8.72E-02	Wet age-related macular degeneration	NA	Succinimide levels	0.04	7.33E-06
rs12439661	C	A	-0.08	-0.04	0.58	0.64	0.03	2.30E-01	Wet age-related macular degeneration	NA	Succinimide levels	0.02	8.82E-06
rs139289471	C	G	0.54	-0.09	0.01	0	0.31	7.72E-01	Wet age-related macular	NA	Succinimide	0.12	5.98E-06

									degeneration		levels		
rs142777799	C	T	0.18	-0.07	0.06	0.08	0.06	2.94E-01	Wet age-related macular degeneration	NA	Succinimide levels	0.04	3.62E-06
rs148866069	G	A	0.43	0.06	0.01	0.02	0.11	5.73E-01	Wet age-related macular degeneration	NA	Succinimide levels	0.1	5.82E-06
rs16871364	T	G	0.15	0.04	0.21	0.26	0.04	2.41E-01	Wet age-related macular degeneration	NA	Succinimide levels	0.02	2.08E-11
rs181190716	T	C	0.7	0.02	0	0.06	0.07	8.33E-01	Wet age-related macular degeneration	NA	Succinimide levels	0.15	3.70E-06
rs183382921	G	A	0.75	0.16	0	0.04	0.09	7.24E-02	Wet age-related macular degeneration	NA	Succinimide levels	0.17	7.49E-06
rs188481956	T	C	0.3	-0.36	0.02	0	0.25	1.53E-01	Wet age-related macular degeneration	NA	Succinimide levels	0.06	2.76E-06
rs2163864	C	T	-0.1	-0.01	0.19	0.22	0.04	8.97E-01	Wet age-related macular degeneration	NA	Succinimide levels	0.02	9.13E-06
rs4462695	A	C	-0.09	-0.01	0.27	0.37	0.03	7.85E-01	Wet age-related macular degeneration	NA	Succinimide levels	0.02	8.14E-06
rs5768637	A	G	0.12	0.07	0.15	0.17	0.04	9.47E-02	Wet age-related macular degeneration	NA	Succinimide levels	0.03	3.87E-06
rs6134892	G	A	0.1	0.05	0.22	0.2	0.04	2.30E-01	Wet age-related macular degeneration	NA	Succinimide levels	0.02	7.23E-06
rs74588150	C	G	0.47	0.16	0.01	0.06	0.07	1.71E-02	Wet age-related macular degeneration	NA	Succinimide levels	0.11	9.79E-06
rs75774670	C	G	0.72	0.11	0	0.02	0.13	3.96E-01	Wet age-related macular degeneration	NA	Succinimide levels	0.16	5.04E-06
rs7640191	T	C	0.27	0.2	0.03	0.06	0.07	7.25E-03	Wet age-related macular degeneration	NA	Succinimide levels	0.05	1.18E-06
rs8056710	A	G	0.47	0.43	0.01	0.01	0.23	6.28E-02	Wet age-related macular degeneration	NA	Succinimide levels	0.1	5.71E-06
rs9368908	G	C	-0.16	-0.06	0.07	0.12	0.05	2.31E-01	Wet age-related macular degeneration	NA	Succinimide levels	0.04	8.32E-06
rs11174150	T	C	0.07	-0.04	0.34	0.29	0.04	3.07E-01	Wet age-related macular degeneration	NA	Androstenediol (3beta,17beta)	0.01	5.64E-06

										monosulfate (1) levels		
rs111936578	C	T	-0.22	0.21	0.02	0	0.24	3.80E-01	Wet age-related macular degeneration	NA	Androstenediol (3beta,17beta) monosulfate (1) levels	0.05	7.46E-06
rs12038241	G	A	-0.06	0.02	0.56	0.64	0.03	6.24E-01	Wet age-related macular degeneration	NA	Androstenediol (3beta,17beta) monosulfate (1) levels	0.01	5.88E-06
rs141370991	T	C	-0.23	-0.01	0.02	0.03	0.1	9.29E-01	Wet age-related macular degeneration	NA	Androstenediol (3beta,17beta) monosulfate (1) levels	0.05	2.02E-06
rs142274824	T	C	-0.33	-0.19	0.01	0.01	0.16	2.34E-01	Wet age-related macular degeneration	NA	Androstenediol (3beta,17beta) monosulfate (1) levels	0.07	9.75E-06
rs144814135	A	G	-0.25	-0.03	0.02	0.01	0.14	8.36E-01	Wet age-related macular degeneration	NA	Androstenediol (3beta,17beta) monosulfate (1) levels	0.05	3.13E-06
rs1467067	C	A	0.07	-0.04	0.31	0.25	0.04	2.40E-01	Wet age-related macular degeneration	NA	Androstenediol (3beta,17beta) monosulfate (1) levels	0.01	3.17E-06
rs146747578	C	G	-0.29	-0.08	0.01	0.01	0.18	6.46E-01	Wet age-related macular degeneration	NA	Androstenediol (3beta,17beta) monosulfate (1) levels	0.07	9.63E-06
rs147871557	G	A	0.32	-0.53	0.01	0	0.48	2.68E-01	Wet age-related macular degeneration	NA	Androstenediol (3beta,17beta) monosulfate (1)	0.07	8.20E-06

											levels		
rs148982377	C	T	-0.38	0.2	0.03	0.09	0.06	4.40E-04	Wet age-related macular degeneration	NA	Androstenediol (3beta,17beta) monosulfate (1) levels	0.04	6.86E-25
rs149982314	T	C	-0.32	-0.36	0.01	0	0.38	3.40E-01	Wet age-related macular degeneration	NA	Androstenediol (3beta,17beta) monosulfate (1) levels	0.07	5.64E-06
rs1871395	G	A	0.11	0.01	0.16	0.28	0.04	8.66E-01	Wet age-related macular degeneration	NA	Androstenediol (3beta,17beta) monosulfate (1) levels	0.02	1.18E-09
rs191866531	A	G	0.42	0.12	0.01	0.01	0.15	4.13E-01	Wet age-related macular degeneration	NA	Androstenediol (3beta,17beta) monosulfate (1) levels	0.07	2.00E-08
rs28524991	A	T	-0.19	0.16	0.03	0.02	0.12	1.85E-01	Wet age-related macular degeneration	NA	Androstenediol (3beta,17beta) monosulfate (1) levels	0.04	3.31E-06
rs55677910	G	A	-0.09	0.05	0.14	0.15	0.05	2.59E-01	Wet age-related macular degeneration	NA	Androstenediol (3beta,17beta) monosulfate (1) levels	0.02	3.07E-06
rs55764900	G	A	-0.09	0.05	0.16	0.12	0.05	2.75E-01	Wet age-related macular degeneration	NA	Androstenediol (3beta,17beta) monosulfate (1) levels	0.02	2.35E-06
rs677027	A	G	-0.06	0.04	0.49	0.49	0.03	2.14E-01	Wet age-related macular degeneration	NA	Androstenediol (3beta,17beta) monosulfate (1) levels	0.01	5.61E-06

rs706795	C	T	0.06	-0.04	0.59	0.59	0.03	1.75E-01	Wet age-related macular degeneration	NA	Androstenediol (3beta,17beta) monosulfate (1) levels	0.01	4.21E-06
rs7086721	C	T	0.07	-0.02	0.7	0.7	0.04	5.47E-01	Wet age-related macular degeneration	NA	Androstenediol (3beta,17beta) monosulfate (1) levels	0.02	9.63E-06
rs7173332	C	G	-0.09	0.05	0.16	0.19	0.04	2.25E-01	Wet age-related macular degeneration	NA	Androstenediol (3beta,17beta) monosulfate (1) levels	0.02	3.07E-06
rs72729813	C	G	-0.13	0.06	0.06	0.05	0.07	4.00E-01	Wet age-related macular degeneration	NA	Androstenediol (3beta,17beta) monosulfate (1) levels	0.03	7.14E-06
rs75107956	C	A	0.16	0.04	0.04	0.06	0.07	6.16E-01	Wet age-related macular degeneration	rs143367698	Androstenediol (3beta,17beta) monosulfate (1) levels	0.04	8.92E-06
rs75561083	T	C	0.26	-0.13	0.02	0.07	0.06	4.13E-02	Wet age-related macular degeneration	NA	Androstenediol (3beta,17beta) monosulfate (1) levels	0.05	3.38E-06
rs76294479	T	C	0.14	-0.02	0.07	0.04	0.09	8.25E-01	Wet age-related macular degeneration	NA	Androstenediol (3beta,17beta) monosulfate (1) levels	0.03	7.79E-08
rs7675318	T	C	0.08	0.07	0.18	0.17	0.04	1.19E-01	Wet age-related macular degeneration	NA	Androstenediol (3beta,17beta) monosulfate (1) levels	0.02	4.19E-06
rs7687817	T	C	-0.11	0.01	0.09	0.05	0.07	8.46E-01	Wet age-related macular	NA	Androstenediol	0.02	5.33E-06

								degeneration		(3beta,17beta) monosulfate (1) levels		
rs78438321	T	C	-0.27	-0.03	0.02	0	0.25	8.94E-01	Wet age-related macular degeneration	NA	Androstenediol (3beta,17beta) monosulfate (1) levels	0.05	5.05E-07
rs784420	G	A	0.07	-0.04	0.3	0.29	0.04	2.37E-01	Wet age-related macular degeneration	NA	Androstenediol (3beta,17beta) monosulfate (1) levels	0.01	8.71E-06
rs9407758	A	C	-0.07	-0.03	0.43	0.42	0.03	3.31E-01	Wet age-related macular degeneration	NA	Androstenediol (3beta,17beta) monosulfate (1) levels	0.01	2.65E-06
rs9420589	T	G	-0.06	0.02	0.43	0.45	0.03	5.26E-01	Wet age-related macular degeneration	NA	Androstenediol (3beta,17beta) monosulfate (1) levels	0.01	3.31E-06
rs955783	T	A	0.07	-0.03	0.4	0.37	0.03	3.09E-01	Wet age-related macular degeneration	NA	Androstenediol (3beta,17beta) monosulfate (1) levels	0.01	2.32E-06
rs11205297	C	T	0.1	-0.03	0.09	0.11	0.05	5.46E-01	Wet age-related macular degeneration	NA	5alpha-androstan-3beta,17beta-diol disulfate levels	0.02	9.53E-06
rs11253048	G	A	0.09	-0.04	0.17	0.16	0.05	3.60E-01	Wet age-related macular degeneration	NA	5alpha-androstan-3beta,17beta-diol disulfate levels	0.02	1.88E-07
rs112881196	G	C	-0.24	0	0.03	0.02	0.11	9.86E-01	Wet age-related macular degeneration	NA	5alpha-androstan-3beta,17beta-di	0.04	8.40E-12

											ol disulfate levels		
rs114974231	C	A	0.14	0.1	0.05	0.04	0.08	2.17E-01	Wet age-related macular degeneration	NA	5alpha-androstan-3beta,17beta-diol disulfate levels	0.03	5.87E-06
rs11742924	A	G	0.06	-0.01	0.41	0.48	0.03	7.22E-01	Wet age-related macular degeneration	NA	5alpha-androstan-3beta,17beta-diol disulfate levels	0.01	7.89E-06
rs117434713	G	A	-0.27	-0.07	0.01	0.02	0.13	5.74E-01	Wet age-related macular degeneration	NA	5alpha-androstan-3beta,17beta-diol disulfate levels	0.06	7.79E-06
rs1256768	G	A	0.09	-0.01	0.17	0.16	0.04	8.00E-01	Wet age-related macular degeneration	NA	5alpha-androstan-3beta,17beta-diol disulfate levels	0.02	5.86E-07
rs13157117	C	T	0.07	-0.02	0.57	0.62	0.03	5.71E-01	Wet age-related macular degeneration	NA	5alpha-androstan-3beta,17beta-diol disulfate levels	0.01	4.69E-07
rs13214463	G	C	-0.17	0.04	0.03	0.01	0.16	7.98E-01	Wet age-related macular degeneration	NA	5alpha-androstan-3beta,17beta-diol disulfate levels	0.04	4.58E-06
rs146609097	G	T	-0.24	-0.02	0.01	0.03	0.09	8.47E-01	Wet age-related macular degeneration	NA	5alpha-androstan-3beta,17beta-diol disulfate levels	0.05	9.27E-06
rs148269658	G	A	-0.22	-0.13	0.02	0.01	0.18	4.78E-01	Wet age-related macular degeneration	NA	5alpha-androstan-3beta,17beta-diol disulfate	0.05	5.73E-06

											levels		
rs148982377	C	T	-0.67	0.2	0.03	0.09	0.06	4.40E-04	Wet age-related macular degeneration	NA	5alpha-androstan-3beta,17beta-diol disulfate levels	0.04	4.30E-79
rs150572962	T	C	-0.25	0.06	0.02	0.02	0.12	6.10E-01	Wet age-related macular degeneration	NA	5alpha-androstan-3beta,17beta-diol disulfate levels	0.05	2.46E-06
rs1562901	A	G	0.06	0	0.53	0.57	0.03	9.04E-01	Wet age-related macular degeneration	NA	5alpha-androstan-3beta,17beta-diol disulfate levels	0.01	6.81E-06
rs16029	A	G	-0.21	0.08	0.02	0.03	0.09	3.71E-01	Wet age-related macular degeneration	NA	5alpha-androstan-3beta,17beta-diol disulfate levels	0.04	3.26E-06
rs1871395	G	A	0.16	0.01	0.16	0.28	0.04	8.66E-01	Wet age-related macular degeneration	NA	5alpha-androstan-3beta,17beta-diol disulfate levels	0.02	2.18E-19
rs1956213	G	C	-0.06	0.01	0.34	0.41	0.03	8.24E-01	Wet age-related macular degeneration	NA	5alpha-androstan-3beta,17beta-diol disulfate levels	0.01	2.79E-06
rs2098759	A	G	-0.07	0.08	0.57	0.66	0.03	2.22E-02	Wet age-related macular degeneration	NA	5alpha-androstan-3beta,17beta-diol disulfate levels	0.01	3.98E-07
rs212100	C	T	0.14	-0.12	0.84	0.85	0.05	1.10E-02	Wet age-related macular degeneration	NA	5alpha-androstan-3beta,17beta-diol disulfate levels	0.02	3.93E-14

rs28460220	G	T	-0.17	-0.09	0.04	0.1	0.06	1.23E-01	Wet age-related macular degeneration	NA	5alpha-androstan-3beta,17beta-diol disulfate levels	0.03	4.59E-07
rs487624	A	C	-0.06	0.03	0.43	0.49	0.03	4.18E-01	Wet age-related macular degeneration	NA	5alpha-androstan-3beta,17beta-diol disulfate levels	0.01	7.06E-06
rs74371056	C	A	-0.08	0.05	0.16	0.14	0.05	3.20E-01	Wet age-related macular degeneration	NA	5alpha-androstan-3beta,17beta-diol disulfate levels	0.02	5.67E-06
rs78481579	A	G	0.14	-0.09	0.04	0.05	0.07	2.12E-01	Wet age-related macular degeneration	NA	5alpha-androstan-3beta,17beta-diol disulfate levels	0.03	7.40E-06
rs78742441	A	G	0.12	0.09	0.08	0.12	0.05	8.31E-02	Wet age-related macular degeneration	NA	5alpha-androstan-3beta,17beta-diol disulfate levels	0.02	1.96E-06
rs78947297	G	A	-0.16	0.12	0.04	0.03	0.09	1.90E-01	Wet age-related macular degeneration	NA	5alpha-androstan-3beta,17beta-diol disulfate levels	0.04	7.26E-06
rs79101022	G	T	0.12	0.01	0.06	0.09	0.06	8.15E-01	Wet age-related macular degeneration	NA	5alpha-androstan-3beta,17beta-diol disulfate levels	0.03	8.47E-06
rs79293656	T	C	-0.24	0.04	0.02	0.02	0.1	6.98E-01	Wet age-related macular degeneration	NA	5alpha-androstan-3beta,17beta-diol disulfate levels	0.05	1.57E-06
rs9414945	C	T	0.09	-0.02	0.13	0.14	0.05	6.13E-01	Wet age-related macular	NA	5alpha-androstan	0.02	7.88E-06

		degeneration	-3beta,17beta-diol disulfate levels	
