# Supplementary material for: Causal association between metabolites and age-related macular degeneration: a bidirectional two-sample mendelian randomization study
Source: Hereditas. 2024 Dec 20;161:51. doi: 10.1186/s41065-024-00356-6 (PMC11662531; doi:10.1186/s41065-024-00356-6)
Supplement: Supplementary file 7 — Supplementary Material 7 [file 41065_2024_356_MOESM7_ESM.pdf]

Supplementary Table 4. SNPs for reverse MR analysis.

SNP	effect_allele	other_allele	beta.exposure	eaf.exposure	se.exposure	pval.exposure	exposure
rs10754199	G	A	-0.58	0.56	0.03	1.77E-113	Age-related macular degeneration (whether dry or wet)
rs11200630	C	T	0.83	0.24	0.03	4.16E-160	Age-related macular degeneration (whether dry or wet)
rs114003406	T	A	0.29	0.15	0.04	6.00E-16	Age-related macular degeneration (whether dry or wet)
rs145630012	G	T	-0.29	0.13	0.04	4.90E-14	Age-related macular degeneration (whether dry or wet)
rs2230199	C	G	0.21	0.18	0.03	5.47E-11	Age-related macular degeneration (whether dry or wet)
rs429608	A	G	-0.36	0.12	0.04	2.14E-19	Age-related macular degeneration (whether dry or wet)
rs4980260	T	G	0.15	0.38	0.03	2.42E-08	Age-related macular degeneration (whether dry or wet)
rs57257925	C	G	-0.19	0.19	0.03	3.63E-09	Age-related macular degeneration (whether dry or wet)
rs7478014	T	C	0.25	0.1	0.04	5.51E-09	Age-related macular degeneration (whether dry or wet)
rs10754199	G	A	-0.59	0.56	0.03	3.02E-80	Dry age-related macular degeneration (includes geographic atrophy)
rs11200630	C	T	0.77	0.24	0.04	7.61E-94	Dry age-related macular degeneration (includes geographic atrophy)
rs114003406	T	A	0.26	0.15	0.04	1.20E-09	Dry age-related macular degeneration (includes geographic atrophy)
rs145630012	G	T	-0.29	0.13	0.05	4.29E-10	Dry age-related macular degeneration (includes geographic atrophy)
rs429608	A	G	-0.36	0.12	0.05	7.14E-14	Dry age-related macular degeneration (includes geographic atrophy)
rs7531980	C	T	0.21	0.79	0.04	2.23E-08	Dry age-related macular degeneration (includes geographic atrophy)
rs10754199	G	A	-0.64	0.56	0.03	1.18E-81	Wet age-related macular degeneration
rs11200630	C	T	1.04	0.24	0.04	1.06E-140	Wet age-related macular degeneration
rs11248524	G	T	0.36	0.07	0.06	3.32E-08	Wet age-related macular degeneration
rs114003406	T	A	0.36	0.15	0.05	6.31E-15	Wet age-related macular degeneration
rs145630012	G	T	-0.32	0.13	0.05	1.61E-10	Wet age-related macular degeneration
rs2230199	C	G	0.24	0.18	0.04	1.75E-08	Wet age-related macular degeneration
rs429358	C	T	-0.26	0.18	0.05	5.34E-09	Wet age-related macular degeneration
rs429608	A	G	-0.41	0.12	0.05	4.31E-15	Wet age-related macular degeneration
rs78691732	C	T	0.75	0.02	0.11	2.65E-11	Wet age-related macular degeneration
