# Supplementary material for: Causal association between metabolites and age-related macular degeneration: a bidirectional two-sample mendelian randomization study
Source: Hereditas. 2024 Dec 20;161:51. doi: 10.1186/s41065-024-00356-6 (PMC11662531; doi:10.1186/s41065-024-00356-6)
Supplement: Supplementary file 8 — Supplementary Material 8 [file 41065_2024_356_MOESM8_ESM.pdf]

# Supplementary Table 5. MR results for causal effects of metabolites on AMD and subtypes.

| outcome | exposure | method | nsnp | b | se | pval | lo_ci | up_ci | or | or_lci95 | or_uci95 |
|---|---|---|---|---|---|---|---|---|---|---|---|
| Age-related macular degeneration | 1-stearoyl-GPE (18:0) levels | MR Egger | 34 | -0.36 | 0.13 | 0.009 | -0.62 | -0.11 | 0.69 | 0.54 | 0.9 |
| Age-related macular degeneration | 1-stearoyl-GPE (18:0) levels | Weighted median | 34 | -0.21 | 0.08 | 0.007 | -0.37 | -0.06 | 0.81 | 0.69 | 0.94 |
| Age-related macular degeneration | 1-stearoyl-GPE (18:0) levels | Inverse variance weighted | 34 | -0.21 | 0.06 | <0.001 | -0.31 | -0.1 | 0.81 | 0.73 | 0.91 |
| Age-related macular degeneration | 1-stearoyl-GPE (18:0) levels | Simple mode | 34 | -0.21 | 0.17 | 0.228 | -0.54 | 0.13 | 0.81 | 0.58 | 1.13 |
| Age-related macular degeneration | 1-stearoyl-GPE (18:0) levels | Weighted mode | 34 | -0.23 | 0.13 | 0.077 | -0.48 | 0.02 | 0.79 | 0.62 | 1.02 |
| Age-related macular degeneration | Gulonate levels | MR Egger | 21 | 0.25 | 0.2 | 0.224 | -0.14 | 0.65 | 1.29 | 0.87 | 1.91 |
| Age-related macular degeneration | Gulonate levels | Weighted median | 21 | 0.21 | 0.12 | 0.068 | -0.02 | 0.44 | 1.24 | 0.98 | 1.55 |
| Age-related macular degeneration | Gulonate levels | Inverse variance weighted | 21 | 0.26 | 0.08 | 0.001 | 0.1 | 0.42 | 1.3 | 1.11 | 1.52 |
| Age-related macular degeneration | Gulonate levels | Simple mode | 21 | 0.11 | 0.2 | 0.591 | -0.28 | 0.5 | 1.11 | 0.76 | 1.64 |
| Age-related macular degeneration | Gulonate levels | Weighted mode | 21 | 0.16 | 0.19 | 0.413 | -0.21 | 0.53 | 1.17 | 0.81 | 1.7 |
| Age-related macular degeneration | Androstenediol (3beta,17beta) monosulfate (1) levels | MR Egger | 31 | -0.08 | 0.11 | 0.436 | -0.29 | 0.12 | 0.92 | 0.75 | 1.13 |
| Age-related macular degeneration | Androstenediol (3beta,17beta) monosulfate (1) levels | Weighted median | 31 | -0.17 | 0.09 | 0.074 | -0.35 | 0.02 | 0.85 | 0.71 | 1.02 |
| Age-related macular degeneration | Androstenediol (3beta,17beta) monosulfate (1) levels | Inverse variance weighted | 31 | -0.18 | 0.06 | 0.004 | -0.29 | -0.06 | 0.84 | 0.75 | 0.94 |
| Age-related macular degeneration | Androstenediol (3beta,17beta) monosulfate (1) levels | Simple mode | 31 | -0.08 | 0.14 | 0.589 | -0.36 | 0.2 | 0.93 | 0.7 | 1.22 |
| Age-related macular | Androstenediol (3beta,17beta) | Weighted mode | 31 | -0.14 | 0.1 | 0.149 | -0.33 | 0.05 | 0.87 | 0.72 | 1.05 |

| | | | | | | | | | | |
|---|---|---|---|---|---|---|---|---|---|---|
| degeneration | monosulfate (1) levels | | | | | | | | | |
| Age-related macular degeneration | Mannonate levels | MR Egger | 20 | 0.14 | 0.04 | 0.002 | 0.07 | 0.21 | 1.15 | 1.07 | 1.24 |
| Age-related macular degeneration | Mannonate levels | Weighted median | 20 | 0.12 | 0.04 | 0.01 | 0.03 | 0.2 | 1.12 | 1.03 | 1.23 |
| Age-related macular degeneration | Mannonate levels | Inverse variance weighted | 20 | 0.1 | 0.03 | 0.002 | 0.04 | 0.17 | 1.11 | 1.04 | 1.18 |
| Age-related macular degeneration | Mannonate levels | Simple mode | 20 | 0.14 | 0.15 | 0.352 | -0.15 | 0.43 | 1.15 | 0.86 | 1.54 |
| Age-related macular degeneration | Mannonate levels | Weighted mode | 20 | 0.12 | 0.04 | 0.008 | 0.04 | 0.2 | 1.13 | 1.04 | 1.22 |
| Age-related macular degeneration | Stearoyl sphingomyelin (d18:1/18:0) levels | MR Egger | 35 | -0.18 | 0.1 | 0.08 | -0.37 | 0.02 | 0.84 | 0.69 | 1.02 |
| Age-related macular degeneration | Stearoyl sphingomyelin (d18:1/18:0) levels | Weighted median | 35 | -0.21 | 0.07 | 0.004 | -0.36 | -0.07 | 0.81 | 0.7 | 0.93 |
| Age-related macular degeneration | Stearoyl sphingomyelin (d18:1/18:0) levels | Inverse variance weighted | 35 | -0.17 | 0.05 | <0.001 | -0.27 | -0.08 | 0.84 | 0.76 | 0.93 |
| Age-related macular degeneration | Stearoyl sphingomyelin (d18:1/18:0) levels | Simple mode | 35 | -0.21 | 0.17 | 0.22 | -0.53 | 0.12 | 0.81 | 0.59 | 1.13 |
| Age-related macular degeneration | Stearoyl sphingomyelin (d18:1/18:0) levels | Weighted mode | 35 | -0.29 | 0.12 | 0.018 | -0.52 | -0.06 | 0.75 | 0.59 | 0.94 |
| Age-related macular degeneration | Xylose levels | MR Egger | 25 | -0.25 | 0.11 | 0.035 | -0.47 | -0.03 | 0.78 | 0.63 | 0.97 |
| Age-related macular degeneration | Xylose levels | Weighted median | 25 | -0.24 | 0.08 | 0.003 | -0.39 | -0.08 | 0.79 | 0.68 | 0.92 |
| Age-related macular degeneration | Xylose levels | Inverse variance weighted | 25 | -0.22 | 0.06 | <0.001 | -0.34 | -0.11 | 0.8 | 0.71 | 0.9 |
| Age-related macular degeneration | Xylose levels | Simple mode | 25 | -0.21 | 0.12 | 0.103 | -0.45 | 0.03 | 0.81 | 0.64 | 1.03 |
| Age-related macular degeneration | Xylose levels | Weighted mode | 25 | -0.23 | 0.1 | 0.029 | -0.42 | -0.04 | 0.8 | 0.66 | 0.97 |
| Age-related macular degeneration | X-11850 levels | MR Egger | 23 | -0.15 | 0.16 | 0.357 | -0.46 | 0.16 | 0.86 | 0.63 | 1.17 |

| | | | | | | | | | | | |
|---|---|---|---|---|---|---|---|---|---|---|---|
| Age-related macular degeneration | X-11850 levels | Weighted median | 23 | -0.15 | 0.09 | 0.103 | -0.34 | 0.03 | 0.86 | 0.71 | 1.03 |
| Age-related macular degeneration | X-11850 levels | Inverse variance weighted | 23 | -0.21 | 0.07 | 0.002 | -0.35 | -0.08 | 0.81 | 0.71 | 0.92 |
| Age-related macular degeneration | X-11850 levels | Simple mode | 23 | -0.1 | 0.19 | 0.623 | -0.48 | 0.28 | 0.91 | 0.62 | 1.33 |
| Age-related macular degeneration | X-11850 levels | Weighted mode | 23 | -0.08 | 0.17 | 0.624 | -0.42 | 0.25 | 0.92 | 0.66 | 1.28 |
| Dry age-related macular degeneration (includes geographic atrophy) | 1-stearoyl-GPE (18:0) levels | MR Egger | 34 | -0.38 | 0.14 | 0.013 | -0.67 | -0.1 | 0.68 | 0.51 | 0.91 |
| Dry age-related macular degeneration (includes geographic atrophy) | 1-stearoyl-GPE (18:0) levels | Weighted median | 34 | -0.27 | 0.09 | 0.005 | -0.45 | -0.08 | 0.77 | 0.64 | 0.92 |
| Dry age-related macular degeneration (includes geographic atrophy) | 1-stearoyl-GPE (18:0) levels | Inverse variance weighted | 34 | -0.24 | 0.06 | <0.001 | -0.36 | -0.12 | 0.79 | 0.7 | 0.89 |
| Dry age-related macular degeneration (includes geographic atrophy) | 1-stearoyl-GPE (18:0) levels | Simple mode | 34 | -0.22 | 0.18 | 0.25 | -0.58 | 0.15 | 0.81 | 0.56 | 1.16 |
| Dry age-related macular degeneration (includes geographic atrophy) | 1-stearoyl-GPE (18:0) levels | Weighted mode | 34 | -0.27 | 0.13 | 0.04 | -0.53 | -0.02 | 0.76 | 0.59 | 0.98 |
| Dry age-related macular degeneration (includes geographic atrophy) | X-11850 levels | MR Egger | 23 | -0.19 | 0.19 | 0.339 | -0.56 | 0.19 | 0.83 | 0.57 | 1.21 |
| Dry age-related macular degeneration (includes geographic atrophy) | X-11850 levels | Weighted median | 23 | -0.21 | 0.12 | 0.084 | -0.44 | 0.03 | 0.81 | 0.65 | 1.03 |
| Dry age-related macular degeneration (includes geographic atrophy) | X-11850 levels | Inverse variance weighted | 23 | -0.32 | 0.08 | <0.001 | -0.48 | -0.16 | 0.73 | 0.62 | 0.85 |
| Dry age-related macular | X-11850 levels | Simple mode | 23 | -0.16 | 0.24 | 0.52 | -0.63 | 0.31 | 0.85 | 0.53 | 1.37 |

| | | | | | | | | | | | |
|---|---|---|---|---|---|---|---|---|---|---|---|
| degeneration (includes geographic atrophy) | | | | | | | | | | | |
| Dry age-related macular degeneration (includes geographic atrophy) | X-11850 levels | Weighted mode | 23 | -0.16 | 0.23 | 0.507 | -0.61 | 0.3 | 0.85 | 0.54 | 1.35 |
| Wet age-related macular degeneration | DHEAS levels | MR Egger | 44 | -0.41 | 0.14 | 0.004 | -0.68 | -0.15 | 0.66 | 0.51 | 0.86 |
| Wet age-related macular degeneration | DHEAS levels | Weighted median | 44 | -0.48 | 0.11 | <0.001 | -0.69 | -0.26 | 0.62 | 0.5 | 0.77 |
| Wet age-related macular degeneration | DHEAS levels | Inverse variance weighted | 44 | -0.28 | 0.08 | <0.001 | -0.43 | -0.13 | 0.76 | 0.65 | 0.88 |
| Wet age-related macular degeneration | DHEAS levels | Simple mode | 44 | -0.41 | 0.2 | 0.05 | -0.8 | -0.01 | 0.67 | 0.45 | 0.99 |
| Wet age-related macular degeneration | DHEAS levels | Weighted mode | 44 | -0.46 | 0.11 | <0.001 | -0.68 | -0.23 | 0.63 | 0.51 | 0.79 |
| Wet age-related macular degeneration | 1-stearoyl-GPE (18:0) levels | MR Egger | 34 | -0.37 | 0.17 | 0.037 | -0.71 | -0.04 | 0.69 | 0.49 | 0.96 |
| Wet age-related macular degeneration | 1-stearoyl-GPE (18:0) levels | Weighted median | 34 | -0.19 | 0.1 | 0.055 | -0.39 | 0 | 0.82 | 0.67 | 1 |
| Wet age-related macular degeneration | 1-stearoyl-GPE (18:0) levels | Inverse variance weighted | 34 | -0.21 | 0.07 | 0.003 | -0.35 | -0.07 | 0.81 | 0.7 | 0.93 |
| Wet age-related macular degeneration | 1-stearoyl-GPE (18:0) levels | Simple mode | 34 | -0.11 | 0.23 | 0.645 | -0.56 | 0.34 | 0.9 | 0.57 | 1.41 |
| Wet age-related macular degeneration | 1-stearoyl-GPE (18:0) levels | Weighted mode | 34 | -0.47 | 0.18 | 0.012 | -0.82 | -0.13 | 0.62 | 0.44 | 0.88 |
| Wet age-related macular degeneration | 5alpha-androstan-3beta,17beta -diol disulfate levels | MR Egger | 28 | -0.22 | 0.09 | 0.024 | -0.41 | -0.04 | 0.8 | 0.67 | 0.96 |
| Wet age-related macular degeneration | 5alpha-androstan-3beta,17beta -diol disulfate levels | Weighted median | 28 | -0.29 | 0.08 | <0.001 | -0.46 | -0.13 | 0.74 | 0.63 | 0.88 |
| Wet age-related macular degeneration | 5alpha-androstan-3beta,17beta -diol disulfate levels | Inverse variance weighted | 28 | -0.22 | 0.06 | <0.001 | -0.34 | -0.1 | 0.8 | 0.71 | 0.91 |
| Wet age-related macular degeneration | 5alpha-androstan-3beta,17beta -diol disulfate levels | Simple mode | 28 | -0.23 | 0.21 | 0.286 | -0.63 | 0.18 | 0.8 | 0.53 | 1.2 |

| | | | | | | | | | | | | |
|---|---|---|---|---|---|---|---|---|---|---|---|---|
| Wet age-related macular degeneration | 5alpha-androstan-3beta,17beta -diol disulfate levels | Weighted mode | 28 | -0.29 | 0.08 | 0.002 | -0.45 | -0.12 | 0.75 | 0.64 | 0.88 |
| Wet age-related macular degeneration | 16a-hydroxy DHEA 3-sulfate levels | MR Egger | 22 | 0.19 | 0.07 | 0.009 | 0.06 | 0.32 | 1.21 | 1.06 | 1.38 |
| Wet age-related macular degeneration | 16a-hydroxy DHEA 3-sulfate levels | Weighted median | 22 | 0.19 | 0.05 | <0.001 | 0.09 | 0.29 | 1.21 | 1.09 | 1.34 |
| Wet age-related macular degeneration | 16a-hydroxy DHEA 3-sulfate levels | Inverse variance weighted | 22 | 0.18 | 0.05 | 0.001 | 0.08 | 0.29 | 1.2 | 1.08 | 1.33 |
| Wet age-related macular degeneration | 16a-hydroxy DHEA 3-sulfate levels | Simple mode | 22 | 0.18 | 0.18 | 0.33 | -0.17 | 0.52 | 1.19 | 0.84 | 1.68 |
| Wet age-related macular degeneration | 16a-hydroxy DHEA 3-sulfate levels | Weighted mode | 22 | 0.18 | 0.06 | 0.004 | 0.07 | 0.29 | 1.2 | 1.08 | 1.34 |
| Wet age-related macular degeneration | Androstenediol (3beta,17beta) monosulfate (1) levels | MR Egger | 31 | -0.27 | 0.14 | 0.062 | -0.53 | 0 | 0.77 | 0.59 | 1 |
| Wet age-related macular degeneration | Androstenediol (3beta,17beta) monosulfate (1) levels | Weighted median | 31 | -0.51 | 0.12 | <0.001 | -0.74 | -0.28 | 0.6 | 0.48 | 0.76 |
| Wet age-related macular degeneration | Androstenediol (3beta,17beta) monosulfate (1) levels | Inverse variance weighted | 31 | -0.29 | 0.08 | <0.001 | -0.44 | -0.14 | 0.75 | 0.64 | 0.87 |
| Wet age-related macular degeneration | Androstenediol (3beta,17beta) monosulfate (1) levels | Simple mode | 31 | -0.54 | 0.24 | 0.028 | -1.01 | -0.08 | 0.58 | 0.37 | 0.92 |
| Wet age-related macular degeneration | Androstenediol (3beta,17beta) monosulfate (1) levels | Weighted mode | 31 | -0.53 | 0.13 | <0.001 | -0.79 | -0.27 | 0.59 | 0.46 | 0.76 |
| Wet age-related macular degeneration | Succinimide levels | MR Egger | 21 | 0.15 | 0.1 | 0.145 | -0.04 | 0.33 | 1.16 | 0.96 | 1.4 |
| Wet age-related macular degeneration | Succinimide levels | Weighted median | 21 | 0.18 | 0.08 | 0.022 | 0.03 | 0.33 | 1.19 | 1.03 | 1.39 |
| Wet age-related macular degeneration | Succinimide levels | Inverse variance weighted | 21 | 0.21 | 0.06 | 0.001 | 0.08 | 0.33 | 1.23 | 1.09 | 1.4 |
| Wet age-related macular degeneration | Succinimide levels | Simple mode | 21 | 0.25 | 0.11 | 0.043 | 0.02 | 0.47 | 1.28 | 1.02 | 1.6 |
| Wet age-related macular degeneration | Succinimide levels | Weighted mode | 21 | 0.16 | 0.07 | 0.038 | 0.02 | 0.31 | 1.18 | 1.02 | 1.36 |
| Wet age-related macular | Xylose levels | MR Egger | 25 | -0.33 | 0.15 | 0.035 | -0.61 | -0.04 | 0.72 | 0.54 | 0.96 |

| | | | | | | | | | | | |
|---|---|---|---|---|---|---|---|---|---|---|---|
| degeneration | | | | | | | | | | | |
| Wet age-related macular degeneration | Xylose levels | Weighted median | 25 | -0.34 | 0.1 | 0.001 | -0.53 | -0.14 | 0.71 | 0.59 | 0.87 |
| Wet age-related macular degeneration | Xylose levels | Inverse variance weighted | 25 | -0.33 | 0.08 | <0.001 | -0.48 | -0.18 | 0.72 | 0.62 | 0.84 |
| Wet age-related macular degeneration | Xylose levels | Simple mode | 25 | -0.31 | 0.15 | 0.047 | -0.6 | -0.02 | 0.73 | 0.55 | 0.98 |
| Wet age-related macular degeneration | Xylose levels | Weighted mode | 25 | -0.34 | 0.13 | 0.018 | -0.6 | -0.08 | 0.71 | 0.55 | 0.93 |
| Wet age-related macular degeneration | X-13553 levels | MR Egger | 24 | 0.33 | 0.15 | 0.035 | 0.04 | 0.61 | 1.38 | 1.04 | 1.84 |
| Wet age-related macular degeneration | X-13553 levels | Weighted median | 24 | 0.24 | 0.13 | 0.065 | -0.02 | 0.49 | 1.27 | 0.99 | 1.62 |
| Wet age-related macular degeneration | X-13553 levels | Inverse variance weighted | 24 | 0.31 | 0.08 | <0.001 | 0.14 | 0.47 | 1.36 | 1.15 | 1.6 |
| Wet age-related macular degeneration | X-13553 levels | Simple mode | 24 | 0.25 | 0.19 | 0.205 | -0.13 | 0.63 | 1.29 | 0.88 | 1.88 |
| Wet age-related macular degeneration | X-13553 levels | Weighted mode | 24 | 0.25 | 0.15 | 0.108 | -0.04 | 0.55 | 1.29 | 0.96 | 1.73 |
| Wet age-related macular degeneration | N2-acetyl,N6,N6-dimethyllysine levels | MR Egger | 24 | -0.09 | 0.04 | 0.032 | -0.17 | -0.01 | 0.91 | 0.85 | 0.99 |
| Wet age-related macular degeneration | N2-acetyl,N6,N6-dimethyllysine levels | Weighted median | 24 | -0.1 | 0.03 | 0.002 | -0.17 | -0.04 | 0.9 | 0.85 | 0.96 |
| Wet age-related macular degeneration | N2-acetyl,N6,N6-dimethyllysine levels | Inverse variance weighted | 24 | -0.09 | 0.03 | 0.002 | -0.15 | -0.03 | 0.91 | 0.86 | 0.97 |
| Wet age-related macular degeneration | N2-acetyl,N6,N6-dimethyllysine levels | Simple mode | 24 | -0.02 | 0.11 | 0.88 | -0.23 | 0.19 | 0.98 | 0.8 | 1.22 |
| Wet age-related macular degeneration | N2-acetyl,N6,N6-dimethyllysine levels | Weighted mode | 24 | -0.1 | 0.03 | 0.005 | -0.16 | -0.03 | 0.91 | 0.85 | 0.97 |
