# Supplementary material for: Causal association between metabolites and age-related macular degeneration: a bidirectional two-sample mendelian randomization study
Source: Hereditas. 2024 Dec 20;161:51. doi: 10.1186/s41065-024-00356-6 (PMC11662531; doi:10.1186/s41065-024-00356-6)
Supplement: Supplementary file 9 — Supplementary Material 9 [file 41065_2024_356_MOESM9_ESM.pdf]

Supplementary Table 6. MR results for causal effects of AMD and subtypes on metabolites.

outcome	exposure	method	nsnp	b	se	pval	lo_ci	up_ci	or	or_lci95	or_uci95
1-stearoyl-GPE (18:0)	Age-related macular degeneration (whether dry or wet)	MR Egger	9	-0.03	0.03	0.408	-0.09	0.03	0.97	0.91	1.04
1-stearoyl-GPE (18:0)	Age-related macular degeneration (whether dry or wet)	Weighted median	9	0	0.02	0.898	-0.04	0.03	1	0.96	1.03
1-stearoyl-GPE (18:0)	Age-related macular degeneration (whether dry or wet)	Inverse variance weighted	9	-0.01	0.02	0.749	-0.04	0.03	0.99	0.96	1.03
1-stearoyl-GPE (18:0)	Age-related macular degeneration (whether dry or wet)	Simple mode	9	0.02	0.04	0.532	-0.05	0.1	1.02	0.95	1.1
1-stearoyl-GPE (18:0)	Age-related macular degeneration (whether dry or wet)	Weighted mode	9	0	0.02	0.943	-0.04	0.03	1	0.96	1.04
Gulonate	Age-related macular degeneration (whether dry or wet)	MR Egger	9	0.01	0.03	0.735	-0.05	0.07	1.01	0.96	1.07
Gulonate	Age-related macular degeneration (whether dry or wet)	Weighted median	9	0.01	0.02	0.586	-0.03	0.05	1.01	0.97	1.05
Gulonate	Age-related macular degeneration (whether dry or wet)	Inverse variance weighted	9	0	0.01	0.868	-0.03	0.03	1	0.97	1.03
Gulonate	Age-related macular degeneration (whether dry or wet)	Simple mode	9	0.02	0.03	0.578	-0.05	0.08	1.02	0.96	1.09
Gulonate	Age-related macular degeneration (whether dry or	Weighted mode	9	0.01	0.02	0.712	-0.03	0.04	1.01	0.97	1.04

	wet)										
Androstenediol (3beta,17beta) monosulfate	Age-related macular degeneration (whether dry or wet)	MR Egger	9	-0.03	0.03	0.352	-0.08	0.03	0.97	0.93	1.03
Androstenediol (3beta,17beta) monosulfate	Age-related macular degeneration (whether dry or wet)	Weighted median	9	-0.04	0.02	0.007	-0.07	-0.01	0.96	0.93	0.99
Androstenediol (3beta,17beta) monosulfate	Age-related macular degeneration (whether dry or wet)	Inverse variance weighted	9	-0.04	0.01	0.003	-0.07	-0.01	0.96	0.94	0.99
Androstenediol (3beta,17beta) monosulfate	Age-related macular degeneration (whether dry or wet)	Simple mode	9	-0.07	0.03	0.039	-0.13	-0.01	0.93	0.88	0.99
Androstenediol (3beta,17beta) monosulfate	Age-related macular degeneration (whether dry or wet)	Weighted mode	9	-0.04	0.02	0.036	-0.07	-0.01	0.96	0.93	0.99
Mannonate	Age-related macular degeneration (whether dry or wet)	MR Egger	9	0.01	0.03	0.805	-0.06	0.08	1.01	0.94	1.08
Mannonate	Age-related macular degeneration (whether dry or wet)	Weighted median	9	-0.01	0.02	0.563	-0.04	0.02	0.99	0.96	1.02
Mannonate	Age-related macular degeneration (whether dry or wet)	Inverse variance weighted	9	-0.01	0.02	0.763	-0.04	0.03	0.99	0.96	1.03
Mannonate	Age-related macular degeneration (whether dry or wet)	Simple mode	9	0.04	0.03	0.307	-0.03	0.1	1.04	0.97	1.11
Mannonate	Age-related macular degeneration (whether dry or wet)	Weighted mode	9	-0.01	0.02	0.688	-0.04	0.03	0.99	0.96	1.03
Stearoyl sphingomyelin (d18:1/18:0)	Age-related macular degeneration (whether dry or	MR Egger	9	0.05	0.03	0.14	-0.01	0.11	1.05	0.99	1.11

	wet)									
Stearoyl sphingomyelin (d18:1/18:0)	Age-related macular degeneration (whether dry or wet)	Weighted median	9	0.03	0.02	0.087	0	0.06	1.03	1	1.07
Stearoyl sphingomyelin (d18:1/18:0)	Age-related macular degeneration (whether dry or wet)	Inverse variance weighted	9	0.03	0.02	0.042	0	0.06	1.03	1	1.06
Stearoyl sphingomyelin (d18:1/18:0)	Age-related macular degeneration (whether dry or wet)	Simple mode	9	0.05	0.03	0.146	-0.01	0.11	1.05	0.99	1.12
Stearoyl sphingomyelin (d18:1/18:0)	Age-related macular degeneration (whether dry or wet)	Weighted mode	9	0.03	0.02	0.117	0	0.07	1.03	1	1.07
Xylose	Age-related macular degeneration (whether dry or wet)	MR Egger	9	0.03	0.03	0.337	-0.03	0.1	1.03	0.97	1.1
Xylose	Age-related macular degeneration (whether dry or wet)	Weighted median	9	0.03	0.02	0.115	-0.01	0.08	1.04	0.99	1.08
Xylose	Age-related macular degeneration (whether dry or wet)	Inverse variance weighted	9	0.02	0.02	0.38	-0.02	0.05	1.02	0.98	1.05
Xylose	Age-related macular degeneration (whether dry or wet)	Simple mode	9	-0.01	0.04	0.896	-0.08	0.07	0.99	0.92	1.07
Xylose	Age-related macular degeneration (whether dry or wet)	Weighted mode	9	0.02	0.02	0.485	-0.03	0.06	1.02	0.97	1.06
X-11850	Age-related macular degeneration (whether dry or wet)	MR Egger	9	0.07	0.04	0.139	-0.01	0.14	1.07	0.99	1.15
X-11850	Age-related macular degeneration (whether dry or	Weighted median	9	0.01	0.02	0.638	-0.03	0.05	1.01	0.97	1.05

	wet)										
X-11850	Age-related macular degeneration (whether dry or wet)	Inverse variance weighted	9	0	0.02	0.927	-0.05	0.04	1	0.95	1.05
X-11850	Age-related macular degeneration (whether dry or wet)	Simple mode	9	0	0.04	0.903	-0.08	0.07	1	0.93	1.07
X-11850	Age-related macular degeneration (whether dry or wet)	Weighted mode	9	0.01	0.02	0.463	-0.02	0.05	1.01	0.98	1.05
1-stearoyl-GPE (18:0)	Dry age-related macular degeneration (includes geographic atrophy)	MR Egger	6	0.02	0.05	0.674	-0.08	0.12	1.02	0.93	1.13
1-stearoyl-GPE (18:0)	Dry age-related macular degeneration (includes geographic atrophy)	Weighted median	6	0	0.02	0.797	-0.04	0.03	1	0.96	1.03
1-stearoyl-GPE (18:0)	Dry age-related macular degeneration (includes geographic atrophy)	Inverse variance weighted	6	-0.01	0.02	0.456	-0.05	0.02	0.99	0.95	1.02
1-stearoyl-GPE (18:0)	Dry age-related macular degeneration (includes geographic atrophy)	Simple mode	6	-0.09	0.03	0.052	-0.15	-0.02	0.92	0.86	0.98
1-stearoyl-GPE (18:0)	Dry age-related macular degeneration (includes geographic atrophy)	Weighted mode	6	0	0.02	0.86	-0.04	0.03	1	0.96	1.04
X-11850	Dry age-related macular degeneration (includes geographic atrophy)	MR Egger	6	0.13	0.05	0.049	0.04	0.22	1.14	1.04	1.25
X-11850	Dry age-related macular degeneration (includes geographic atrophy)	Weighted median	6	0.01	0.02	0.657	-0.03	0.05	1.01	0.97	1.06
X-11850	Dry age-related macular degeneration (includes	Inverse variance	6	0	0.03	0.984	-0.06	0.06	1	0.94	1.06

	geographic atrophy)	weighted									
X-11850	Dry age-related macular degeneration (includes geographic atrophy)	Simple mode	6	-0.02	0.04	0.69	-0.08	0.05	0.99	0.92	1.06
X-11850	Dry age-related macular degeneration (includes geographic atrophy)	Weighted mode	6	0.01	0.02	0.525	-0.02	0.05	1.01	0.98	1.05
DHEAS	Wet age-related macular degeneration	MR Egger	9	-0.04	0.04	0.322	-0.11	0.03	0.96	0.9	1.03
DHEAS	Wet age-related macular degeneration	Weighted median	9	-0.02	0.01	0.102	-0.05	0	0.98	0.95	1
DHEAS	Wet age-related macular degeneration	Inverse variance weighted	9	-0.02	0.02	0.24	-0.05	0.01	0.98	0.95	1.01
DHEAS	Wet age-related macular degeneration	Simple mode	9	-0.01	0.02	0.683	-0.06	0.04	0.99	0.95	1.04
DHEAS	Wet age-related macular degeneration	Weighted mode	9	-0.02	0.01	0.181	-0.05	0.01	0.98	0.95	1.01
1-stearoyl-GPE (18:0)	Wet age-related macular degeneration	MR Egger	9	0.03	0.06	0.588	-0.08	0.15	1.03	0.92	1.16
1-stearoyl-GPE (18:0)	Wet age-related macular degeneration	Weighted median	9	0	0.01	0.82	-0.03	0.03	1	0.97	1.03
1-stearoyl-GPE (18:0)	Wet age-related macular degeneration	Inverse variance weighted	9	-0.02	0.03	0.563	-0.07	0.04	0.98	0.93	1.04
1-stearoyl-GPE (18:0)	Wet age-related macular degeneration	Simple mode	9	-0.01	0.03	0.818	-0.06	0.05	0.99	0.94	1.05
1-stearoyl-GPE (18:0)	Wet age-related macular degeneration	Weighted mode	9	0	0.01	0.812	-0.03	0.02	1	0.97	1.02
5alpha-androstan-3beta,17beta-diol disulfate	Wet age-related macular degeneration	MR Egger	9	0	0.03	0.911	-0.05	0.06	1	0.95	1.06
5alpha-androstan-3beta,17beta-diol disulfate	Wet age-related macular degeneration	Weighted median	9	-0.01	0.01	0.638	-0.03	0.02	0.99	0.97	1.02

5alpha-androstan-3beta,17beta-diol disulfate	Wet age-related macular degeneration	Inverse variance weighted	9	-0.01	0.01	0.387	-0.04	0.01	0.99	0.97	1.01
5alpha-androstan-3beta,17beta-diol disulfate	Wet age-related macular degeneration	Simple mode	9	0.01	0.02	0.662	-0.04	0.06	1.01	0.96	1.06
5alpha-androstan-3beta,17beta-diol disulfate	Wet age-related macular degeneration	Weighted mode	9	-0.01	0.01	0.38	-0.04	0.01	0.99	0.96	1.01
16a-hydroxy DHEA 3-sulfate	Wet age-related macular degeneration	MR Egger	9	-0.03	0.03	0.411	-0.09	0.04	0.97	0.91	1.04
16a-hydroxy DHEA 3-sulfate	Wet age-related macular degeneration	Weighted median	9	-0.02	0.01	0.177	-0.05	0.01	0.98	0.95	1.01
16a-hydroxy DHEA 3-sulfate	Wet age-related macular degeneration	Inverse variance weighted	9	-0.01	0.01	0.519	-0.04	0.02	0.99	0.96	1.02
16a-hydroxy DHEA 3-sulfate	Wet age-related macular degeneration	Simple mode	9	-0.01	0.02	0.573	-0.06	0.03	0.99	0.94	1.03
16a-hydroxy DHEA 3-sulfate	Wet age-related macular degeneration	Weighted mode	9	-0.01	0.01	0.356	-0.04	0.01	0.99	0.96	1.01
Androstenediol (3beta,17beta) monosulfate	Wet age-related macular degeneration	MR Egger	9	-0.05	0.03	0.139	-0.11	0.01	0.95	0.89	1.01
Androstenediol (3beta,17beta) monosulfate	Wet age-related macular degeneration	Weighted median	9	-0.03	0.01	0.01	-0.06	-0.01	0.97	0.94	0.99
Androstenediol (3beta,17beta) monosulfate	Wet age-related macular degeneration	Inverse variance weighted	9	-0.03	0.01	0.074	-0.05	0	0.97	0.95	1
Androstenediol (3beta,17beta) monosulfate	Wet age-related macular degeneration	Simple mode	9	-0.03	0.02	0.156	-0.08	0.01	0.97	0.93	1.01
Androstenediol (3beta,17beta) monosulfate	Wet age-related macular degeneration	Weighted mode	9	-0.03	0.01	0.043	-0.06	-0.01	0.97	0.94	0.99
Succinimide	Wet age-related macular degeneration	MR Egger	9	-0.07	0.03	0.061	-0.14	-0.01	0.93	0.87	0.99
Succinimide	Wet age-related macular degeneration	Weighted median	9	-0.02	0.02	0.358	-0.05	0.02	0.98	0.95	1.02

Succinimide	Wet age-related macular degeneration	Inverse variance weighted	9	0	0.02	0.929	-0.04	0.04	1	0.96	1.04
Succinimide	Wet age-related macular degeneration	Simple mode	9	0.02	0.03	0.557	-0.05	0.09	1.02	0.96	1.09
Succinimide	Wet age-related macular degeneration	Weighted mode	9	-0.01	0.02	0.465	-0.05	0.02	0.99	0.95	1.02
Xylose	Wet age-related macular degeneration	MR Egger	9	0.04	0.04	0.41	-0.05	0.12	1.04	0.95	1.13
Xylose	Wet age-related macular degeneration	Weighted median	9	0.01	0.02	0.671	-0.03	0.04	1.01	0.97	1.04
Xylose	Wet age-related macular degeneration	Inverse variance weighted	9	0.01	0.02	0.671	-0.03	0.05	1.01	0.97	1.05
Xylose	Wet age-related macular degeneration	Simple mode	9	0.01	0.03	0.707	-0.05	0.07	1.01	0.96	1.07
Xylose	Wet age-related macular degeneration	Weighted mode	9	0.02	0.02	0.357	-0.02	0.05	1.02	0.98	1.05
X-13553	Wet age-related macular degeneration	MR Egger	9	-0.01	0.03	0.594	-0.06	0.04	0.99	0.94	1.04
X-13553	Wet age-related macular degeneration	Weighted median	9	0.03	0.02	0.089	0	0.06	1.03	1	1.06
X-13553	Wet age-related macular degeneration	Inverse variance weighted	9	0.02	0.01	0.134	-0.01	0.04	1.02	0.99	1.04
X-13553	Wet age-related macular degeneration	Simple mode	9	0.05	0.03	0.12	-0.01	0.1	1.05	0.99	1.11
X-13553	Wet age-related macular degeneration	Weighted mode	9	0	0.02	0.845	-0.03	0.03	1	0.97	1.03
N2-acetyl,N6,N6-dimethyllysine	Wet age-related macular degeneration	MR Egger	9	0	0.04	0.941	-0.07	0.07	1	0.93	1.07
N2-acetyl,N6,N6-dimethyllysine	Wet age-related macular degeneration	Weighted median	9	0.01	0.02	0.454	-0.02	0.04	1.01	0.98	1.04

N2-acetyl,N6,N6-dimethyllysine	Wet age-related macular degeneration	Inverse variance weighted	9	0.01	0.02	0.645	-0.02	0.04	1.01	0.98	1.04
N2-acetyl,N6,N6-dimethyllysine	Wet age-related macular degeneration	Simple mode	9	0.01	0.03	0.627	-0.04	0.06	1.01	0.96	1.07
N2-acetyl,N6,N6-dimethyllysine	Wet age-related macular degeneration	Weighted mode	9	0.01	0.02	0.464	-0.02	0.04	1.01	0.98	1.04
