# Supplementary material for: Causal association between metabolites and age-related macular degeneration: a bidirectional two-sample mendelian randomization study
Source: Hereditas. 2024 Dec 20;161:51. doi: 10.1186/s41065-024-00356-6 (PMC11662531; doi:10.1186/s41065-024-00356-6)
Supplement: Supplementary file 10 — Supplementary Material 10 [file 41065_2024_356_MOESM10_ESM.pdf]

Supplementary Table 7. Sensitivity analysis of reverse MR analysis.

exposure	outcome	method	Cochran's Q test		MR-Egger intercept test			MR-PRESSO Global test	
			Q	Q_pval	intercept	se	P value	RSSobs	P value
AMD	1-stearoyl-GPE (18:0)	MR Egger	8.39	0.300	0.01	0.01	0.429	10.62	0.458
		Inverse variance weighted	9.23	0.323					
	Gulonate	MR Egger	5.83	0.559	0.00	0.01	0.765	8.80	0.654
		Inverse variance weighted	5.93	0.655					
	Androstenediol (3beta,17beta) monosulfate	MR Egger	5.74	0.570	-0.01	0.01	0.557	6.99	0.746
		Inverse variance weighted	6.12	0.633					
	Mannonate	MR Egger	9.95	0.192	-0.01	0.01	0.649	11.50	0.377
		Inverse variance weighted	10.27	0.247					
	Stearoyl sphingomyelin (d18:1/18:0)	MR Egger	4.75	0.691	-0.01	0.01	0.504	5.74	0.826
		Inverse variance weighted	5.24	0.731					
	Xylose	MR Egger	6.85	0.445	-0.01	0.01	0.523	10.73	0.531
		Inverse variance weighted	7.30	0.505					
	X-11850	MR Egger	11.46	0.120	-0.03	0.02	0.083	28.91	0.099
		Inverse variance weighted	18.15	0.020					
Dry AMD	1-stearoyl-GPE (18:0)	MR Egger	6.02	0.198	-0.02	0.03	0.465	9.09	0.345
		Inverse variance weighted	7.00	0.221					
	X-11850	MR Egger	4.61	0.330	-0.07	0.02	0.037	25.60	0.129
		Inverse variance weighted	15.49	0.008					
Wet AMD	DHEAS	MR Egger	15.40	0.031	0.01	0.02	0.561	18.21	0.134
		Inverse variance weighted	16.22	0.039					
	1-stearoyl-GPE (18:0)	MR Egger	9.62	0.141	0.01	0.02	0.816	11.09	0.322
		Inverse variance weighted	9.72	0.205					
	5alpha-androstan-3beta,17beta-diol disulfate	MR Egger	9.80	0.200	-0.01	0.02	0.589	13.06	0.376
		Inverse variance weighted	10.25	0.248					
	16a-hydroxy DHEA 3-sulfate	MR Egger	10.81	0.147	0.01	0.02	0.530	13.26	0.321
		Inverse variance weighted	11.48	0.176					
	Androstenediol	MR Egger	11.58	0.115	0.02	0.02	0.375	14.86	0.244

	(3beta,17beta) monosulfate	Inverse variance weighted	13.07	0.109					
	Succinimide	MR Egger	6.45	0.488	0.05	0.02	0.043	15.26	0.239
		Inverse variance weighted	12.51	0.130					
	Xylose	MR Egger	13.92	0.053	-0.02	0.02	0.465	20.23	0.157
		Inverse variance weighted	15.11	0.057					
	X-13553	MR Egger	5.28	0.626	0.02	0.01	0.198	13.21	0.495
		Inverse variance weighted	7.30	0.505					
	N2-acetyl,N6,N6-dimethyllysine	MR Egger	10.74	0.150	0.01	0.02	0.761	11.82	0.355
		Inverse variance weighted	10.89	0.208					
