# Supplementary material for: Causal association between metabolites and age-related macular degeneration: a bidirectional two-sample mendelian randomization study
Source: Hereditas. 2024 Dec 20;161:51. doi: 10.1186/s41065-024-00356-6 (PMC11662531; doi:10.1186/s41065-024-00356-6)
Supplement: Supplementary file 13 — Supplementary Material 13 [file 41065_2024_356_MOESM13_ESM.docx]

**supplementary materials about five pitfalls**

A recent study by Burgess et al. (BMC Medicine, 2024) highlighted five pitfalls. Following is how our study avoided the five pitfalls.

1. Inappropriate research question

The pathogenesis of AMD is unclear, and metabolites have shown a possible association with AMD, but the causal relationship is less clear. Evidence of randomized controlled trial (RCT) is lacking. Previous MR studies took metabolites as exposure and diseases as outcome and obtained some possible causal relationships. (e. g., PMID: 35692035 and PMID: 34735824). Based on the lack of knowledge in the current literature and clinical needs, we chose this research question, without selecting for exposure factors impractical or unrelated to genetic variants as mentioned in the article by Burgess et al, for example, use of chopsticks.

1. Inappropriate choice of variants as instruments

Our screening of instrumental variables was based on the GWAS of an external dataset (PMID: 36635386). In 2022, an article entitled "Genomic Atlas of the plasma metabolome priorities metabolites implied in human diseases" was published in Nature Genetics (PMID: 36635386). The authors analyzed the proportion of 1091 blood metabolites and 309 metabolites by GWAS, and provided publicly available summary statistics in 2023, so we can screen the required instrumental variables. Our selection of instrumental variables adheres to the STROBE-MR guidelines and the three core assumption of mendelian randomization. We chose SNPs that are strongly associated with metabolites as instrumental variables, excluded SNPs in linkage disequilibrium, calculated the F-value for each SNP, and removed confounding SNPs related to known risk factors for AMD. This aligns with the assumptions of instrumental variables in mendelian randomization, where the instrumental variables must be strongly related to the exposure and should affect the outcome only through the exposure.

1. Insufficient interrogation of findings

In this study, we employed random-effect inverse variance weighted (IVW) as the primary analytical method for MR analysis and performed false discovery rate (FDR) correction on the P-values. Additionally, we utilized MR-Egger, weighted median, weighted mode, and simple mode as supplementary analytical methods to ensure that the results obtained from these five methods are directionally consistent, thereby supporting the findings from IVW. Beyond these, we conducted sensitivity analyses, including the MR-Egger intercept, Cochran’s Q, MR-PRESSO test, and leave-one-out analysis, to ensure the robustness of our results.

1. Inappropriate interpretation of findings

We have cautiously interpreted the results of MR analysis, ensuring that our conclusions are based on the data and analysis, and avoiding overinterpretation. We propose the potential impact of metabolites on AMD and suggest further mechanistic studies to explore the utility of these metabolites as biomarkers or therapeutic targets. Future cohort studies are still needed to confirm the causal associations between specific metabolites and macular degeneration, as Burgess et al. (PMID: 39256834) have stated, the specific mechanisms underlying the causal relationships between metabolites and outcomes remain to be investigated, which we have also noted in our manuscript.

1. Lack of engagement with previous literature

In the background section of our article, we have mentioned the epidemiology, genetics, and environmental factors of AMD, as well as the potential role of metabolites in pathogenesis. We have delved into the existing literature to ensure that our research builds upon current knowledge. We have compared our findings with the existing body of literature and discussed in detail how our study fills gaps in knowledge, such as previous discoveries highlighting the significant role of lipids in macular degeneration, while our study reveals a causal association between various lipid substances and AMD. This suggests that further investigation into the mechanisms of action of these substances in AMD could be a feasible direction for future research.
