# Supplementary material for: Causal association between metabolites and age-related macular degeneration: a bidirectional two-sample mendelian randomization study
Source: Hereditas. 2024 Dec 20;161:51. doi: 10.1186/s41065-024-00356-6 (PMC11662531; doi:10.1186/s41065-024-00356-6)
Supplement: Supplementary file 14 — Supplementary Material 14 [file 41065_2024_356_MOESM14_ESM.docx]

**Supplementary materials about analysis codes**

exposureFile="exposure.F.csv"

outcomeName="AMD"

exposure_dat=read_exposure_data(filename=exposureFile,sep = ",",snp_col = "SNP",beta_col = "beta.exposure",se_col = "se.exposure",pval_col = "pval.exposure",effect_allele_col="effect_allele.exposure",other_allele_col = "other_allele.exposure",eaf_col = "eaf.exposure",phenotype_col = "exposure",id_col = "id.exposure",samplesize_col = "samplesize.exposure",chr_col="chr.exposure", pos_col = "pos.exposure",clump=FALSE)

outcomeData=extract_outcome_data(snps=exposure_dat$SNP, outcomes=“ID”)

write.csv(outcomeData, file="outcome.csv", row.names=F)

outcomeData$outcome=outcomeName

dat=harmonise_data(exposure_dat, outcomeData)

outTab=dat[dat$mr_keep=="TRUE",]

write.csv(outTab, file="table.SNP.csv", row.names=F)

mrResult=mr(dat)

mrTab=generate_odds_ratios(mrResult)

write.csv(mrTab, file="table.MRresult.csv", row.names=F)

heterTab=mr_heterogeneity(dat)

write.csv(heterTab, file="table.heterogeneity.csv", row.names=F)

pleioTab=mr_pleiotropy_test(dat)

write.csv(pleioTab, file="table.pleiotropy.csv", row.names=F)
